# Early‐Life Polyethylene Microplastic Exposure Exacerbates ADHD‐Like Behaviors in Mice via PI3K/AKT1/Nrf2 Signaling: Evidence From the Pilot Cohort and Multi‐Omics Analyses

**DOI:** 10.1002/advs.78594

**Published:** 2026-11-01

**Authors:** Yufan Luo, Ruijin Xie, Hua Xu, Xue Xiao, Wei Xiao, Qiyang Pan, Shengjie Xu, Li Liu, Zeliang Xiao, Yue Zhou, Chenyu Sun, Yu Wu, Yueying Liu

**Affiliations:** ^1^ Department of Pediatrics Affiliated Hospital of Jiangnan University Wuxi Jiangsu China; ^2^ Lab of Modern Environmental Toxicology Public Health and Preventive Medicine Wuxi School of Medicine Jiangnan University Wuxi Jiangsu China; ^3^ Department of Psychiatry and Psychology Mayo Clinic Rochester Minnesota USA; ^4^ Mayo Clinic School of Graduate Medical Education Mayo Clinic College of Medicine and Science Rochester Minnesota USA; ^5^ Department of Internal Medicine The Second People's Hospital of Hefei Hefei Anhui China; ^6^ School of Public Health University of Minnesota‐Twin Cities Minneapolis USA; ^7^ Division of Public Health Infectious Diseases and Occupational Medicine Mayo Clinic Rochester Minnesota USA

**Keywords:** adhd symptoms, environmental exposure, lipid peroxidation, melatonin, microplastics, multi omics, neurotoxicity, nfe2l2, pi3k signaling, plastic pollution

## Abstract

Microplastic pollution may influence neurodevelopment, yet its relationship with attention‐deficit/hyperactivity disorder (ADHD) remains unclear. We integrated a pediatric cohort, multi‐omics analyses, network toxicology, and animal experiments to investigate polyethylene (PE) microplastic exposure in ADHD vulnerability. Among 90 children, PE was the predominant polymer detected in blood and was more abundant in children with ADHD. Higher PE burden was associated with greater symptom severity, increased IL‐6, and reduced antioxidant capacity. Multi‐omics and network analyses converged on PI3K/AKT1/Nrf2 signaling. In a perinatal nicotine mouse model, developmental PE co‐exposure exacerbated ADHD‐like behaviors, dopaminergic dysfunction, and oxidative injury. Melatonin partially alleviated these abnormalities by restoring PI3K/AKT1/GSK3β and Nrf2 signaling, whereas AAV‐mediated PI3K knockdown weakened its protective effects. Collectively, the human findings identify circulating PE burden as an environmental correlate of ADHD‐related neurodevelopmental vulnerability, while the animal experiments provide evidence that developmental PE co‐exposure can aggravate ADHD‐like abnormalities in a susceptible background.

## Graphical abstract

Circulating polyethylene microplastic burden is positively associated with heightened ADHD symptom severity and systemic redox‐inflammatory imbalance in children. Mechanistic investigation in a developmental co‐exposure mouse model further demonstrates that microplastics aggravate ADHD‐like behaviors, dopaminergic dysregulation, and oxidative hippocampal injury. PI3K/AKT1/Nrf2 signaling is identified as a central mechanistic nexus linking microplastic‐induced neurotoxicity to neurodevelopmental vulnerability. Pharmacological modulation with melatonin partially restores pathway activity and ameliorates neurobehavioral deficits, establishing a modifiable therapeutic target for mitigating microplastic‐related developmental neurotoxicity. These findings integrate clinical association with experimental causation, supporting microplastic exposure as an emerging environmental risk factor for ADHD pathogenesis.

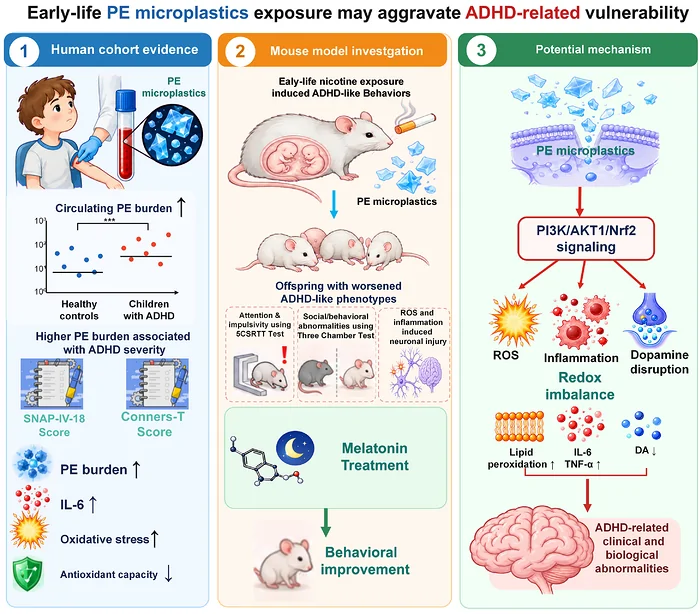

## 1 Introduction

With the rapid expansion of industrial production, humans now inhabit an environment permeated by plastic materials. Microplastics, commonly characterized as plastic fragments measuring less than 5 mm in diameter, have become pervasive pollutants detected across food supplies, potable water sources, atmospheric compartments, and the wider biosphere [1]. Human exposure occurs through ingestion, inhalation, and, potentially, dermal contact, and is now documented across the entire lifespan: microplastics have been quantified in human blood, placenta, and, most recently, brain tissue [2]. Children may be particularly vulnerable owing to their developing physiological barriers, immature antioxidant defense systems, age‐specific hand‐to‐mouth behaviors, and disproportionately higher exposure relative to body mass [3]. Experimental studies further demonstrate that fine plastic particles, spanning the micro‐ and nanoscale, are capable of crossing gastrointestinal epithelial boundaries, accessing the bloodstream, and depositing within neural tissues, subsequently eliciting redox imbalance, glial activation, and conduct‐related deviations in experimental animal studies [4, 5]. Nevertheless, direct evidence linking internal microplastic burden to childhood neurodevelopmental disorders remains extremely scarce. In particular, the potential influence of early‐life microplastic contact on susceptibility to or clinical manifestation of attention‐deficit/hyperactivity disorder remains virtually uninvestigated.

Attention‐deficit/hyperactivity disorder (ADHD) is a prevalent neurodevelopmental disorder marked by enduring difficulties in sustaining focus, excessive motor activity, and diminished impulse regulation, all of which compromise scholastic achievement, interpersonal relationships, and general well‐being [6]. A recent meta‐analysis estimated that ADHD affects approximately 8% of children and adolescents worldwide, underscoring its substantial public health burden [7]. Although ADHD is highly heritable, twin and epidemiological studies converge on a gene‐by‐environment interaction model in which early‐life environmental insults, particularly those occurring during gestation and the perinatal period, modulate disease risk and symptom severity. Established environmental contributors include maternal smoking, prenatal nicotine exposure, and early‐life toxicant burden; however, the role of newly emerging environmental contaminants, notably early‐life microplastic exposure, remains poorly understood [8]. Oxidative stress and the inflammatory response represent key mechanistic convergence points between microplastic toxicity and ADHD pathophysiology [9]. Dysfunction of prefrontal cortical catecholamine signaling is widely recognized as a central neurobiological feature of ADHD. Dopamine and norepinephrine regulate attention, behavioral inhibition, working memory, motivation, and executive function, and disruption of these neurotransmitter systems is thought to underlie the core behavioral manifestations of the disorder [10]. Oxidative stress and inflammation may further impair catecholamine synthesis, receptor signaling, synaptic plasticity, and neuronal survival. Indeed, clinical studies have reported altered circulating cytokines, reduced antioxidant capacity, and elevated lipid‐peroxidation markers in children with ADHD compared with typically developing peers, although findings vary across populations and disease severity [11]. Notably, microplastics may affect the nervous system not only through direct tissue deposition but also via peripheral inflammation and oxidative stress [12]. In parallel, nanoplastic exposure has been shown to modify dopamine receptor expression in cultured cortical neurons, suggesting that plastic particles may interfere with dopaminergic signaling relevant to neurodevelopmental conditions [13]. We therefore hypothesized that early‐life microplastic exposure induces oxidative stress and inflammation that, in turn, disrupt dopamine homeostasis and exacerbate ADHD‐like behaviors.

In this study, we sought to determine whether early‐life exposure to polyethylene (PE) microplastics, the most abundantly detected polymer in the peripheral blood of children with ADHD in our pilot cohort, exacerbates ADHD‐like behaviors. To this end, we integrated a pilot pediatric cohort, multi‐omics analyses, computational approaches, and mechanistic animal experiments to investigate the relationship between circulating microplastic burden and ADHD. Our results showed that PE burden was positively associated with elevated inflammatory and oxidative stress markers, which in turn correlated with greater ADHD symptom severity. Through computational screening, we identified compounds capable of restoring PI3K/AKT1/Nrf2 signaling as both mechanistic validators and candidate intervention strategies against the neurotoxic effects of early‐life PE exposure. Specifically, we found that concurrent melatonin administration enhanced PI3K/AKT/Nrf2 signaling and downstream antioxidant defenses, thereby partially attenuating the behavioral, molecular, and metabolic abnormalities associated with PE co‐exposure. Furthermore, AAV‐mediated knockdown of PI3K in the prefrontal cortex markedly weakened the protective effects of melatonin, supporting PI3K/AKT1/Nrf2 signaling as a critical mechanistic mediator of its neuroprotective action. Together, our findings identify an association between circulating PE burden and ADHD‐related clinical and biological features in children and provide experimental evidence in mice that developmental PE co‐exposure can aggravate ADHD‐like phenotypes in a susceptible background.

## 2 Methods

### 2.1 Pilot Observational Study Design

This pilot cross‐sectional observational study was conducted at the Department of Pediatrics, Affiliated Hospital of Jiangnan University (Wuxi, China) between January 2025 and January 2026 based on our team's previous studies [14, 15, 16, 17]. A total of 90 children were consecutively enrolled, including 45 children with newly diagnosed attention‐deficit/hyperactivity disorder (ADHD) and 45 age‐ and sex‐matched healthy controls recruited from children undergoing routine physical examinations at the same hospital during the study period. The inclusion criteria for the ADHD group were as follows: (1) age 5–10 years; (2) a newly established diagnosis of ADHD according to the Diagnostic and Statistical Manual of Mental Disorders, Fifth Edition (DSM‐5) [18]; (3) no treatment with psychostimulants, atomoxetine, or other ADHD‐related medications within the 3 months preceding enrollment; (4) no known history of acute or unusually high exposure to industrial toxicants; and (5) no comorbid neurodevelopmental, psychiatric, neurological, or hereditary disorders that could substantially influence behavioral assessment or neurobiological measurements. Children with chronic systemic diseases, acute or recent infections, or use of medications or nutritional supplements known to substantially affect immune function, oxidative status, or lipid metabolism within the preceding 12 weeks were excluded. Healthy controls had no previous diagnosis or clinical evidence of ADHD or other major neurodevelopmental, neurological, or psychiatric disorders, and the same exclusion criteria were applied. To reduce potential confounding, children with ADHD and healthy controls were recruited from the same medical center during the same study period and were matched by age and sex. The exclusion of recent medication or nutritional supplement use, acute infection, chronic systemic disease, and unusually high occupational or industrial toxicant exposure was intended to reduce major clinical and treatment‐related sources of variability that could influence inflammatory, oxidative, or lipid‐metabolic measurements. However, habitual dietary intake, including consumption of omega‐3‐rich seafood, household plastic use, socioeconomic status, and indoor microplastic or PE‐containing dust exposure were not quantitatively assessed using validated exposure instruments and therefore could not be incorporated into covariate‐adjusted analyses [19, 20]. Demographic and baseline characteristics of the enrolled children are summarized in Table S1. Peripheral blood samples were collected and allocated for microplastic detection and characterization, inflammatory and oxidative stress measurements, targeted biochemical assays, metabolomic profiling, and transcriptomic analysis.

### 2.2 Clinical ADHD Severity Classification and Symptom Assessment

ADHD diagnosis and clinical severity were determined by qualified pediatric clinicians according to the Diagnostic and Statistical Manual of Mental Disorders, Fifth Edition (DSM‐5). Following comprehensive clinical assessment, children meeting the diagnostic criteria for ADHD were further classified as having mild, moderate, or severe ADHD according to the DSM‐5 severity specifier. Clinical severity was determined by considering both the number and intensity of symptoms beyond those required for diagnosis and the extent of associated functional impairment. Mild ADHD was characterized by few, if any, symptoms beyond those required for diagnosis and only minor functional impairment; severe ADHD was characterized by substantially more symptoms than required for diagnosis, several particularly severe symptoms, or marked functional impairment; and moderate ADHD represented an intermediate level of symptom burden or impairment between the mild and severe categories. Based on this clinician‐administered DSM‐5 assessment, the ADHD cohort was stratified into mild, moderate, and severe subgroups (n = 15 per subgroup). The Swanson, Nolan, and Pelham Questionnaire, Version IV, 18‐item form (SNAP‐IV‐18), and the Conners Third Edition Rating Scale (Conners‐3) were additionally administered to quantitatively characterize ADHD symptom burden [21, 22]. For the SNAP‐IV‐18, the combined average rating per item (ARI) was calculated by summing the scores of all 18 ADHD items and dividing the total by 18, with higher values indicating greater overall ADHD symptom burden. For the Conners‐3, raw scores were converted to age‐ and sex‐standardized T‐scores according to the corresponding normative scoring procedure, with higher T‐scores indicating greater symptom or behavioral concerns relative to the normative population. Importantly, the SNAP‐IV‐18 ARI and Conners‐3 T‐scores were not used as fixed numerical cutoffs for assigning participants to the mild, moderate, or severe clinical groups. Instead, both instruments were treated as complementary quantitative measures of symptom burden and were used to characterize and provide convergent support for the clinician‐defined DSM‐5 severity classification. Detailed scoring and interpretation procedures for the SNAP‐IV‐18 and Conners‐3 are provided in Questionnaires S1 and S2 and Tables S2 and S3.

### 2.3 Human Ethics Statement

The Ethics Committee of the Department of Pediatrics, Affiliated Hospital of Jiangnan University (Wuxi, China), granted approval for this study protocol (Approval ID: LS‐JN2025010416R2), and all procedures were conducted in accordance with the Declaration of Helsinki. Written informed consent was obtained from parents or legally authorized guardians before any child was enrolled. All clinical records and biological specimens were de‐identified prior to laboratory analysis.

### 2.4 Microplastic Detection Using Micro‐Raman Spectroscopy

Microplastic particles isolated from peripheral blood samples were identified using micro‐Raman spectroscopy, with sample digestion, purification, and filtration performed as detailed in Method S1 and our previous studies [23, 24]. To minimize observer‐dependent selection bias, all membrane filters were coded before analysis, and investigators responsible for particle screening and Raman identification were blinded to the clinical group. Each membrane was divided into predefined analytical fields and systematically scanned in a fixed sequence across the analyzable filter area under an integrated optical microscope. All particle‐like features meeting the predefined optical detection criteria were recorded and subjected to Raman spectroscopic analysis irrespective of their presumed polymer identity. Raman spectra were acquired from the central region of each candidate particle using a confocal micro‐Raman spectrometer. Instrument parameters, including laser wavelength, laser power, spectral acquisition range, integration time, and number of accumulations, were optimized to obtain adequate spectral intensity while minimizing thermal damage and were maintained consistently across all samples. Micro‐Raman measurements and polymer identification were performed using the Raman polymer spectral library of Scientific Compass (www.shiyanjia.com). For each candidate particle, a background spectrum was collected from an adjacent particle‐free region of the membrane to account for substrate‐derived signals. The acquired spectra were subjected to baseline correction and, where necessary, smoothing and cosmic‐ray removal before spectral matching. Polymer identity was assigned on the basis of characteristic Raman peaks, the overall spectral profile, and the corresponding library similarity score. All polymer assignments were independently reviewed by a second investigator who was also blinded to the clinical group, using both library matching and characteristic spectral features.

### 2.5 Quality Assurance and Quality Control

A comprehensive quality assurance and quality control procedure was implemented throughout sample preparation and micro‐Raman analysis based on our previous studies [23, 24]. Reagent blanks, filter blanks, and procedural blanks were included to assess potential contamination introduced by reagents, filtration materials, laboratory handling, or the analytical environment. Procedural blanks were processed in parallel with study samples and underwent the complete digestion, purification, filtration, and Raman‐identification workflow without addition of blood. Spectra obtained from blank filters and procedural controls were compared with those from study samples to identify and exclude background‐ or substrate‐derived signals. To evaluate analytical recovery, spike‐and‐recovery experiments were additionally performed using reference microplastic particles representative of the particle‐size range observed in the clinical samples. Reference PE, PP, and PS particles with nominal sizes of approximately 1 and 5 µm were added to blank matrix samples and processed through the complete analytical workflow. Recovery was calculated as: Recovery Rate (%) = Recovered particle counts/Spiked particle counts × 100%. The recovery efficiency, blank particle counts, and polymer‐specific background contamination results are summarized in Tables S4 and S5. All study samples were analyzed using identical sample‐processing, scanning, spectral‐acquisition, and polymer‐identification criteria to ensure consistency across analytical batches.

### 2.6 Chemicals and Reagents

Nicotine was purchased from Shanghai Maokang Biotechnology Co., Ltd. (Shanghai, China; cat. no. MS2005‐1G). Melatonin was obtained from MedChemExpress (MCE, Monmouth Junction, NJ, USA; cat. no. HY‐B0075). The primary antibodies used for western blotting were as follows: phospho‐PI3 kinase p85 (Tyr458) (cat. no. 17366S), PI3 kinase p85 (cat. no. 4257S), PI3 Kinase p110 alpha (cat. no. 4249S), phospho‐AKT1 (Ser473) (cat. no. 9018S), AKT1 (cat. no. 2938S), phospho‐GSK‐3β (Ser9) (cat. no. 5558S), and GSK‐3β (cat. no. 12456S), all purchased from Cell Signaling Technology (Danvers, MA, USA). Antibodies against NFE2L2/Nrf2 (cat. no. 16396‐1‐AP), Keap1 (cat. no. 10503‐2‐AP), tyrosine hydroxylase (TH; cat. no. 25859‐1‐AP), dopamine receptor D1 (DRD1; cat. no. 17934‐1‐AP), and β‐actin (cat. no. 66009‐1‐Ig) were obtained from Proteintech (Wuhan, China).

### 2.7 Measurement of Circulating Cytokines and Oxidative Stress Markers

Quantification of circulating cytokines and oxidative stress indicators was carried out with commercially procured assay kits, following the protocols furnished by the suppliers and adhering to methodologies reported in our earlier investigations [25]. Serum concentrations of interleukin‐6 (IL‐6), interleukin‐1β (IL‐1β), and tumor necrosis factor‐α (TNF‐α) in human specimens were assessed via enzyme‐linked immunosorbent assay (ELISA) using the following reagents: IL‐6 (catalogue number E‐EL‐H6156), IL‐1β (E‐EL‐H0149), and TNF‐α (E‐EL‐H0109), all sourced from Elabscience Biotechnology Co., Ltd. (Wuhan, China). For murine samples, levels of the aforementioned cytokines were evaluated with species‐specific ELISA reagents obtained from Elabscience Biotechnology Co., Ltd.: IL‐6 (E‐EL‐M0044), IL‐1β (E‐EL‐M0037), and TNF‐α (E‐EL‐M3063). Concentrations of malondialdehyde (MDA) and reduced glutathione (GSH) across human and murine specimens were determined with commercially supplied detection kits (MDA: E‐EL‐0060; GSH: E‐EL‐0026; Elabscience Biotechnology Co., Ltd.). Superoxide dismutase (SOD) enzymatic activity was measured with a dedicated activity assay kit (BC0170; Beijing Solarbio Science & Technology Co., Ltd., Beijing, China). Every analytical procedure was executed strictly in line with the supplier's recommended protocols, and all specimens were processed under uniform experimental settings to reduce variation between assays.

### 2.8 Quantification of Polyunsaturated Fatty Acids and Lipid Peroxidation

Circulating polyunsaturated fatty acids and lipid peroxidation markers were evaluated via commercially procured detection reagents based on our previous studies [16, 24], with procedures executed strictly as per the supplier's guidelines. Arachidonic acid (AA) was assessed with an Arachidonic Acid ELISA kit (catalogue number E‐EL‐0051; Elabscience Biotechnology Co., Ltd., Wuhan, China). Phosphatidylcholine hydroperoxide (PCOOH) was quantified employing a commercial detection kit (catalogue number E‐BC‐K796‐M; Elabscience Biotechnology Co., Ltd., Wuhan, China). Eicosapentaenoic acid (EPA), docosahexaenoic acid (DHA), and 8‐isoprostaglandin F2α (8‐iso‐PGF2α) were measured via enzyme‐linked immunosorbent assay (ELISA) using commercially sourced kits (EPA, catalogue number ELK9817; DHA, catalogue number ELK8137; 8‐iso‐PGF2α, catalogue number ELK0151; ELK Biotechnology Co., Ltd., Wuhan, China). Each analytical run was conducted strictly following the vendor's specifications, with all specimens processed in a standardized manner to reduce batch‐to‐batch discrepancy. The AA/EPA and AA/DHA ratios were subsequently calculated from the corresponding measured concentrations to assess the relative balance between arachidonic acid and omega‐3 polyunsaturated fatty acids.

### 2.9 Untargeted Metabolomic Profiling and Metabolite Identification

To characterize metabolic alterations associated with ADHD pathophysiology, untargeted metabolomic profiling was performed in age‐ and sex‐matched children with severe ADHD and healthy controls. Fasting venous blood specimens were drawn from peripheral vessels between 7:00 and 9:00 AM to reduce possible confounding from circadian rhythms and dietary intake based on our previous studies [14, 24]. Within 2 h of collection, serum was isolated by centrifuging at 3 000 × g for 10 min at 4°C, divided into aliquots to prevent multiple freeze‐thaw events, and preserved at −80°C pending further analysis. Serum samples were subsequently transferred on dry ice to OE Biotech Co., Ltd. (Shanghai, China) for untargeted metabolomic profiling according to standardized procedures and our previously established methodology. Differential metabolites were identified using a combined multivariate and univariate statistical strategy. First, orthogonal partial least squares discriminant analysis (OPLS‐DA) was conducted to assess discrepancies in overall metabolic patterns distinguishing children with severe ADHD from healthy counterparts. Metabolites exhibiting a variable importance in projection (VIP) score exceeding 1.0 were regarded as making a meaningful contribution to distinguishing between groups. Model robustness and the risk of overfitting were evaluated through a permutation test involving 200 iterations. Second, individual metabolites were compared between groups via an unpaired two‐tailed Student's t‐test. Derived *p*‐values underwent correction for multiple testing with the false discovery rate approach, whereby a *q*‐value below 0.05 was taken as indicating statistical significance. Third, a fold‐change criterion was applied to ensure biological relevance. Metabolites were defined as differentially abundant only when concurrently meeting the following thresholds: VIP greater than 1.0, *q*‐value less than 0.05, and absolute log_2_ fold change exceeding 1. The identified differential metabolites were then analyzed for pathway enrichment against the Kyoto Encyclopedia of Genes and Genomes (KEGG) database via the OECloud platform (OE Biotech, Shanghai, China). Enriched metabolic pathways were ranked according to statistical significance and metabolite representation. Particular attention was subsequently given to pathways related to glycerophospholipid metabolism, glutathione metabolism, polyunsaturated fatty acid metabolism, and oxidative stress based on the unbiased enrichment results. Correlation analyses between selected metabolites and clinical or biochemical parameters were also performed using the OECloud platform where appropriate.

### 2.10 Peripheral Blood RNA Sequencing and Transcriptomic Analysis

To investigate gene expression alterations associated with ADHD pathophysiology, peripheral blood transcriptomic profiling was performed in age‐ and sex‐matched children with severe ADHD and healthy controls. Venous blood specimens were drawn in their entirety with PAXgene Blood RNA Tubes (Qiagen, Germany), which facilitate prompt stabilization of intracellular RNA and curtail ex vivo transcriptional alterations, in line with our prior work [26]. In brief, 2.5–3.0 mL of fasting venous blood was aspirated straight into PAXgene collection vessels and carefully inverted eight to ten times to achieve thorough homogenization, as directed by the supplier's guidelines. Specimens were then conveyed under uniform transport conditions to GENEWIZ (Suzhou, China) for total RNA extraction, RNA quality assessment, library preparation, and high‐throughput sequencing. Following sequencing and quality control, transcript abundance was quantified and normalized for downstream analyses. Principal component analysis (PCA) was performed to evaluate the overall distribution of transcriptomic profiles between children with severe ADHD and healthy controls. Differential expression analysis was performed between the two groups, and *p*‐values were corrected for multiple testing using the Benjamini–Hochberg (BH) procedure. Differentially expressed genes (DEGs) were defined as those satisfying both BH‐adjusted *p* < 0.05 and |log2 fold change| ≥ 0.58 [27]. Gene Ontology (GO) and Kyoto Encyclopedia of Genes and Genomes (KEGG) enrichment analyses were subsequently performed using the FDR‐significant DEGs, with BH‐adjusted *p* < 0.05 considered statistically significant. Gene Set Enrichment Analysis (GSEA) was additionally performed using ranked gene‐expression profiles and gene sets obtained from the Molecular Signatures Database, with FDR‐adjusted significance used for pathway interpretation.

### 2.11 Animal Design and Drug Treatment

A perinatal nicotine exposure (PNE) paradigm was implemented to elicit ADHD‐like behavioral phenotypes in the offspring of treated dams, following the methodology reported in an earlier study [28]. Adult C57BL/6J mice were maintained in conventional animal housing facilities under a 12 h alternating light and dark photoperiod, with unrestricted availability of standard chow and drinking water. Female and male mice were mated at a ratio of 2:1. Detection of a vaginal plug served as confirmation of conception, with the day of observation recorded as embryonic day 0.5 (E0.5). Following confirmation, gravid females were allocated to treatment cohorts through a randomization procedure. Beginning on E1.5, dams assigned to the PNE groups received nicotine at 100 µg/mL in drinking water throughout pregnancy and lactation until postnatal day 21 (P21), whereas control dams received regular drinking water [29, 30]. Nicotine‐containing water was freshly prepared and replaced regularly. This exposure window was selected to encompass prenatal and early postnatal neurodevelopment, two critical periods of brain susceptibility to environmental insults. Polyethylene (PE) microplastics with a particle size of approximately 5 µm were used for early‐life exposure. This size was selected because it approximated the upper range of PE particles detected in the pediatric cohort and enabled evaluation of oxidative stress, inflammatory activation, and neurobehavioral alterations induced by relatively small particles, as supported by recent studies [31]. The low and high PE doses (1 and 3 mg/kg) were selected based on previous studies employing low‐dose, environmentally relevant microplastic exposure paradigms [32, 33], and were intended to model sustained developmental exposure rather than acute high‐dose toxicity. PE suspensions were freshly prepared before administration and adequately dispersed to minimize particle aggregation. PE and melatonin were administered to pregnant and subsequently lactating dams once daily by oral gavage from E1.5 to P21, with doses adjusted according to maternal body weight. The five experimental groups were treated as follows: Control, regular drinking water plus vehicle gavage; ADHD, nicotine‐containing drinking water (100 µg/mL) plus vehicle gavage; ADHD + Low PE, nicotine water plus PE at 1 mg/kg/day; ADHD + High PE, nicotine water plus PE at 3 mg/kg/day; and ADHD + High PE + Melatonin, nicotine water plus PE at 3 mg/kg/day and melatonin at 30 mg/kg/day. Equivalent volumes of vehicle were administered to the corresponding control groups to minimize handling‐related effects. Melatonin was co‐administered with nicotine and PE throughout E1.5–P21. Accordingly, this experimental arm was designed to evaluate the protective effects of melatonin during developmental exposure rather than its therapeutic efficacy against an established ADHD‐like phenotype. In a separate cohort, the effect of PE exposure alone was examined without nicotine co‐exposure. Dams were randomly assigned to three groups (n = 6 per group): Control (regular drinking water plus vehicle gavage), Low PE (PE at 1 mg/kg/day), and High PE (PE at 3 mg/kg/day), with PE administered by oral gavage from E1.5 to P21 as described above. Male offspring (n = 6 per group) in the PE‐only cohort were subjected to the three‐chamber social interaction test and 5‐CSRTT. Social behavior, attentional performance, premature responding, and perseverative responding were analyzed to determine whether PE exposure alone was sufficient to induce detectable ADHD‐relevant behavioral abnormalities.

To minimize variability associated with sex‐dependent differences in neurodevelopmental and behavioral outcomes, only male offspring were included. After weaning at P21, male offspring were randomly selected from each group (n = 8 per group) for behavioral testing commencing at P28 and downstream analyses. Body weight was monitored throughout early postnatal development. Behavioral assessments were conducted using a predefined test battery encompassing complementary neurobehavioral domains. These included the three‐chamber social interaction test, open‐field test, marble‐burying test, novel object recognition test, Morris water maze, and the five‐choice serial reaction time task (5‐CSRTT). The 5‐CSRTT was specifically incorporated to evaluate ADHD‐relevant attentional performance and impulsive responding, whereas the remaining behavioral paradigms assessed social behavior, anxiety‐related and repetitive behavior, recognition memory, and spatial learning and memory. Investigators performing behavioral testing and analysis were blinded to group allocation whenever feasible [34, 35]. After completion of behavioral experiments, mice were euthanized, and peripheral blood, prefrontal cortex, and hippocampal tissues were collected for biochemical, histological, transcriptomic, and molecular analyses. All animal procedures were approved by the Institutional Animal Care and Use Committee of the Affiliated Hospital of Jiangnan University (approval No. JN.No20251114C017150[205]).

### 2.12 Statistical Analysis

All statistical analyses were performed using GraphPad Prism and the R programming environment. Continuous variables are presented as mean ± standard deviation (SD). The Shapiro–Wilk test was used to assess normality. For comparisons between two independent groups, normally distributed data were analyzed with an unpaired two‐tailed Student's *t*‐test, and non‐normally distributed data with the Mann–Whitney *U*‐test. For comparisons among more than two groups, one‐way analysis of variance (ANOVA) followed by appropriate post hoc multiple‐comparison tests was used for normally distributed data, whereas the Kruskal‐Wallis test followed by Dunn's post hoc test was used otherwise. Repeated‐measures two‐way ANOVA was applied to longitudinal endpoints, including body‐weight trajectories and Morris water maze training performance, as well as to 5‐CSRTT outcomes across stimulus durations or inter‐trial intervals, with group as the between‐subject factor and stimulus duration or inter‐trial interval as the within‐subject factor, followed by appropriate post hoc multiple‐comparison procedures. Associations between continuous variables were assessed using Spearman's rank correlation. A two‐tailed *p*‐value < 0.05 was considered statistically significant. To enhance transparency and reproducibility, the custom analysis scripts in R are available on GitHub (https://github.com/PediatricLab‐Jiangnan/Earlylife‐ADHD‐PE). Moreover, the raw datasets generated in this study have been deposited in the Genome Sequence Archive (GSA) under the following accession numbers: human serum metabolomics (OMIX013618) and human blood RNA—seq (HRA015358). Additional methods used in this study are described in Methods S1–S17.

## 3 Results

### 3.1 Increased PE Burden Was Associated With ADHD Symptom Severity, Inflammation, and Impaired Antioxidant Capacity

To explore the potential link between microplastic exposure and ADHD in children, we established a pilot observational cohort comprising 45 children with ADHD and 45 healthy controls. Circulating microplastics were identified using micro‐Raman spectroscopy following standard protocols detailed in our previous studies (Figure 1A,B) [23]. Raman spectra confirmed the presence of polyethylene (PE), polypropylene (PP), and polystyrene (PS) particles in blood samples (Figure 1C), and additional polymer types, including polyethylene terephthalate (PET), polyvinyl chloride (PVC), polymethyl methacrylate (PMMA), polyurethane (PU), and polybutylene adipate terephthalate (PBAT), were detected in both groups (Figure 2A and Figure S1). Detection rates of all major polymer types were numerically higher in children with ADHD than in controls (Figure 2B). PET showed the highest detection rate in peripheral blood (82.2% in controls and 95.6% in the ADHD group); however, analysis of relative polymer composition revealed that PE was the most abundant polymer in children with ADHD, accounting for 36.7% of total microplastic abundance vs. 27.2% in controls (Figure 2C). Both the relative abundance of PE (Figure 2D) and blood PE concentrations (Figure 2E) were significantly higher in the ADHD group than in controls (*p* < 0.05), whereas mean PE particle size did not differ between groups (1.72 µm vs. 1.96 µm; maximal values nearly 5 µm; Figure 2F). We therefore focused on PE, the most abundant polymer type in children with ADHD, to examine the association between PE burden and ADHD symptom severity.

**FIGURE 1:**
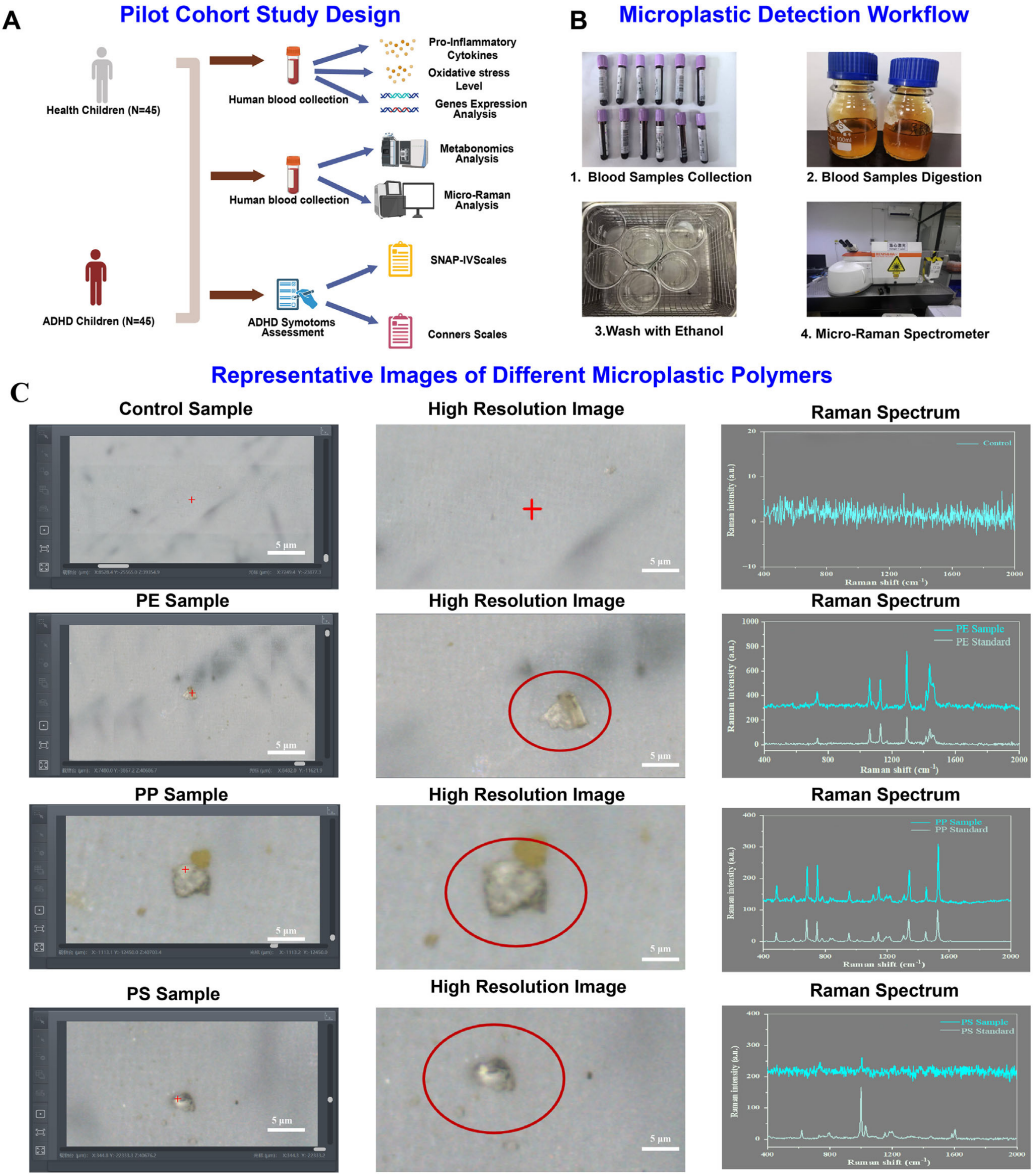
Pilot observational cohort design, blood microplastic detection workflow, and representative Raman characterization of microplastic polymers. (A) Schematic overview of the pilot cohort study. (B) Workflow for the detection and identification of microplastics in blood samples, comprising blood collection, sample digestion, ethanol washing, and micro‐Raman spectroscopic analysis. (C) Representative microscopic images, enlarged high‐resolution images, and corresponding Raman spectra of a control sample and particles identified as polyethylene (PE), polypropylene (PP), and polystyrene (PS) (Scale bar = 5 µm).

**FIGURE 2:**
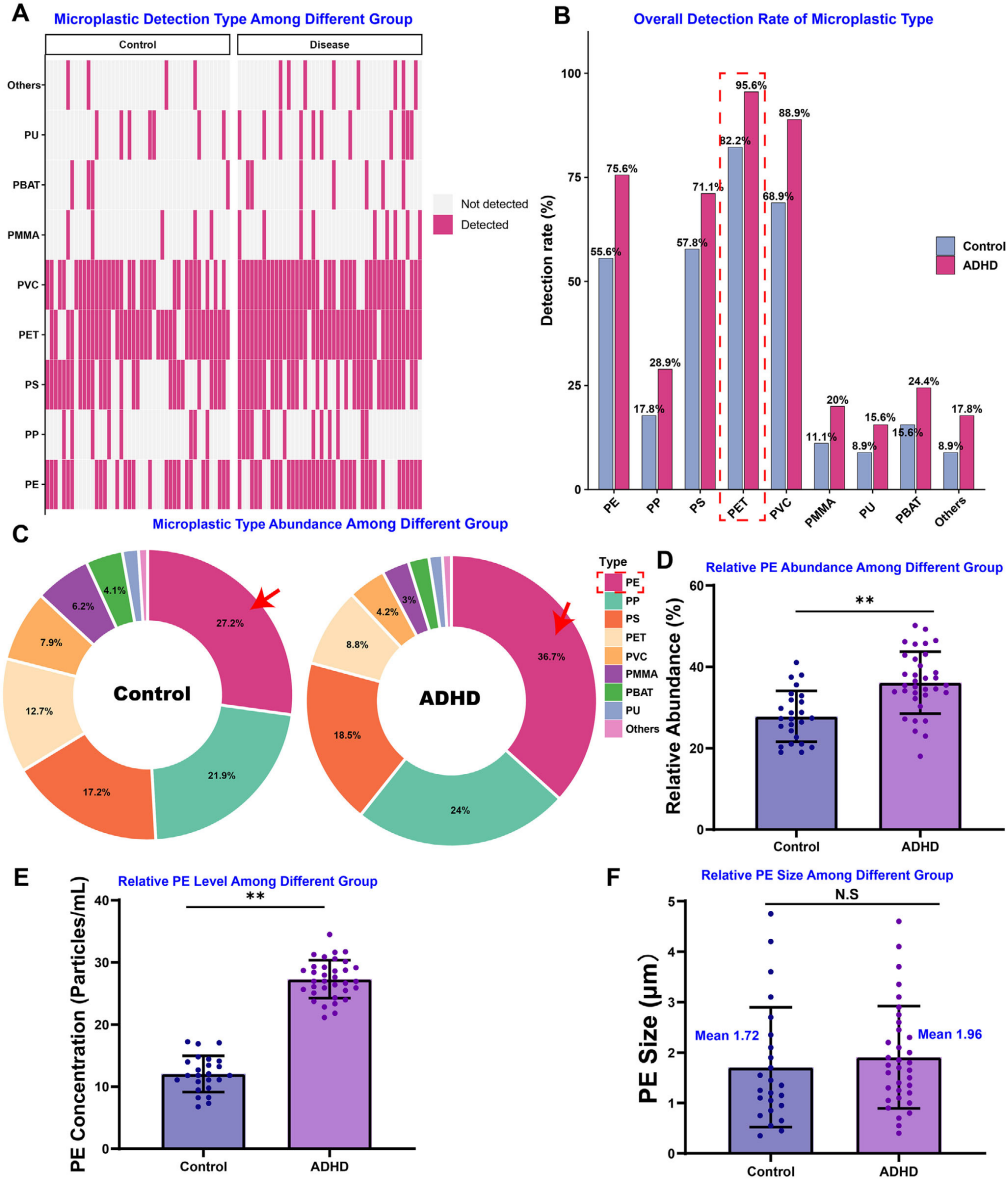
Distribution and quantitative characteristics of circulating microplastics in children with ADHD and healthy controls. (A) Detection map of microplastic polymer types in individual blood samples from healthy control children (Control) and children with ADHD (Disease). Each column represents one participant; magenta bars indicate polymers detected in that individual, and light gray indicates not detected. (B) Overall detection rates of each microplastic polymer type in the Control and ADHD groups (n = 45 per group). (C) Relative composition of the detected microplastic polymers in the control and ADHD groups. Polyethylene (PE) accounted for 27.2% of the total microplastic abundance in controls and 36.7% in children with ADHD, as indicated by the red arrows. (D) Relative abundance of PE in PE‐positive blood samples from the control and ADHD groups. (E) PE microplastic concentration in blood samples of the Control and ADHD groups. (F) Comparison of PE particle size between the two groups. ^*^ *p* < 0.05; ^**^ *p* < 0.01; N.S., not significant.

We next measured circulating cytokines and redox‐related biomarkers. Blood IL‐6 levels were significantly elevated in children with ADHD (*p* < 0.01; Figure 3A), whereas IL‐1β and TNF‐α levels did not differ between groups (Figure 3B,C). Children with ADHD also exhibited increased malondialdehyde (MDA) and decreased glutathione (GSH) levels (*p* < 0.05; Figure 3D,E), while superoxide dismutase (SOD) activity was numerically lower but not statistically significant (Figure 3F). Children with ADHD were stratified into mild, moderate, and severe subgroups according to clinician‐administered DSM‐5 severity specifiers; SNAP‐IV and Conners‐3 scores were used to quantitatively characterize symptom burden (Figure 3G,H) [36, 37]. The relative contribution of PE to the total circulating microplastic burden increased with symptom severity, accounting for approximately 28.2%, 36.0%, and 44.6% of detected polymers in the mild, moderate, and severe subgroups, respectively (Figure 3I). Consistently, blood PE concentrations were significantly higher in the moderate than in the mild subgroup and were highest in children with severe ADHD (*p* < 0.01; Figure 3J). Spearman correlation analysis demonstrated positive associations between blood PE concentration and both SNAP‐IV scores (R = 0.58, *p* < 0.001; Figure 3K) and Conners T‐scores (R = 0.56, *p* < 0.001; Figure 3L), indicating that greater PE burden was associated with more severe ADHD symptoms.

**FIGURE 3:**
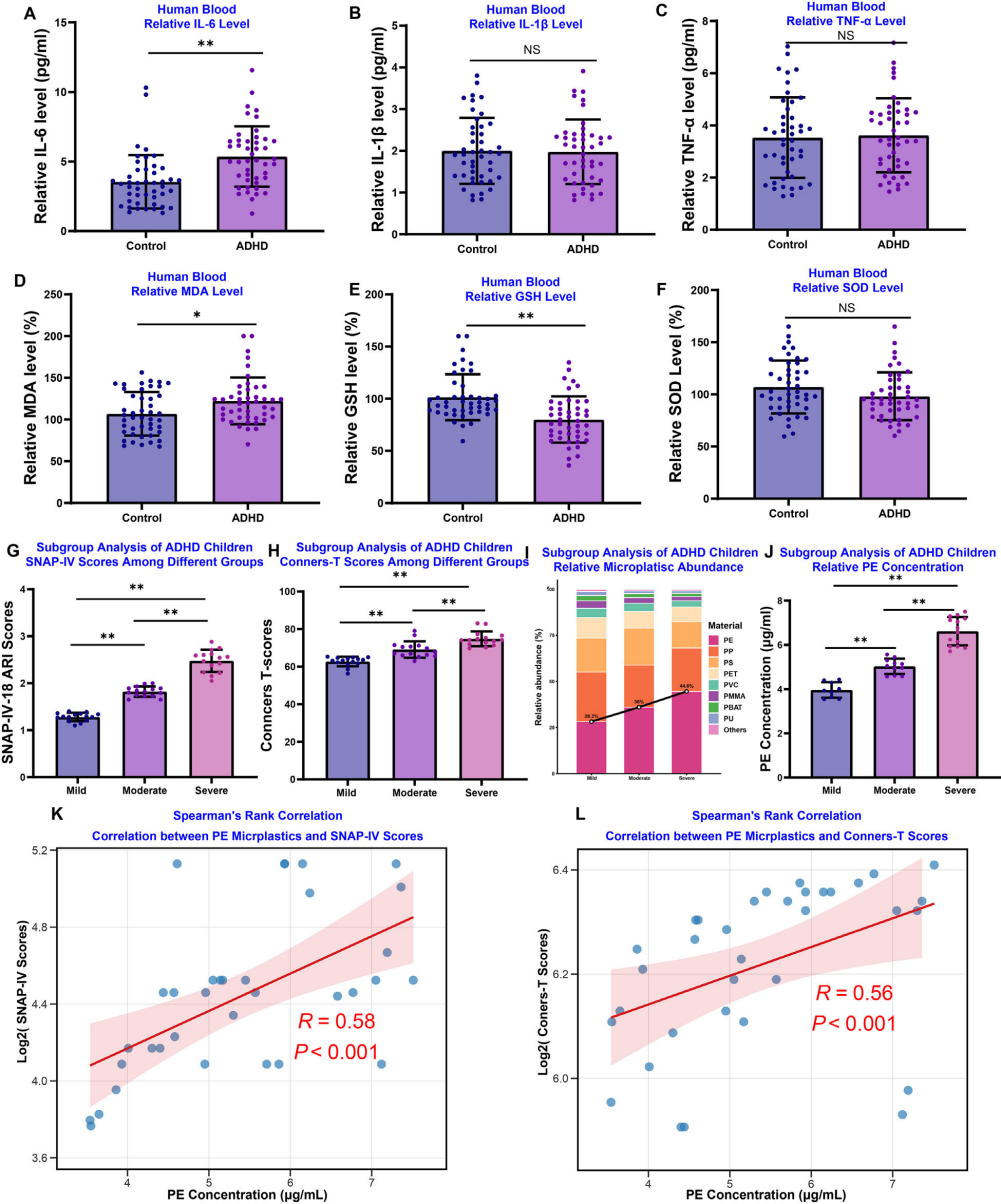
Peripheral inflammation and oxidative stress in children with ADHD, and the association between blood PE microplastic burden and ADHD symptom severity. (A–C) Peripheral blood levels of the pro‐inflammatory cytokines IL‐6, IL‐1β, and TNF‐α, respectively, in healthy control children and children with ADHD. IL‐6 levels were significantly elevated in the ADHD group, whereas no significant between‐group differences were observed for IL‐1β or TNF‐α. (D–F) Relative peripheral blood levels of the oxidative stress markers malondialdehyde (MDA), reduced GSH, and SOD, respectively. Children with ADHD exhibited increased MDA levels and decreased GSH levels, whereas SOD levels did not differ significantly between the groups. (G,H) SNAP‐IV‐18 combined ARI scores (G) and Conners‐3 T‐scores (H) across clinician‐defined mild, moderate, and severe ADHD subgroups based on DSM‐5 severity assessment. Both questionnaire measures showed progressively greater symptom burden across increasing clinical severity. (I) Relative composition of different microplastic polymer types across the mild, moderate, and severe ADHD subgroups. The relative abundance of polyethylene (PE) increased progressively with ADHD symptom severity. (J) Blood PE microplastic concentration across ADHD severity subgroups. (K,L) Spearman's rank correlation analyses between blood PE concentration and SNAP‐IV scores (K) and Conners‐T scores (L) in children with ADHD. ^*^ *p* < 0.05; ^**^ *p* < 0.01; N.S., not significant.

Subgroup analyses further showed that circulating IL‐6 levels were markedly elevated in children with severe ADHD compared with the mild subgroup, but did not differ significantly from the moderate subgroup (*p* < 0.01; Figure 4A). MDA levels showed a modest increasing trend without reaching significance among severity subgroups (Figure 4B), whereas GSH levels declined progressively and were significantly lower in the moderate and severe subgroups than in the mild subgroup (*p* < 0.01; Figure 4C). Blood PE concentration was positively correlated with IL‐6 levels (R = 0.61, *p* < 0.001; Figure 4D) and negatively correlated with GSH levels (R = −0.67, *p* < 0.001; Figure 4F), whereas its association with MDA did not reach statistical significance (R = 0.3, p = 0.085; Figure 4E). IL‐1β, TNF‐α, and SOD did not differ significantly among the ADHD severity subgroups (Figure S2A–C). Collectively, these findings indicate that increased PE burden is associated with ADHD symptom severity, inflammation, and impaired antioxidant capacity.

**FIGURE 4:**
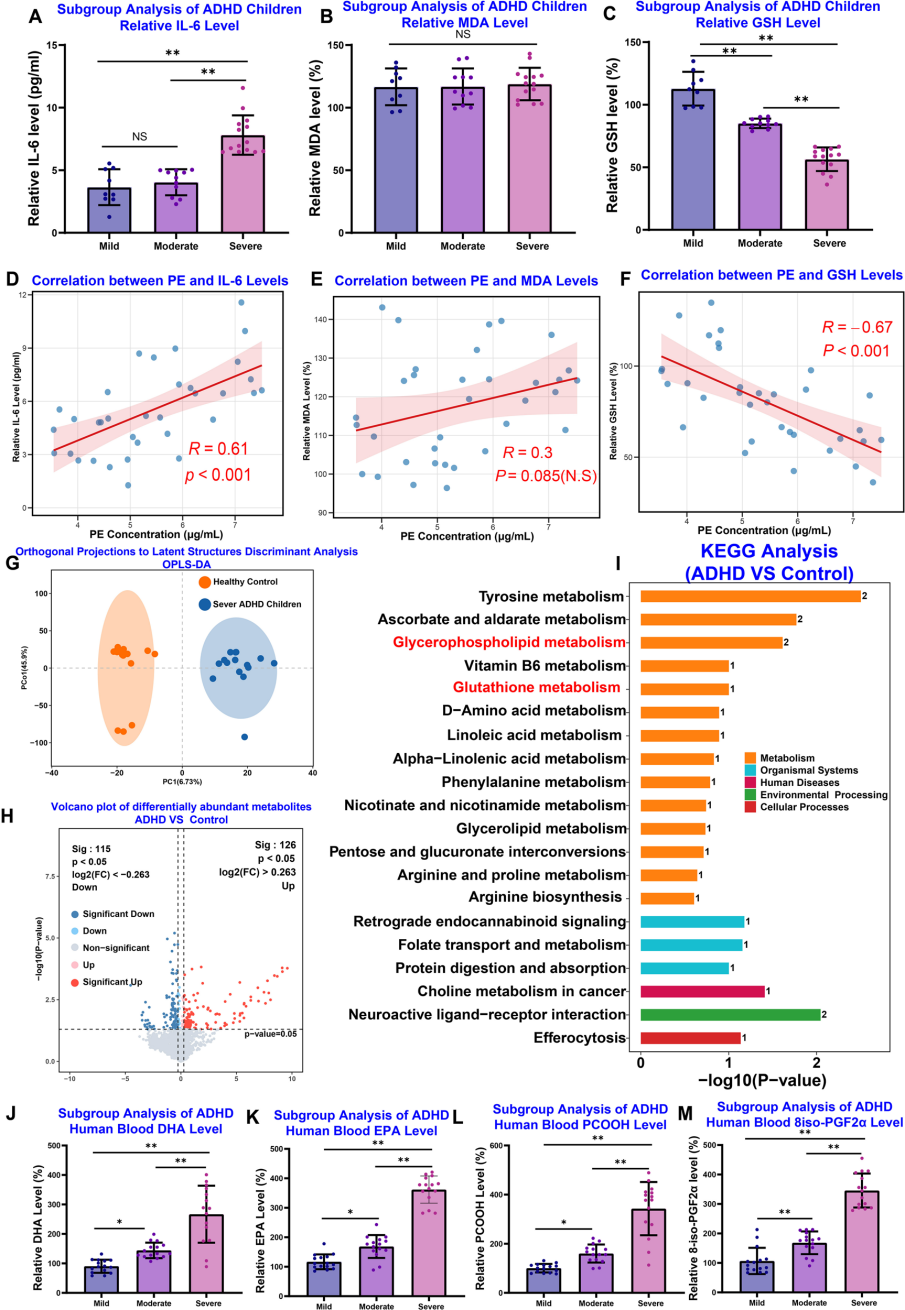
Associations of polyethylene microplastic exposure with inflammation, oxidative stress, and metabolic alterations across ADHD severity subgroups. (A–C) Subgroup analyses of peripheral blood IL‐6, MDA, and reduced GSHl levels, respectively, in children with mild, moderate, or severe ADHD. IL‐6 levels were significantly elevated in the severe ADHD subgroup, whereas MDA levels did not differ significantly among the three subgroups. GSH levels progressively decreased with increasing ADHD symptom severity. (D–F) Correlation analyses between blood PE concentration and IL‐6, MDA, and GSH levels, respectively. PE concentration was positively correlated with IL‐6 levels, was not significantly correlated with MDA levels, and was negatively correlated with GSH levels. (G) OPLS‐DA score plot showing the separation of blood metabolomic profiles between healthy controls and children with severe ADHD. (H) Volcano plot of differentially abundant metabolites between children with ADHD and healthy controls. (I) Kyoto Encyclopedia of Genes and Genomes (KEGG) enrichment analysis of differentially abundant metabolites between the ADHD and control groups. Pathways highlighted in red (glycerophospholipid metabolism and glutathione metabolism) are closely associated with lipid peroxidation and oxidative stress. (J–M) Relative blood levels of DHA (J), EPA (K), phosphatidylcholine hydroperoxide (PCOOH, L), and 8‐iso‐prostaglandin F2α (8‐iso‐PGF2α, M) across ADHD severity subgroups. ^*^ *p* < 0.05, ^**^ *p* < 0.01; NS, not significant.

### 3.2 Multi‐Omics Analyses Implicate PI3K/AKT1/Nrf2 Signaling in the Association Between PE Burden and ADHD‐Related Phenotypes

To explore metabolic pathways that may link PE exposure to ADHD‐related phenotypes, we performed untargeted metabolomic profiling of peripheral blood from age‐ and sex‐matched children with severe ADHD and healthy controls. Orthogonal partial least squares discriminant analysis revealed clear separation between the two groups (Figure 4G), and differential analysis identified 126 increased and 115 decreased metabolites in children with severe ADHD (Figure 4H). Among the top 20 enriched KEGG pathways, glycerophospholipid metabolism and glutathione metabolism were particularly relevant to the observed abnormalities in oxidative stress and lipid homeostasis (Figure 4I) [24]. We next quantified circulating polyunsaturated fatty acids and lipid peroxidation products across the mild, moderate, and severe subgroups. Docosahexaenoic acid (DHA) and eicosapentaenoic acid (EPA) levels progressively decreased with increasing severity, whereas arachidonic acid (AA) remained largely unchanged; consequently, both the AA/DHA and AA/EPA ratios increased progressively from mild to severe ADHD (Figure 4J,K and Figure S2D–F). In parallel, circulating phosphatidylcholine hydroperoxide and 8‐isoprostaglandin F2α levels increased with symptom severity, peaking in children with severe ADHD (Figure 4L,M). These findings indicate that increasing ADHD severity is accompanied by disrupted glycerophospholipid homeostasis and enhanced lipid peroxidation, which may further amplify oxidative stress and inflammation.

To identify molecular targets linking PE exposure to ADHD, ADHD‐related genes from GeneCards were intersected with PE‐related genes retrieved from the Comparative Toxicogenomics Database, yielding 32 shared candidate genes (Figure 5A and Method S1). Protein–protein interaction analysis revealed extensive connectivity among these targets, with AKT1, IL6, PIK3CA, and NFE2L2 occupying central network positions (Figure 5B) [16]. Reactome enrichment analysis implicated signaling by interleukins, nuclear events mediated by NFE2L2, and CD28‐dependent PI3K/AKT signaling (Figure 5C), and the maximal clique centrality, edge percolated component, and degree algorithms jointly identified 14 hub genes (Figure 5D). Mendelian randomization analyses were then performed to evaluate associations between genetically predicted expression of the 14 hub genes and ADHD risk. Among the evaluated genes, genetically predicted AKT1 expression showed the strongest nominal inverse association with ADHD risk (P < 0.05); however, this association did not remain statistically significant after correction for multiple testing (FDR >0.05; Figure 5E and Method S2 and Table S6). The direction of association was broadly consistent across sensitivity estimators (Figure 5F). These findings prioritized AKT1 as a candidate gene associated with ADHD susceptibility within the PE‐related molecular network.

**FIGURE 5:**
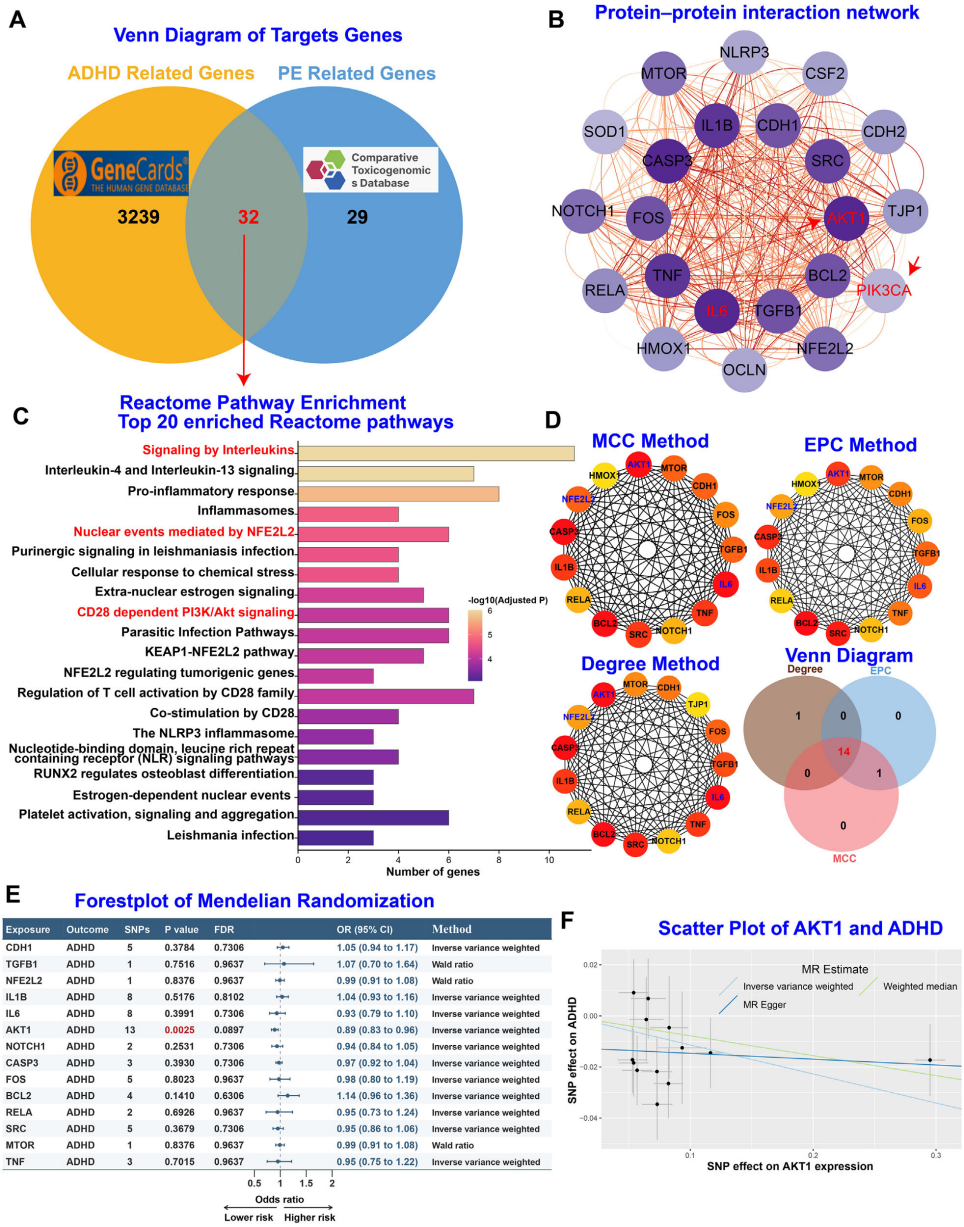
Network toxicology and Mendelian randomization prioritize AKT1 as a candidate gene within the PE–ADHD molecular network. (A) Venn diagram showing the overlap between ADHD‐related genes retrieved from the GeneCards database and polyethylene (PE)‐related genes obtained from the Comparative Toxicogenomics Database (CTD), identifying 32 shared targets. (B) Protein–protein interaction (PPI) network of the 32 overlapping genes constructed using the STRING database. Node size and color intensity reflect the degree of connectivity. Red arrows indicate AKT1 and PIK3CA, two key components of the PI3K/AKT signaling pathway. (C) Reactome pathway enrichment analysis of the overlapping targets, showing the top 20 enriched pathways. Pathways highlighted in red, including signaling by interleukins, nuclear events mediated by NFE2L2, and CD28‐dependent PI3K/AKT signaling, are closely related to inflammation, oxidative stress responses, and PI3K/AKT pathway regulation. (D) Identification of hub genes using three cytoHubba algorithms, including maximal clique centrality (MCC), edge percolated component (EPC), and degree. Fourteen overlapping hub genes were identified by Venn analysis. (E) Forest plot of two‐sample Mendelian randomization analyses evaluating the associations between genetically predicted expression of the 14 hub genes and ADHD risk. Estimates are shown as odds ratios (ORs) with 95% confidence intervals, together with nominal *p*‐values and false discovery rate (FDR)‐adjusted *p*‐values. Among the evaluated genes, AKT1 showed a nominal inverse association with ADHD risk using the inverse‐variance weighted method. (F) Scatter plot of the Mendelian randomization analysis for AKT1 and ADHD, showing the effects of instrumental SNPs on AKT1 expression (*x*‐axis) and ADHD risk (*y*‐axis), together with the fitted lines from the different MR methods.

To further investigate the involvement of PI3K/AKT1/Nrf2 signaling, peripheral blood transcriptomic profiling was performed in age‐ and sex‐matched children with severe ADHD and healthy controls. PCA showed a group‐dependent shift in global gene‐expression profiles (Figure 6A). After correction for multiple testing using the Benjamini–Hochberg procedure, 339 genes met the predefined differential‐expression criteria of BH‐adjusted *p* < 0.05 and |log2FC| ≥ 0.58, including 201 upregulated and 138 downregulated genes in children with severe ADHD (Figure 6B). GO enrichment analysis of the FDR‐significant DEGs highlighted regulation of leukocyte activation and phosphatidylinositol phosphorylation (Figure 6C), whereas KEGG analysis identified cytokine–cytokine receptor interaction and PI3K‐Akt signaling among the significantly enriched pathways (Figure 6D). GSEA further demonstrated enrichment of the IL‐6 production‐related gene set in severe ADHD (NES = 2.19, FDR = 0.004; Figure 6E). Expression analysis of predefined PI3K/AKT1/Nrf2 pathway‐related genes showed reduced PIK3CA, PIK3R1, AKT1, NFE2L2, and GCLC expression in severe ADHD (Figure 6F). RT‐qPCR analysis across the clinician‐defined ADHD severity subgroups further showed that reductions in these genes were most evident in the severe subgroup (Figure 6G). Immune‐cell enrichment analysis additionally revealed altered signatures of CD8 T cells, regulatory T cells, γδ T cells, and resting and activated mast cells, supporting peripheral immune dysregulation (Figure S3 and Method S4) [15].To further evaluate the robustness of the broader transcriptomic alterations in an independent dataset, we additionally analyzed the publicly available whole‐blood dataset GSE159104, comprising 39 children with ADHD and 37 healthy controls. Using BH‐adjusted *p* < 0.05 together with fold change >1.25 or <0.80, 161 DEGs were identified, including 129 upregulated and 32 downregulated genes (Figure S4A,B). Functional enrichment analysis highlighted peroxisome‐related terms together with pathways involving ribosomal and proteostatic functions (Figure S4C,D). The peroxisome‐related enrichment provides complementary transcriptomic evidence of disturbed cellular redox and metabolic homeostasis in ADHD, which may intersect with redox‐sensitive PI3K/AKT and Nrf2 signaling [38]. Together, the metabolomic, network, genetic, transcriptomic, and immune profiling results converge on PI3K/AKT1/Nrf2 signaling as a candidate molecular pathway linking PE exposure to lipid peroxidation, oxidative stress, peripheral inflammation, and ADHD symptom severity.

**FIGURE 6:**
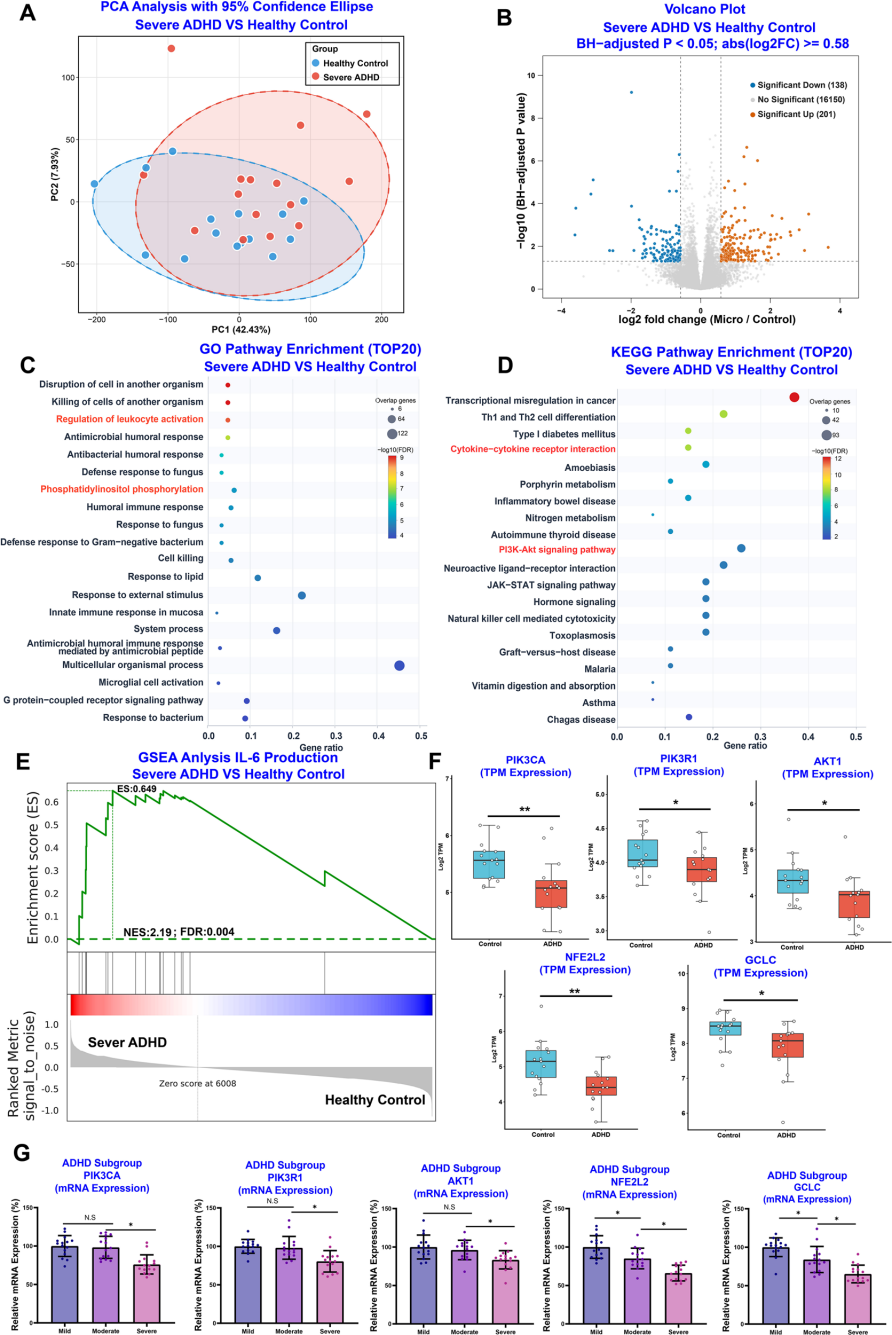
Transcriptomic profiling identifies inflammatory alterations and reduced PI3K/AKT1/Nrf2 pathway‐related gene expression in children with severe ADHD. (A) Principal component analysis (PCA) of peripheral blood transcriptomic profiles from healthy controls and children with severe ADHD, with 95% confidence ellipses. (B) Volcano plot showing differentially expressed genes (DEGs) between severe ADHD and healthy control groups. DEGs were defined as Benjamini‐Hochberg (BH)‐adjusted *p* < 0.05 and |log2 fold change| ≥ 0.58. Red and blue dots indicate significantly upregulated and downregulated genes, respectively. (C,D) Gene Ontology (GO) biological process (C) and KEGG pathway (D) enrichment analyses of the DEGs, showing the top 20 enriched terms. Bubble size represents the number of overlapping genes, and color intensity represents −log10(FDR). Representative pathways highlighted in red include regulation of leukocyte activation and phosphatidylinositol phosphorylation in GO analysis, and cytokine–cytokine receptor interaction and PI3K‐Akt signaling in KEGG analysis. (E) Gene set enrichment analysis (GSEA) showing enrichment of the IL‐6 production‐related gene set in severe ADHD (NES = 2.19, FDR = 0.004). (F) Transcript per million (TPM) expression levels of PIK3CA, PIK3R1, AKT1, NFE2L2, and GCLC in peripheral blood RNA‐seq data from healthy controls and children with severe ADHD, showing reduced expression of these PI3K/AKT1/Nrf2 pathway‐related genes in severe ADHD. (G) RT‐qPCR validation of PIK3CA, PIK3R1, AKT1, NFE2L2, and GCLC expression in peripheral blood from mild, moderate, and severe ADHD subgroups. *p* < 0.05; *p* < 0.01t; N.S., not significant.

### 3.3 In Silico Screening Identifies melatonin as a Candidate Protective Modulator of PE Exposure‐Induced Neurotoxicity

Candidate bioactive compounds were screened against the 32 shared genes between PE exposure‐ and ADHD‐related targets using the Drug Signatures Database (DSigDB) via the Enrichr web server [14]. Focusing on well‐documented antioxidant compounds, we selected N‐acetyl‐L‐cysteine, resveratrol, melatonin, glutathione, and curcumin as the five highest‐ranked candidates associated with the target set (Figure 7A,B and Method S5). Their protective activities were then evaluated in HT22 hippocampal neuronal cells exposed to 5 mM glutamate. Concentration‐response analysis yielded EC50 values of approximately 3.12 mM for N‐acetyl‐L‐cysteine, 26.41 µM for resveratrol, 12.27 µM for melatonin, 3.42 mM for glutathione, and 32.15 µM for curcumin (Figure 7C). Melatonin displayed the lowest EC50 among the five candidates and was therefore selected for further mechanistic investigation.

**FIGURE 7:**
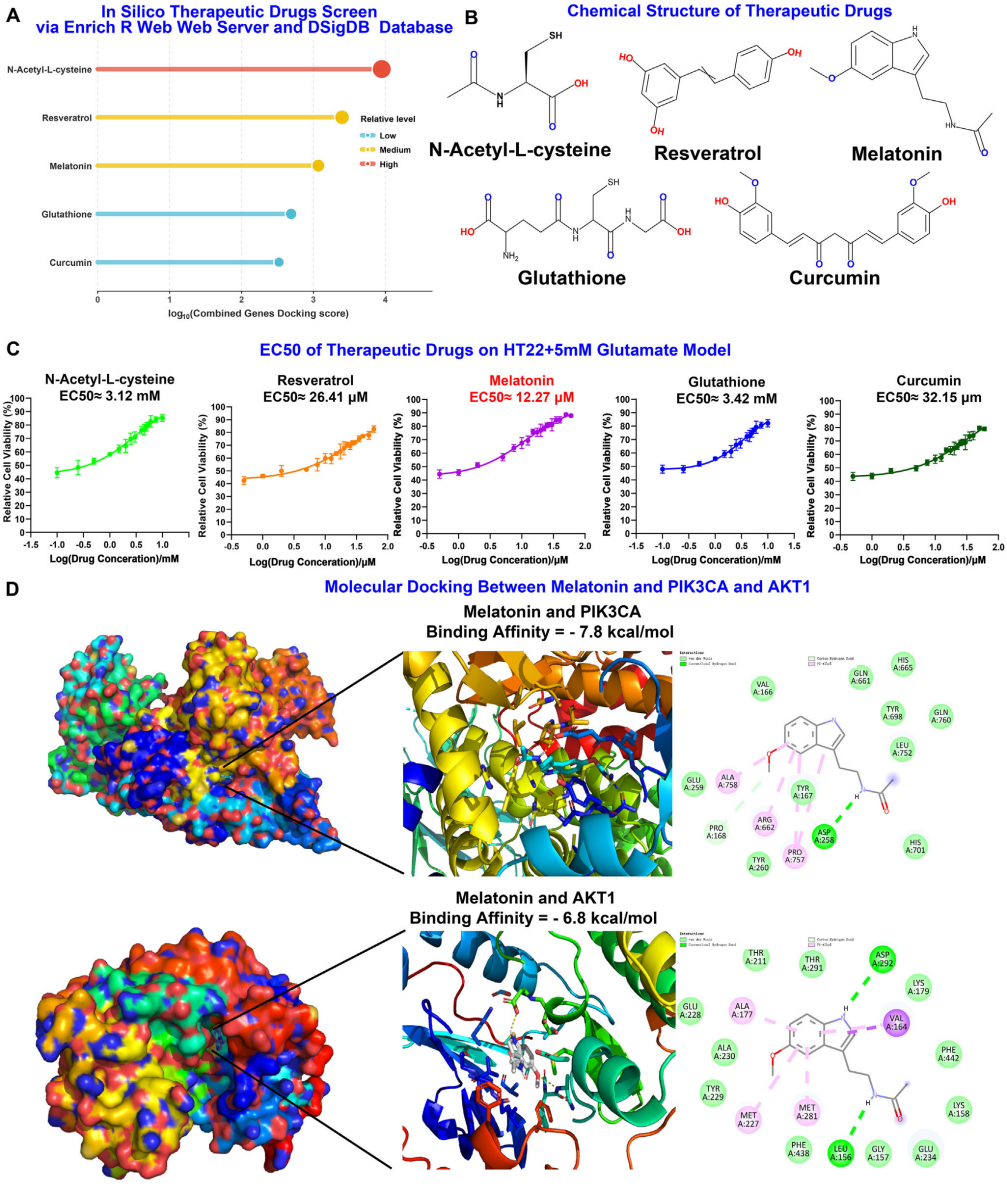
In silico drug screening and experimental validation identify melatonin as a candidate modulator of the PI3K/AKT1 pathway. (A) In silico screening of candidate therapeutic compounds using the Drug Signatures Database (DSigDB) through the Enrichr web server. N‐acetyl‐L‐cysteine, resveratrol, melatonin, glutathione, and curcumin were identified as the top‐ranked candidate compounds based on their enrichment scores. (B) Chemical structures of the five candidate compounds: N‐acetyl‐L‐cysteine, resveratrol, melatonin, glutathione, and curcumin. (C) Dose–response curves and half‐maximal effective concentrations (EC50) of the five candidates in the glutamate‐induced oxidative injury model. HT22 hippocampal neuronal cells were exposed to 5 mM glutamate and treated with increasing concentrations of each compound, and relative cell viability was measured. (D) Molecular docking analysis of melatonin with PIK3CA and AKT1. Protein surface representations, 3D binding conformations, and 2D interaction diagrams illustrate the predicted binding modes of melatonin within the corresponding protein‐binding pockets.

Molecular docking predicted that melatonin occupied binding pockets within both PIK3CA and AKT1, with binding affinities of −7.8 kcal/mol (melatonin‐PIK3CA) and −6.8 kcal/mol (melatonin‐AKT1) (Figure 7D and Method S5). 2D interaction maps revealed multiple hydrogen‐bonding, hydrophobic, and van der Waals interactions between melatonin and residues surrounding the predicted binding sites. The stability of both complexes was further evaluated using 100 ns molecular dynamics simulations. The PIK3CA‐melatonin complex showed an initial conformational adjustment followed by a stable RMSD trajectory (Figure 8A and Method S6), and RMSF analysis identified only localized flexible regions, with most residues maintaining low fluctuations (Figure 8B). The radius of gyration and solvent‐accessible surface area remained within restricted ranges, indicating preserved overall compactness (Figure 8C,D); intermolecular hydrogen bonds were retained intermittently (Figure 8E), and the free‐energy landscape revealed distinct low‐energy conformational basins (Figure 8F). The AKT1‐melatonin complex displayed a comparably stable profile: RMSD remained stable throughout most of the simulation (Figure 8G), residue fluctuations were limited to a few localized peaks (Figure 8H), the radius of gyration and solvent‐accessible surface area were stable over time (Figure 8I,J), one to three intermolecular hydrogen bonds were commonly observed (Figure 8K), and the free‐energy landscape identified a dominant low‐energy basin (Figure 8L). These computational findings supported stable interactions of melatonin with both PIK3CA and AKT1. Based on these analyses, we hypothesized that melatonin alleviates PE exposure‐induced neurotoxicity in ADHD by modulating PI3K/AKT1/Nrf2 signaling.

**FIGURE 8:**
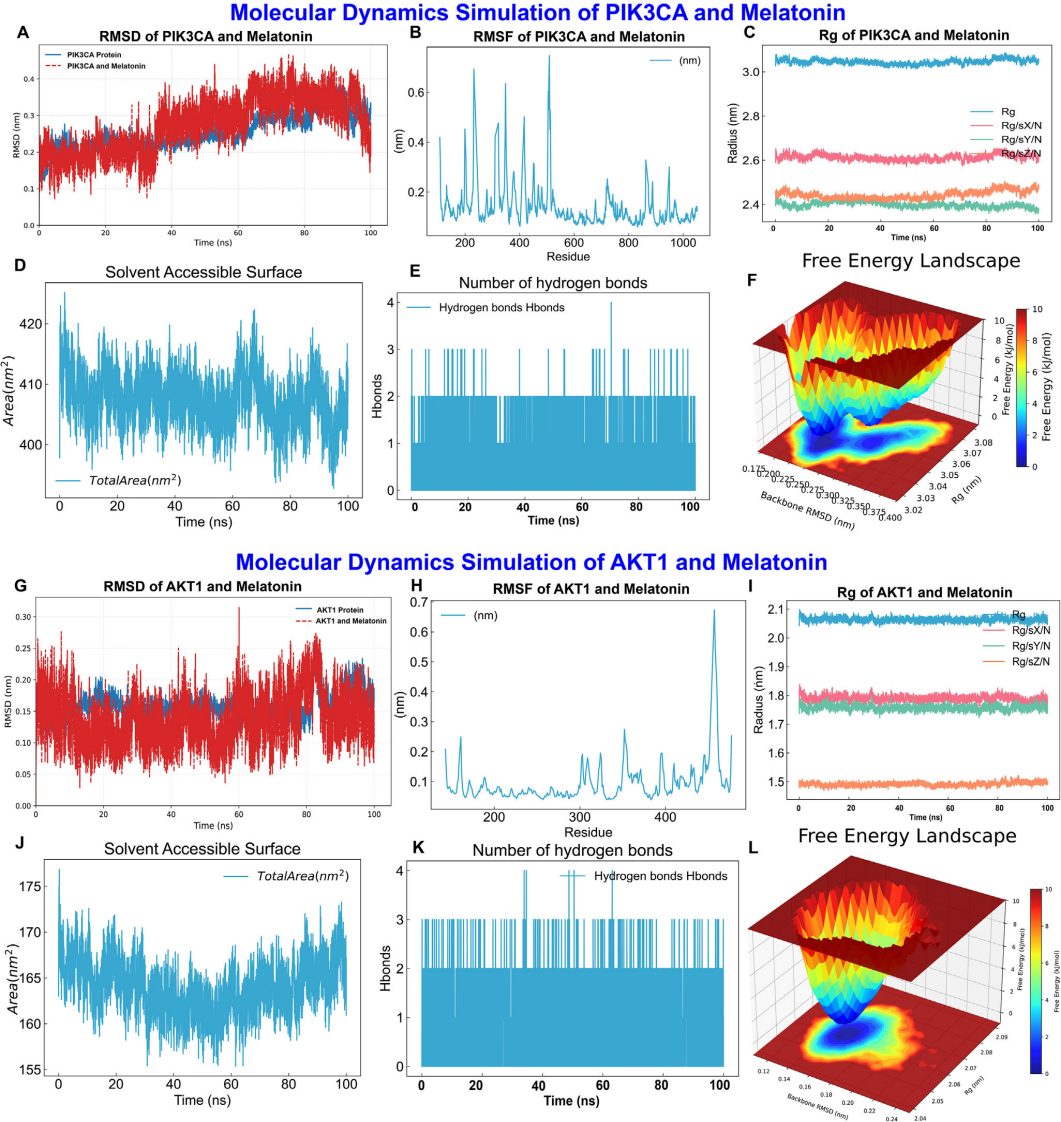
Molecular dynamics simulations of melatonin complexes with PIK3CA and AKT1. (A–F) Molecular dynamics analysis of the PIK3CA–melatonin complex over a 100 ns simulation. (A) Root‐mean‐square deviation (RMSD) trajectories of PIK3CA and the PIK3CA–melatonin complex, reflecting the temporal stability of the protein backbone. (B) Root‐mean‐square fluctuation (RMSF) of individual PIK3CA residues, showing residue‐level structural flexibility during the simulation. (C) Radius of gyration (Rg) and its principal‐axis components, indicating changes in the overall compactness of the PIK3CA–melatonin complex. (D) Solvent‐accessible surface area (SASA) of the PIK3CA–melatonin complex throughout the simulation. (E) Number of intermolecular hydrogen bonds formed between PIK3CA and melatonin as a function of simulation time. (F) Free‐energy landscape of the PIK3CA–melatonin complex constructed using backbone RMSD and Rg as collective variables. Blue regions represent lower‐energy and more stable conformational states, whereas red regions represent higher‐energy states. (G–L) Molecular dynamics analysis of the AKT1–melatonin complex over a 100 ns simulation. (G) RMSD trajectories of apo AKT1 and the AKT1–melatonin complex. (H) Residue‐level RMSF profile of AKT1. (I) Rg and principal‐axis components of the AKT1–melatonin complex. (J) SASA of the AKT1–melatonin complex during the simulation. (K) Number of intermolecular hydrogen bonds between AKT1 and melatonin over time. (L) Free‐energy landscape of the AKT1–melatonin complex based on backbone RMSD and Rg, with lower‐energy conformational basins shown in blue.

### 3.4 Early‐Life PE Exposure Exacerbates ADHD‐Like Behaviors Induced by Perinatal Nicotine Exposure in Mice

Early life represents a critical window of vulnerability to environmental insults in the pathophysiology of ADHD, yet whether early‐life PE exposure exacerbates ADHD‐related phenotypes remains poorly investigated. We first examined whether PE exposure alone was sufficient to induce measurable behavioral abnormalities. In the three‐chamber social interaction test, no significant differences were observed among the Control, Low PE (1 mg/kg), and High PE (3 mg/kg) groups in total distance traveled, sociability preference, or social novelty preference (Figure S5A–C). Consistently, PE exposure alone did not significantly alter 5‐CSRTT performance. Response accuracy and omission rates remained comparable among the three groups across progressively shorter stimulus durations, and no significant differences were observed in premature or perseverative responding across prolonged inter‐trial intervals (Figure S5D–G). These negative‐control findings indicate that, under the present exposure conditions, early‐life PE exposure alone was insufficient to produce overt abnormalities in social behavior, attentional performance, or impulse control, supporting a “second‐hit” model in which PE exposure aggravates a pre‐existing neurodevelopmental vulnerability rather than independently producing an ADHD‐like phenotype. We therefore investigated the effects of early‐life PE exposure in a susceptible background using a perinatal nicotine exposure (PNE) model of ADHD‐like behaviors. Briefly, pregnant C57BL/6J mice received nicotine (100 µg/mL) in drinking water from embryonic day 1.5 to postnatal day (PND) 21, and PE microplastics and melatonin were administered to dams by oral gavage during the same period, such that offspring were exposed through maternal administration from E1.5 to P21 (Figure 9A). Scanning electron microscopy showed that the PE particles used in the experiments had irregular morphologies with a representative size of approximately 5 µm (Figure 9B). Nicotine‐exposed offspring showed significantly lower birth weights than controls (Figure 9C). Growth Curve from PND 0 to PND 21 revealed altered growth trajectories among the groups: nicotine‐exposed offspring initially displayed catch‐up growth and gradually recovered toward normal body weight, whereas PE‐exposed offspring subsequently exhibited significantly higher body weight than controls, an effect potentially related to PE‐induced disruption of lipid metabolism and partially attenuated by melatonin (Figure 9D) [39, 40].

**FIGURE 9:**
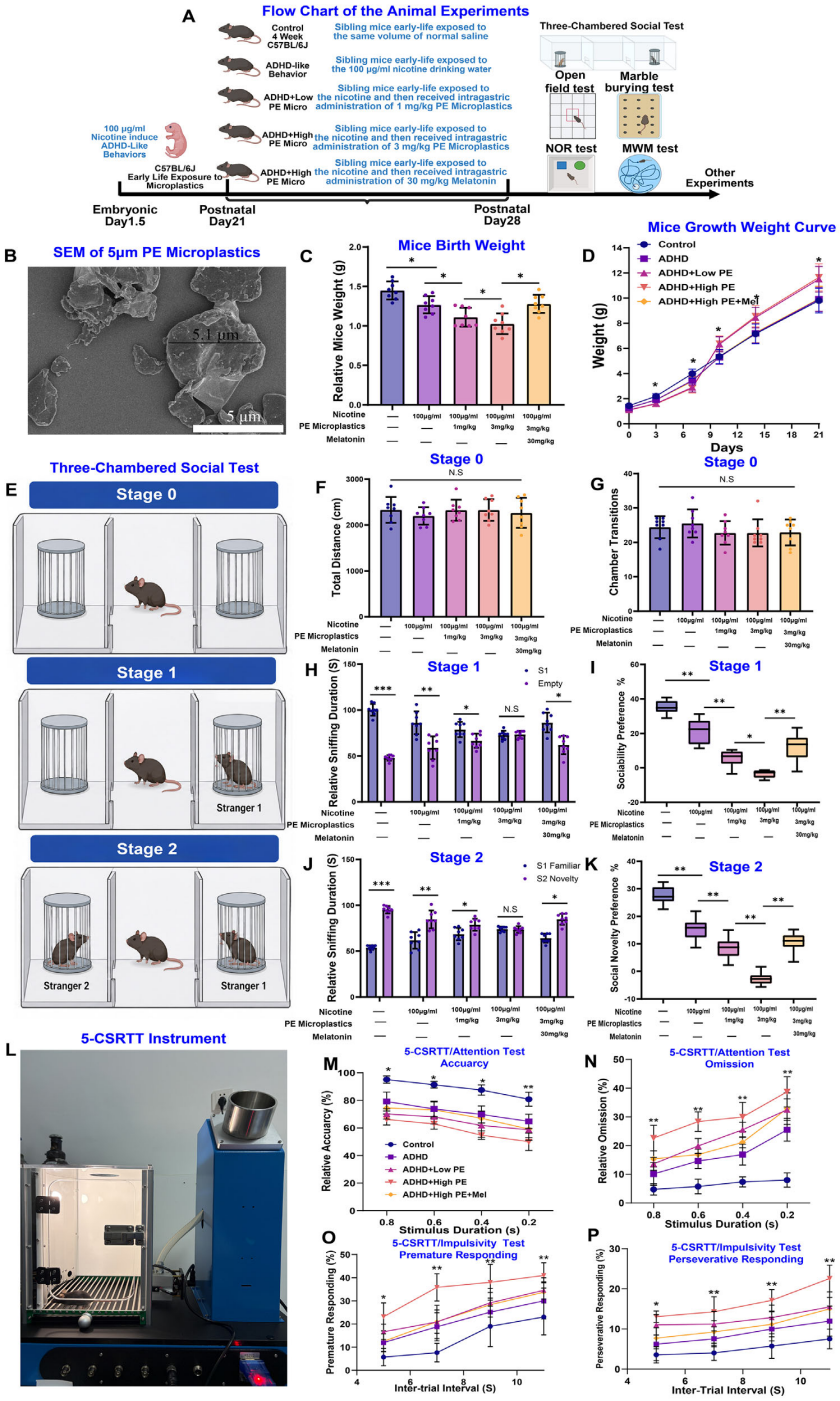
Early‐life PE exposure aggravates behavioral deficits in ADHD‐like mice, and melatonin partially ameliorates these abnormalities. (A) Schematic of the animal experimental design. (B) Scanning electron microscopy image of the 5 µm PE microplastics used in this study. (C) Birth weight of offspring in each group. (D) Growth curves of offspring from postnatal day (P) 0–P21. (E) Schematic of the three‐chamber test, including stage 0 (habituation), stage 1 (sociability), and stage 2 (social novelty). (F‐G) In stage 0, total distance traveled and chamber transitions did not differ among groups, indicating comparable baseline locomotor activity. (H‐I) In stage 1, ADHD‐like mice showed reduced investigation of stranger 1 and a lower sociability preference index than controls; PE co‐exposure further impaired sociability, particularly at the high dose, whereas melatonin partially restored social preference. (J,K) In stage 2, impaired social novelty recognition in ADHD‐like mice was further aggravated by PE and partially rescued by melatonin. (L) Representative image of the 5‐CSRTT apparatus. (M,N) Attentional performance across progressively shorter stimulus durations; PE co‐exposure reduced accuracy and increased omission rates, whereas melatonin partially improved performance. (O,P) Impulsivity‐related performance across prolonged inter‐trial intervals; PE increased premature and perseverative responding, and melatonin partially attenuated these effects. ^*^ *p* < 0.05, ^**^ *p* < 0.01, ^***^ *p* < 0.001; N.S., not significant.

Behavioral characterization was further performed using the three‐chamber social interaction test, open‐field test, marble‐burying test, novel object recognition (NOR) test, Morris water maze (MWM), and five‐choice serial reaction time task (5‐CSRTT) (Methods S7–S12). In the three‐chamber test, no significant differences were observed in total distance traveled or the number of chamber transitions during stage 0, indicating comparable locomotor activity and spontaneous exploration among groups (Figure 9E–G, *p* > 0.05). During the sociability phase, control mice spent substantially more time investigating stranger 1 than the empty cage, whereas this preference was reduced in ADHD‐like mice and further impaired by PE co‐exposure. The ADHD + High PE group showed no clear preference for the social stimulus, while melatonin partially restored social investigation (Figure 9H). Consistently, the sociability preference index progressively decreased across the ADHD, ADHD + Low PE, and ADHD + High PE groups and was significantly improved by melatonin (Figure 9I). During the social novelty phase, control mice preferentially investigated the novel stranger 2 over the familiar stranger 1. This preference was attenuated in ADHD‐like mice and further reduced by PE co‐exposure, with the most pronounced impairment observed in the ADHD + High PE group. Melatonin partially restored preference for the novel mouse (Figure 9J). The social novelty preference index showed a corresponding graded decrease following PE exposure and was partially restored by melatonin treatment (Figure 9K). These findings indicate that early‐life PE exposure aggravates social behavioral abnormalities in PNE‐induced ADHD‐like mice. To more directly assess behavioral domains relevant to the core features of ADHD, we further evaluated attentional performance and impulsive action using the 5‐CSRTT (Figure 9L). Across progressively shorter stimulus durations (0.8, 0.6, 0.4, and 0.2 s), ADHD‐like mice exhibited reduced response accuracy and increased omission rates compared with controls, indicating impaired attentional performance (Figure 9M,N and Figure S6A,B). PE co‐exposure further aggravated these abnormalities in a dose‐dependent manner, with the greatest reduction in accuracy and increase in omissions observed in the ADHD + High PE group. Melatonin partially restored accuracy and reduced omission rates. Impulsive action was subsequently assessed by progressively prolonging the inter‐trial interval from 5 to 11 s. ADHD‐like mice exhibited increased premature responding compared with controls, and PE co‐exposure further increased premature responses, particularly at longer inter‐trial intervals, whereas melatonin partially attenuated this effect (Figure 9O and Figure S6C). Perseverative responding, reflecting impaired response control and repetitive responding, showed a similar exposure‐dependent increase and was also partially reduced by melatonin (Figure 9P and Figure S6D). Collectively, the 5‐CSRTT results provide direct behavioral evidence that early‐life PE exposure exacerbates attentional deficits and impulsive responding in the PNE‐induced ADHD‐like model, thereby extending the behavioral phenotype beyond the social, anxiety‐related, repetitive, and cognitive abnormalities identified using the other behavioral paradigms.

In the open‐field test, representative heatmaps showed progressively reduced exploration of the central area in ADHD‐like and PE‐exposed mice (Figure 10A), whereas mean locomotor velocity did not differ among groups, ruling out motor function bias (Figure 10B). The number of central‐zone entries and the time spent in the center were reduced in ADHD‐like mice, further decreased by PE exposure, and significantly increased by melatonin in the high‐dose PE group (*p* < 0.05; Figure 10C,D), indicating aggravated anxiety‐like behavior. Similarly, in the marble‐burying test, ADHD‐like mice buried more marbles than controls; PE exposure further increased marble burying in a dose‐dependent manner, and melatonin reduced the number of buried marbles in the high‐dose PE group (Figure 10E,F), indicating exacerbated repetitive, compulsive‐like behavior and its attenuation by melatonin. In the NOR test, running speed was comparable among groups (Figure 10G, P > 0.05). Control mice spent significantly more time exploring the novel object than the familiar one; this preference was reduced in ADHD‐like mice and absent in the low‐ and high‐dose PE groups, and melatonin restored preferential exploration of the novel object in high‐dose PE‐exposed mice (Figure 10H). The discrimination index decreased progressively after nicotine and PE exposure, became negative in the high‐dose PE group, and was partially restored by melatonin (Figure 10I, *p* < 0.05). In the Morris water maze, swimming speed did not differ among groups, ruling out motor function bias (Figure 10J). During the five‐day acquisition phase, all groups showed reduced escape latency over time; however, ADHD‐like mice learned more slowly than controls, and PE exposure further prolonged escape latency, particularly from the third training day onward (Figure 10K, P < 0.05). In the probe trial, ADHD‐like mice exhibited fewer platform crossings and less time in the target quadrant; these impairments were aggravated by PE exposure dose‐dependently and partially reversed by melatonin (Figure 10L,M). Representative swimming trajectories corroborated the quantitative findings, with high‐dose PE‐exposed mice displaying less focused search patterns and melatonin‐treated mice showing partial recovery of target‐directed searching (Figure 10N). Collectively, these findings demonstrate that early‐life PE exposure aggravates ADHD‐relevant deficits in attention and impulse control, together with broader abnormalities in social behavior, anxiety‐related behavior, repetitive responding, and cognitive function, whereas melatonin provides partial behavioral protection.

**FIGURE 10:**
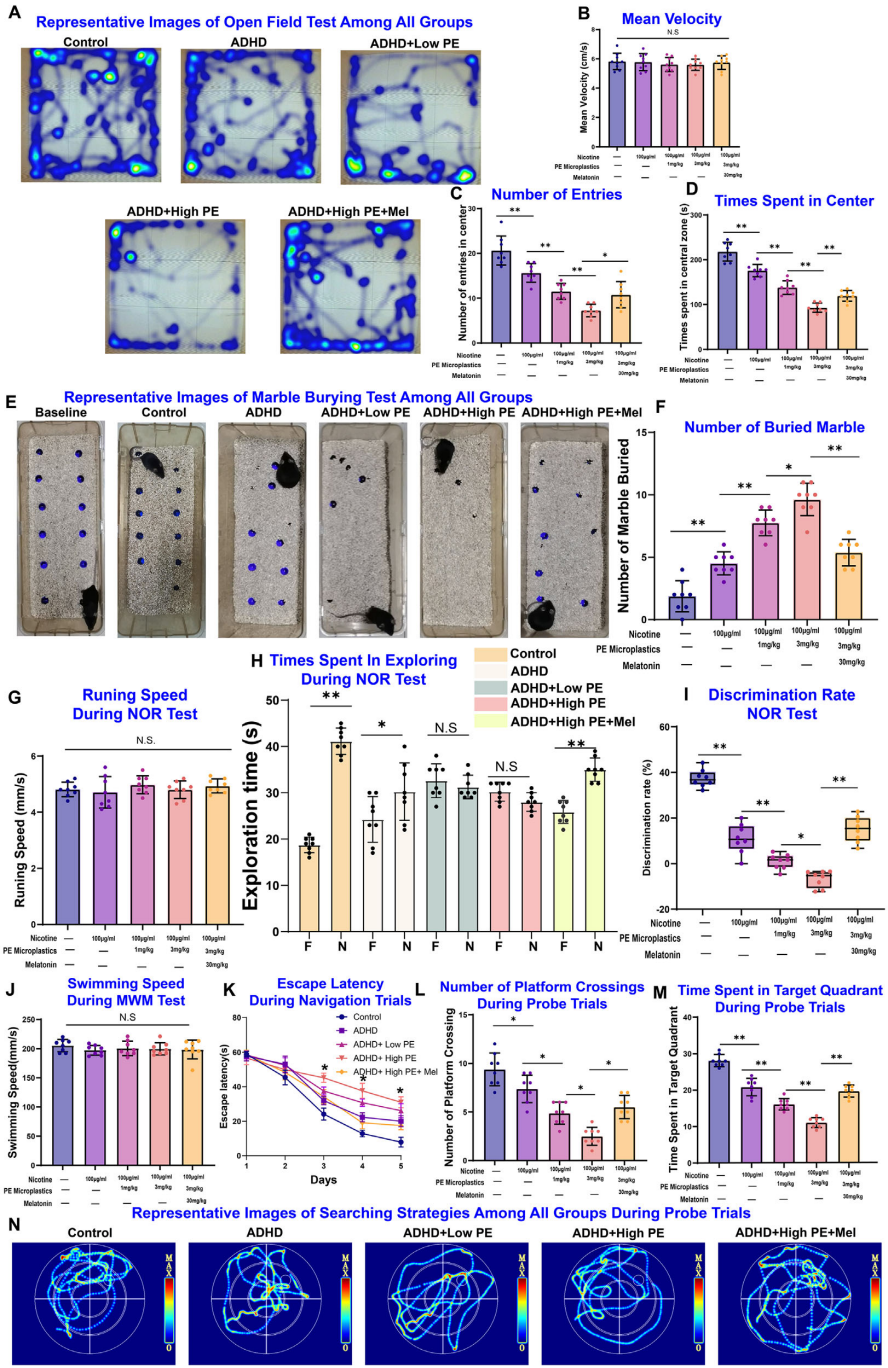
PE microplastic exposure dose‐dependently exacerbates anxiety‐like and cognitive deficits in ADHD‐like mice, and melatonin treatment rescues these impairments. (A–D) Open field test. (A) Representative occupancy heat maps of each group. (B) Mean velocity, showing no significant differences among groups. (C) Number of entries into the center zone and (D) time spent in the center zone, both progressively reduced by nicotine and PE microplastics and partially restored by melatonin, indicating exacerbated anxiety‐like behavior and reduced exploratory drive. (E,F) Marble burying test. (E) Representative images of each group, with the baseline arrangement shown for reference. (F) Number of buried marbles, which increased dose‐dependently with PE exposure and was significantly reduced by melatonin. (G–I) Novel object recognition (NOR) test. (G) Running speed during the test, showing no group differences. (H) Exploration time toward the familiar (F) vs. the novel (N) object in each group. (I) Discrimination rate, which declined dose‐dependently with PE exposure and was restored by melatonin, indicating impaired recognition memory. (J–N) Morris water maze (MWM) test. (J) Swimming speed, showing no group differences. (K) Escape latency during the 5‐day navigation trials; all groups improved over training, but high‐dose PE mice showed significantly delayed learning, which was ameliorated by melatonin. (L) Number of platform crossings and (M) time spent in the target quadrant during the probe trial, both dose‐dependently reduced by PE exposure and restored by melatonin. (N) Representative search‐trajectory heat maps during the probe trial. ^*^ *p* < 0.05, ^**^ *p* < 0.01, ^***^ *p* < 0.001; N.S., not significant.

### 3.5 Early‐Life PE Exposure Aggravates Neuronal Injury, Disrupts Lipid Homeostasis, and Enhances Inflammatory and Oxidative Stress Responses in ADHD‐Like Mice

To unbiasedly delineate the molecular pathways affected by early‐life PE exposure, we performed RNA sequencing of prefrontal cortex (PFC) tissue from ADHD and ADHD + Low PE mice. Principal component analysis showed clear separation between the two groups, indicating that PE exposure substantially altered the prefrontal cortical transcriptome (Figure 11A). Differential expression analysis identified 321 differentially expressed genes, including both upregulated and downregulated transcripts (Figure 11B). GO enrichment analysis indicated that PE exposure affected dopamine receptor signaling and dopamine metabolic processes (Figure 11C), and KEGG analysis further implicated tyrosine metabolism, cytokine–cytokine receptor interaction, and PI3K/AKT signaling (Figure 11D). Gene set enrichment analysis showed significant suppression of glutathione metabolism in the ADHD + Low PE group compared with the ADHD group (Figure 11E), whereas glycerophospholipid metabolism was positively enriched (Figure 11F). These results were consistent with the redox and phospholipid abnormalities detected in the pediatric cohort. RT‐qPCR analysis further showed that NFE2L2 and its downstream antioxidant genes, including GCLC, NQO1, and HO‐1, were significantly reduced in the PFC of ADHD‐like mice. PE exposure produced additional dose‐dependent reductions in these genes, whereas melatonin partially restored their expression in mice exposed to high‐dose PE (Figure 11G–J). These findings indicate that PE exposure suppresses the Nrf2‐dependent antioxidant transcriptional program, consistent with the observed inhibition of glutathione metabolism.

**FIGURE 11:**
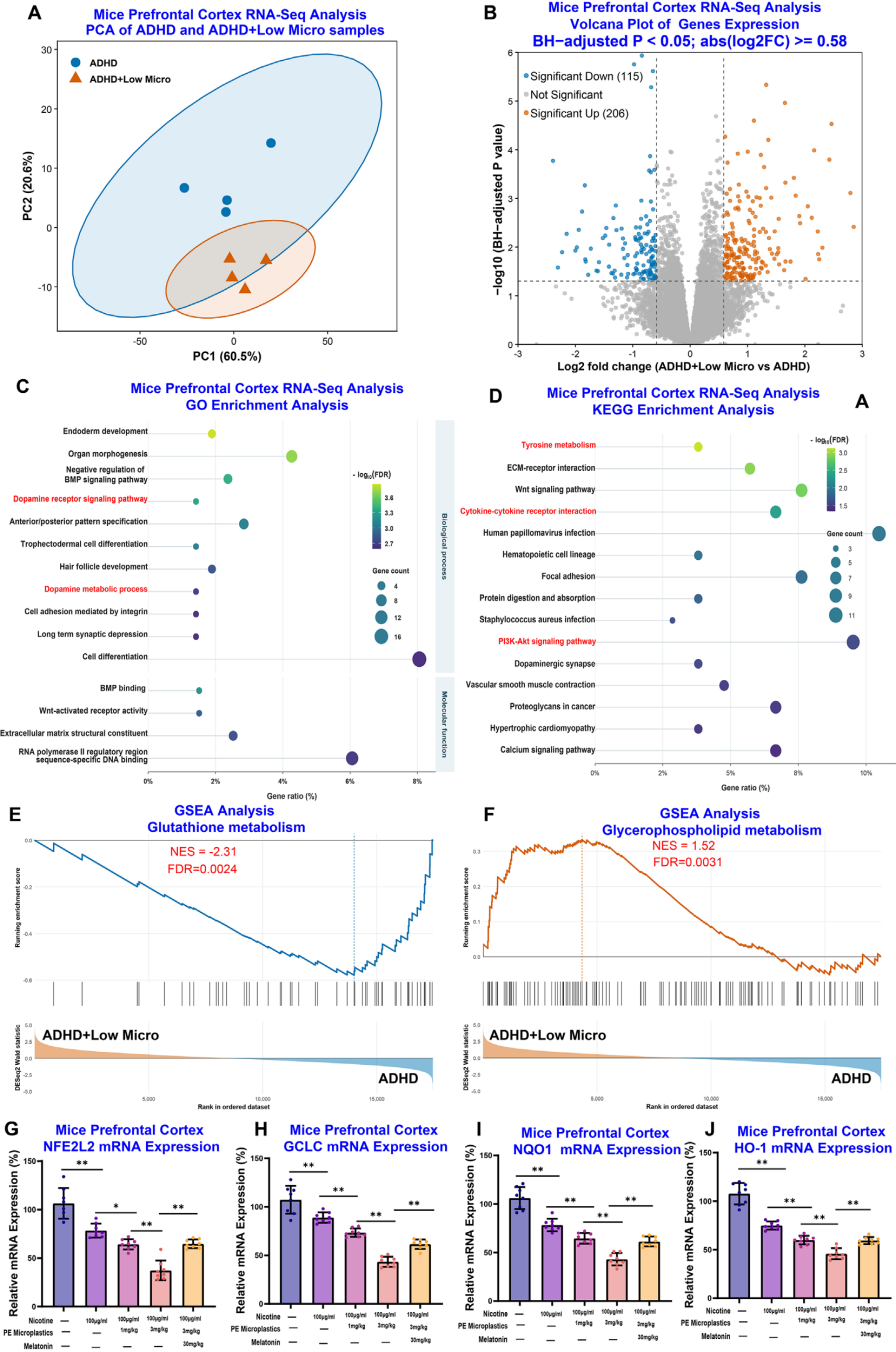
Prefrontal cortex transcriptomic analysis reveals PE microplastic‐induced disruption of redox‐related metabolism and Nrf2 pathway gene expression in ADHD‐like mice. (A) Principal component analysis (PCA) of prefrontal cortex RNA‐sequencing profiles from ADHD‐like mice and ADHD‐like mice exposed to low‐dose PE microplastics, showing clear separation of the two groups. (B) Volcano plot of differentially DEGs in the ADHD plus low‐dose PE group vs. the ADHD group. (C) Gene Ontology (GO) enrichment analysis of DEGs, highlighting dopamine‐related terms including the dopamine receptor signaling pathway and dopamine metabolic process. (D) Kyoto Encyclopedia of Genes and Genomes (KEGG) pathway enrichment analysis, highlighting tyrosine metabolism, cytokine–cytokine receptor interaction, the PI3K‐Akt signaling pathway, and the dopaminergic synapse. (E,F) Gene set enrichment analysis (GSEA) showing significant downregulation of glutathione metabolism and upregulation of glycerophospholipid metabolism in ADHD plus low‐dose PE mice. (G–J) Quantitative reverse‐transcription PCR analysis of Nfe2l2 (G), Gclc (H), Nqo1 (I), and Hmox1 (J) mRNA expression in the prefrontal cortex across the control, ADHD, ADHD plus low‐dose PE, ADHD plus high‐dose PE, and ADHD plus high‐dose PE plus melatonin groups. ADHD‐like mice showed reduced expression of these Nrf2 pathway genes, which was further decreased by PE exposure in a dose‐dependent manner and partially restored by melatonin in high‐dose PE‐exposed mice. Data are presented as mean ± SEM; dots represent individual animals. ^*^ *p* < 0.05, ^**^ *p* < 0.01; N.S., not significant.

Nissl staining demonstrated progressive neuronal disorganization and loss in the hippocampal CA3 and CA1 regions following nicotine and PE exposure (Figure 12A and Method S13). Quantification confirmed significant neuronal loss in ADHD‐like mice, with greater reductions in the PE‐exposed groups, the most pronounced loss in the high‐dose PE group, and partial preservation by melatonin (Figure 12B,C). Hippocampal BDNF protein expression was reduced in ADHD‐like mice, further decreased by PE exposure, and significantly restored by melatonin in the high‐dose PE group (Figure 12D,E, P < 0.05). Serum EPA and DHA levels were reduced by nicotine exposure and declined further after PE administration (Figure 12F,G, P < 0.05), whereas circulating AA, the AA/EPA and AA/DHA ratios (Figure S7A–C, P < 0.05), and phosphatidylcholine hydroperoxide (PCOOH) and 8‐iso‐PGF2α levels increased progressively following nicotine and PE exposure (Figure 12H,I, P < 0.05). Melatonin partially corrected each of these metabolic abnormalities. In the PFC, IL‐1β and TNF‐α (Figure S7D,E) and IL‐6 and MDA levels (Figure 12J,K, P < 0.05) were elevated in ADHD‐like mice and increased further following PE exposure, whereas SOD and GSH levels showed the opposite pattern, with progressive reductions in the PE‐treated groups. Melatonin reduced IL‐6 and MDA levels while restoring SOD and GSH levels in high‐dose PE‐exposed mice (Figure 12L–M, P < 0.05). Together, these findings demonstrate that early‐life PE exposure aggravates neuronal injury, disrupts lipid homeostasis, and intensifies inflammatory and oxidative stress responses in ADHD‐like mice.

**FIGURE 12:**
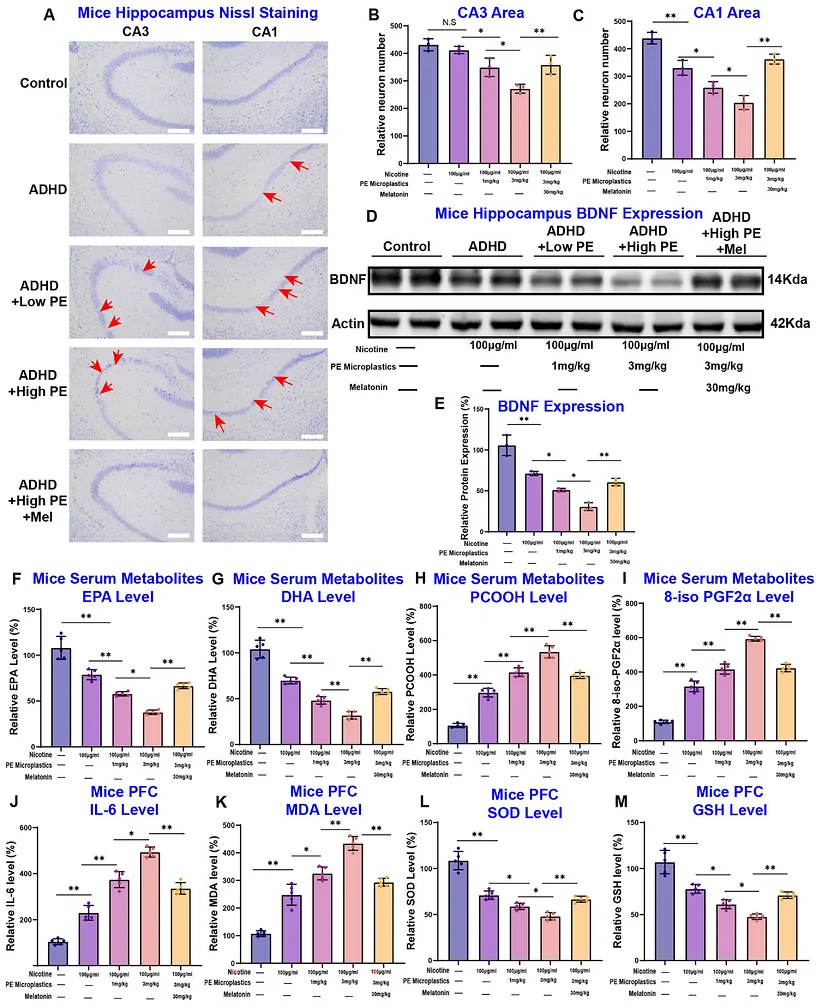
Melatonin attenuates hippocampal neuronal injury, BDNF loss, lipid metabolic abnormalities, inflammation, and oxidative stress induced by polyethylene microplastic exposure in ADHD‐like mice. (A) Representative Nissl‐stained images of the hippocampal CA3 and CA1 regions from different groups. Red arrows indicate areas of neuronal loss, disorganization, or morphological damage. (B,C) Quantification of relative neuronal numbers in the hippocampal CA3 (B) and CA1 (C) regions. Nicotine‐induced ADHD‐like mice showed reduced neuronal numbers, which were further decreased by PE exposure in a dose‐dependent manner. Melatonin treatment partially restored neuronal numbers in mice exposed to high‐dose PE. (D) Representative Western blot bands showing hippocampal brain‐derived neurotrophic factor (BDNF) protein expression, with actin used as the loading control. (E) Quantification of BDNF protein expression. BDNF expression was reduced in ADHD‐like mice and further suppressed by PE exposure, whereas melatonin partially restored its expression. (F–I) Relative serum levels of eicosapentaenoic acid (EPA; F), docosahexaenoic acid (DHA; G), phosphatidylcholine hydroperoxide (PCOOH; H), and 8‐isoprostaglandin F2α (8‐iso‐PGF2α; I). Nicotine exposure reduced EPA and DHA levels and increased PCOOH and 8‐iso‐PGF2α levels. These alterations were further aggravated by PE exposure, particularly at the high dose, and were partially reversed by melatonin treatment. (J–M) Levels of interleukin‐6 (IL‐6; J), malondialdehyde (MDA; K), superoxide dismutase (SOD; L), and reduced glutathione (GSH; M) in the prefrontal cortex. PE exposure dose‐dependently enhanced the nicotine‐induced increases in IL‐6 and MDA and further reduced SOD and GSH levels. Melatonin treatment significantly attenuated these inflammatory and oxidative stress alterations ^*^ *p* < 0.05, ^**^ *p* < 0.01, N.S., not significant.

### 3.6 PE Exposure Suppresses PI3K/AKT1/Nrf2 Signaling and Antioxidant Defenses in ADHD‐Like Mice, and Melatonin Reactivates the Axis

We first examined whether PE exposure altered dopamine metabolism in the PFC. Dopamine and norepinephrine levels were significantly reduced in ADHD‐like mice and declined further following PE exposure in a dose‐dependent manner (Figure 13A,B, *p* < 0.05), and melatonin partially restored both neurotransmitters in high‐dose PE‐exposed mice. In contrast, DOPAC levels remained largely unchanged among groups (Figure 13C, *p* > 0.05). Consequently, the DOPAC/DA ratio increased following nicotine exposure and rose further after PE administration, indicating accelerated dopamine turnover relative to the diminished dopamine pool, and melatonin significantly reduced the elevated ratio (*p* < 0.05; Figure 13D). Western blot analysis further showed that tyrosine hydroxylase and dopamine D1 receptor protein levels were reduced in ADHD‐like mice, further suppressed by PE exposure with the greatest reductions in the high‐dose group, and partially restored by melatonin (Figure 13E–G). These findings indicate that PE exposure aggravates prefrontal dopaminergic dysfunction in ADHD‐like mice, whereas melatonin partially preserves dopamine synthesis and receptor signaling.

**FIGURE 13:**
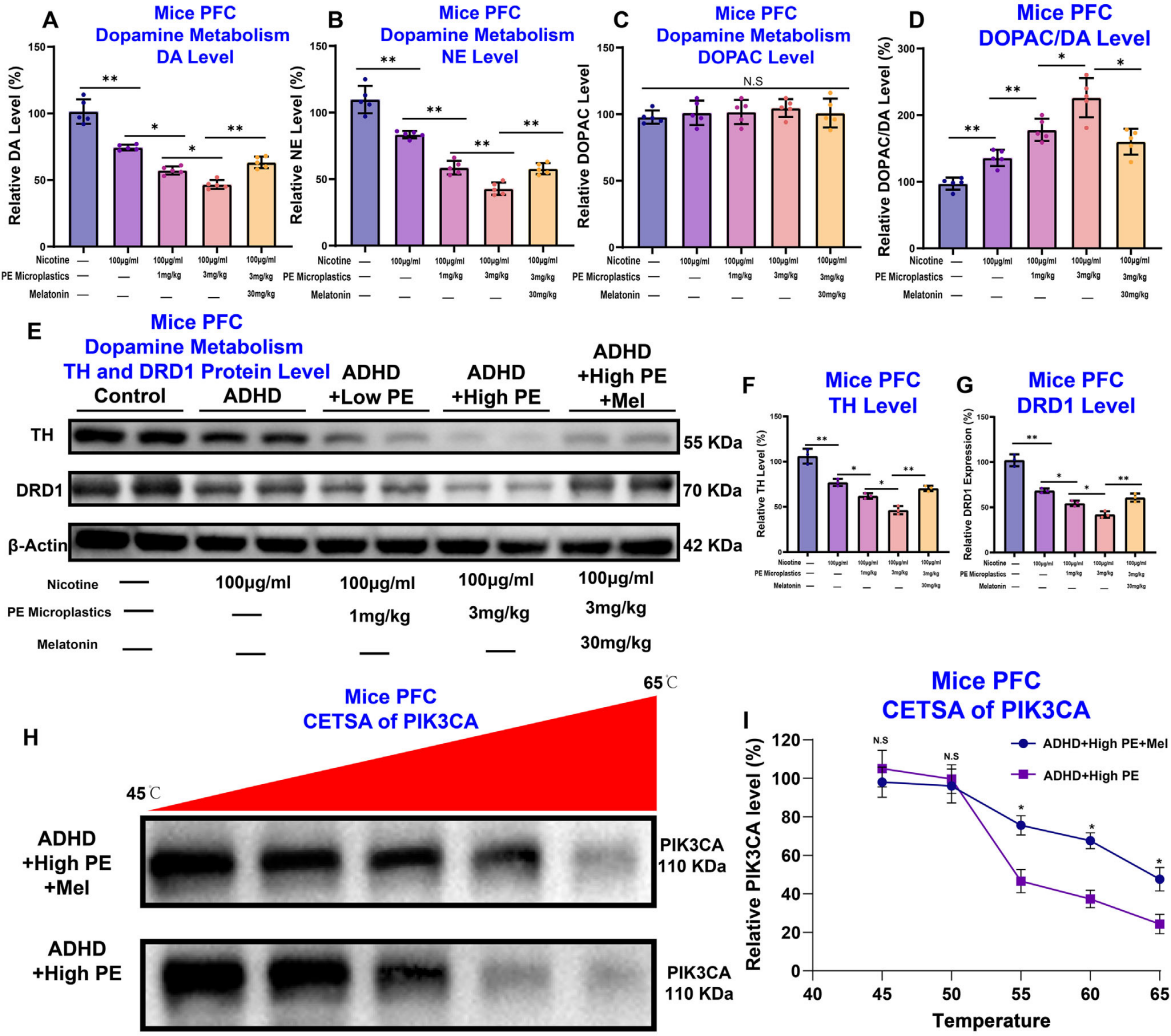
Melatonin restores prefrontal cortical dopaminergic homeostasis and enhances the thermal stability of PIK3CA in PE‐exposed ADHD‐like mice. (A–D) Relative levels of dopamine (DA; A), norepinephrine (NE; B), 3,4‐dihydroxyphenylacetic acid (DOPAC; C), and the DOPAC/DA ratio (D) in the prefrontal cortex (PFC). (E–G) Representative Western blots (E) and quantitative analysis of tyrosine hydroxylase (TH; F) and dopamine receptor D1 (DRD1; G) protein expression in the PFC. (H–I) Cellular thermal shift assay (CETSA) of the PI3K catalytic subunit p110α (PIK3CA) using a PIK3CA‐specific antibody. (H) Representative immunoblots of soluble PIK3CA after heating at 45°C, 50°C, 55°C, 60°C, and 65°C. (I) Thermal stability curves showing greater retention of soluble PIK3CA in the ADHD + High PE + Mel group than in the ADHD + High PE group at 55°C, 60°C, and 65°C, consistent with enhanced PIK3CA thermal stability following melatonin administration. These findings support cellular engagement of PIK3CA but do not by themselves establish direct molecular binding. ^*^ *p* < 0.05; N.S., not significant.

We next investigated whether the protective effects of melatonin were associated with engagement of the PI3K catalytic subunit p110α (PIK3CA). CETSA was performed using a PIK3CA/p110α‐specific antibody. In PFC lysates from high‐dose PE‐exposed ADHD‐like mice, the soluble PIK3CA fraction progressively decreased as the temperature increased from 45°C to 65°C (Figure 13H and Method S14). Samples from melatonin‐treated mice retained significantly higher levels of soluble PIK3CA at 55°C, 60°C, and 65°C than those from untreated mice (Figure 13I, *p* < 0.05), whereas no significant differences were observed at 45°C or 50°C. These findings indicate increased thermal stability of PIK3CA following melatonin administration and are consistent with cellular engagement of the p110α catalytic subunit. Together with the molecular docking and molecular dynamics results, the CETSA findings support PIK3CA as a candidate molecular target associated with melatonin‐mediated activation of PI3K/AKT1 signaling. Western blot analysis further showed that phosphorylation of the PI3K p85 regulatory subunit at Tyr458, AKT1 at Ser473, and GSK3β at Ser9 was reduced in the PFC of ADHD‐like mice and further suppressed by PE exposure in a dose‐dependent manner (Figure 14A,B, *p* < 0.05), whereas total PI3K p85, AKT1, and GSK3β protein abundance remained largely unchanged. Melatonin significantly restored phosphorylation of all three signaling proteins in high‐dose PE‐exposed mice. Because phosphorylation of GSK3β at Ser9 inhibits its kinase activity, restoration of AKT1‐dependent GSK3β phosphorylation is consistent with reduced GSK3β‐mediated suppression of Nrf2 signaling. Consistent with this interpretation, Keap1 protein abundance increased following nicotine exposure and rose further after PE co‐exposure, whereas Nrf2 expression progressively decreased (Figure 14C,D and Method S15, *p* < 0.05). Melatonin reduced Keap1 abundance while partially restoring Nrf2 expression. In parallel, the mRNA levels of the Nrf2 downstream antioxidant genes Gclc, Nqo1, and Hmox1 were reduced in ADHD‐like mice, further suppressed by PE exposure, and significantly restored by melatonin in the high‐dose PE group (Figure 14E–G, *p* < 0.05). These findings indicate that PE co‐exposure suppresses PI3K/AKT1/GSK3β/Nrf2 signaling in the PFC, whereas melatonin partially restores pathway activity and Nrf2‐dependent antioxidant defenses. To determine whether these molecular alterations were restricted to the PFC or extended to another ADHD‐relevant dopaminergic region, we additionally examined key dopaminergic and signaling proteins in the striatum. Striatal TH and DRD1 protein expression was reduced in ADHD‐like mice and further decreased following PE co‐exposure, whereas melatonin partially restored both proteins (Figure S8A,B, *p* < 0.05). Consistent with the PFC findings, phosphorylation of PI3K p85, AKT1, and GSK3β was progressively reduced following nicotine and PE co‐exposure, while total PI3K p85, AKT1, and GSK3β protein abundance remained largely unchanged (Figure S8C,D). Melatonin partially restored phosphorylation of these signaling proteins. In parallel, striatal Keap1 expression increased, and Nrf2 expression decreased following nicotine exposure and were further altered by PE co‐exposure, whereas melatonin partially reversed these changes (Figure S8E,F). These findings indicate that PE‐associated dopaminergic dysfunction and suppression of PI3K/AKT1/Nrf2‐related signaling are not restricted to the PFC but also extend to the striatum, suggesting broader involvement of ADHD‐relevant dopaminergic brain regions.

**FIGURE 14:**
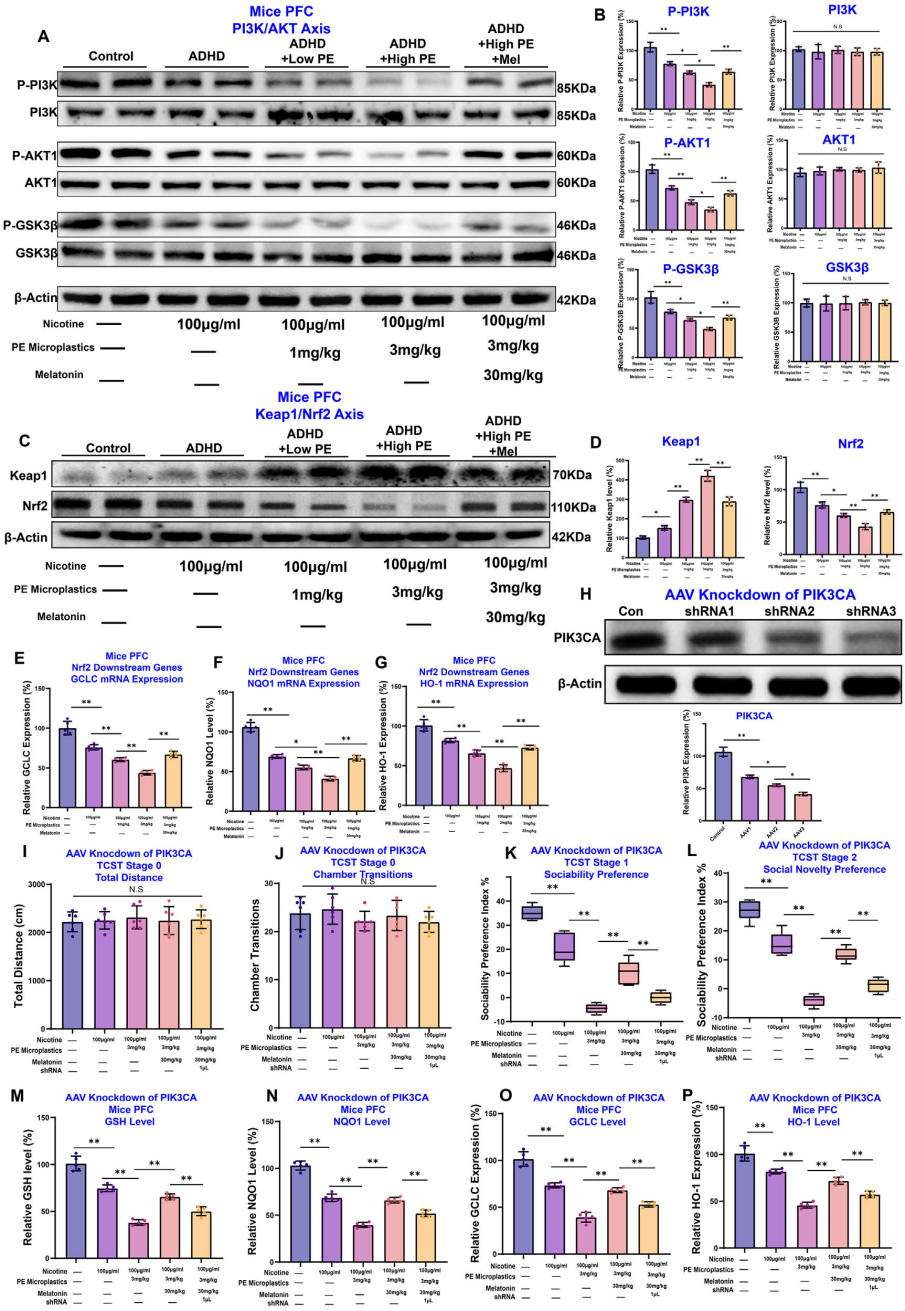
Melatonin modulates the PI3K/AKT1/Nrf2 axis in the prefrontal cortex, and AAV‐mediated PI3K knockdown attenuates its protective effects in PE‐exposed ADHD‐like mice. (A,B) Representative Western blots (A) and quantification (B) of the PI3K/AKT axis in the PFC across the five groups. Nicotine exposure reduced the phosphorylation levels of PI3K, AKT1, and GSK3β without significantly altering their total protein abundance. PE co‐exposure further suppressed phosphorylation in a dose‐dependent manner, whereas melatonin partially restored the phosphorylation of these signaling proteins. (C,D) Western blot analysis (C) and quantification (D) of the Keap1/Nrf2 axis. Keap1 increased while Nrf2 decreased dose‐dependently with PE exposure, and melatonin reversed both changes. (E–G) qRT‐PCR analysis of Nrf2 downstream antioxidant genes in the PFC: GCLC (E), NQO1 (F), and HO‐1 (G) mRNA levels were dose‐dependently suppressed by PE and restored by melatonin. (H) Validation of AAV‐shRNA‐mediated PI3K knockdown in the PFC. (I–L) Three‐chambered social test (TCST) in the PI3K‐knockdown cohort. Total distance (I) and chamber transitions (J) in Stage 0 showed no group differences. Melatonin restored the sociability preference (K, Stage 1) and social novelty preference (L, Stage 2) in ADHD + High PE mice, and these protective effects were markedly attenuated by PIK3CA knockdown. (M–P) PFC levels of reduced glutathione (GSH; M) and the Nrf2‐regulated antioxidant factors NQO1 (N), GCLC (O), and HO‐1 (P) following AAV‐mediated PIK3CA knockdown. Melatonin increased GSH levels and restored NQO1, GCLC, and HO‐1 expression in high‐dose PE‐exposed ADHD‐like mice. These effects were significantly attenuated by PI3K knockdown, supporting the involvement of PI3K‐dependent Nrf2 signaling in the antioxidant and behavioral effects of melatonin.

Finally, we examined whether reducing PIK3CA expression in the PFC would attenuate the behavioral and antioxidant effects of melatonin. Three AAV‐delivered shRNA constructs targeting PIK3CA, which encodes the p110α catalytic subunit of class IA PI3K, were evaluated (Method S16). All three constructs reduced p110α protein expression, and shRNA3, which produced the greatest knockdown efficiency, was selected for subsequent experiments (Figure 14H). PIK3CA knockdown did not significantly affect total distance traveled or chamber transitions during stage 0 of the three‐chamber test, indicating preserved general locomotor activity (Figure 14I,J). Melatonin significantly improved both sociability and social novelty preference in high‐dose PE‐exposed ADHD‐like mice, whereas these improvements were markedly attenuated following PIK3CA knockdown (Figure 14K,L and Method S17). Similarly, melatonin restored PFC GSH levels and increased NQO1, GCLC, and HO‐1 expression, and each of these effects was significantly weakened by PIK3CA knockdown (Figure 14M,P). Collectively, these findings indicate that PE exposure disrupts prefrontal dopamine homeostasis and suppresses PI3K/AKT1/Nrf2 signaling in ADHD‐like mice, whereas melatonin partially restores pathway activity, Nrf2‐dependent antioxidant defenses, and behavioral function. The attenuation of these protective effects following PIK3CA knockdown provides functional evidence that PIK3CA signaling contributes to the protective actions of melatonin. However, because the knockdown was incomplete and was not restricted to a specific neural cell population, these experiments do not establish complete pathway necessity or identify the neuronal or glial populations responsible for the observed effects.

## 4 Discussion

In the contemporary industrialized world, humans are immersed in a pervasive plastic environment. Microplastics, defined as synthetic polymer particles smaller than 5 mm, have become ubiquitous environmental contaminants and have been detected in human blood, placenta, brain tissue, and numerous other biological matrices [41]. Among the polymers identified through human biomonitoring, polyethylene (PE) is consistently the most frequently detected, accounting for approximately 30% of blood‐borne microplastics in recent studies [3], a finding attributable to its status as the highest‐volume plastic produced globally and its predominant use in food packaging, toys, feeding bottles, and household items that constitute major exposure sources during early childhood [42]. Children represent a uniquely vulnerable population owing to higher intake relative to body mass, immature detoxification and immune systems, hand‐to‐mouth behaviors, and critical windows of neurodevelopment [20]. Despite this biological plausibility, the relevance of circulating microplastics to childhood neurodevelopmental disorders [43], particularly ADHD, remains poorly understood, with existing epidemiological evidence limited to a small number of cross‐sectional studies that preclude causal inference. In our pilot cohort, PE showed the most prominent increase among the detected polymers in children with ADHD, and both its relative abundance and blood concentration, rather than particle size, were associated with ADHD status [44]. Circulating PE concentrations also increased across the mild, moderate, and severe clinical subgroups and correlated positively with SNAP‐IV and Conners T‐scores, indicating a severity‐associated gradient. Although this pattern strengthens the consistency of the observed association, it should not be interpreted as evidence that PE exposure causes greater ADHD severity. The predominance of PE in our cohort is broadly consistent with biomonitoring studies reporting PE as one of the most abundant polymers detected in human blood, stool, and tissue samples [45]. Notably, PE particle‐size distributions did not differ significantly between children with ADHD and healthy controls. Because polymers differ in surface chemistry, hydrophobicity, and additive composition, biological interactions may depend not only on particle size but also on polymer type, total burden, and associated chemical constituents [1]. Taken together, our pilot pediatric cohort provides preliminary evidence that circulating PE burden is associated with ADHD‐related symptom severity and accompanying redox‐inflammatory alterations. However, the cross‐sectional design precludes conclusions regarding temporality or causality, and these observations should be considered hypothesis‐generating and supportive of further prospective and experimental investigation [46].

Oxidative stress and neuroinflammation represent a convergent pathophysiological node where microplastic toxicity and ADHD biology intersect. Microplastics induce toxicity through a well‐characterized oxidative cascade: particles accumulate in mitochondria, disrupt the electron transport chain, depolarize mitochondrial membranes, and trigger ROS overproduction, leading to protein oxidation and lipid peroxidation [47]. Consistent with this, even low‐dose polystyrene microplastic exposure induced hepatotoxicity with compromised antioxidant balance and elevated cytokines in human cell lines and organoids [48, 49]. In parallel, a recent meta‐analysis demonstrated that children with ADHD have significantly elevated blood total oxidant status and reduced total antioxidant status compared with healthy controls [9], and circulating cytokines are likewise elevated [50]. Our cohort findings align with this pattern: children with ADHD exhibited reduced GSH levels, increased MDA levels, and elevated IL‐6, with the most pronounced alterations in the severe subgroup. The metabolomic alterations in our cohort further converge on a coherent lipid‐redox disruption cascade. Children with ADHD exhibited reduced EPA and DHA alongside elevated AA/EPA and AA/DHA ratios, consistent with prior meta‐analytic evidence identifying omega‐3 PUFA deficiency as a reproducible biochemical feature of ADHD. Because EPA and DHA serve as precursors for pro‐resolving lipid mediators, whereas AA is the primary substrate for pro‐inflammatory eicosanoids and non‐enzymatic lipid peroxidation, an elevated AA/EPA ratio reflects a membrane environment that favors inflammation over resolution [51, 52]. This pro‐oxidant shift was further evidenced by increased 8‐iso‐PGF2α, a stable F2‐isoprostane generated by free radical‐mediated peroxidation of membrane AA and widely regarded as a reliable in vivo biomarker of oxidative stress [51, 52]. Notably, 8‐iso‐PGF2α is not merely a passive marker but also exerts biological activity as a vasoconstrictor and platelet activator, suggesting that its elevation may actively contribute to neurovascular pathophysiology [53]. Taken together, these findings support a self‐perpetuating model: microplastic‐induced ROS deplete membrane omega‐3 PUFAs and oxidize AA‐enriched glycerophospholipids, generating products such as 8‐iso‐PGF2α that amplify neuroinflammation, which in turn produces further ROS, perpetuating membrane damage and exacerbating the omega‐3‐deficient, pro‐inflammatory lipid profile characteristic of ADHD [54, 55].

Several independent lines of evidence converged on PI3K/AKT1/Nrf2 signaling as a candidate pathway of interest in the biological alterations associated with PE exposure and ADHD. Network toxicology identified AKT1, PIK3CA, NFE2L2, and IL6 as central genes shared between PE‐related and ADHD‐related gene sets, whereas Mendelian randomization showed a nominal inverse association between genetically predicted AKT1 expression and ADHD risk. Importantly, the MR analysis evaluates the relationship between genetically proxied gene expression and ADHD susceptibility and does not directly assess PE exposure or establish that AKT1 mediates the association between PE exposure and ADHD. Accordingly, the MR findings should be interpreted as supportive evidence for AKT1 target prioritization and mechanistic triangulation, rather than as proof that PE exposure exerts its effects through AKT1 in humans. Importantly, after transcriptome‐wide multiple‐testing correction, peripheral blood transcriptomic analysis continued to identify significant alterations in inflammatory and phosphatidylinositol‐related pathways, including cytokine–cytokine receptor interaction and PI3K‐Akt signaling. Reduced expression of PIK3CA, PIK3R1, AKT1, NFE2L2, and GCLC was most evident in the severe ADHD subgroup, further supporting disruption of PI3K/AKT1/Nrf2‐related signaling in children with greater disease burden. Independent analysis of the public whole‐blood dataset GSE159104 provided complementary support for this redox‐metabolic framework. FDR‐corrected enrichment analysis highlighted peroxisome‐related processes, together with alterations in ribosomal pathways. Peroxisomes are key organelles involved in both ROS generation and ROS clearance, as well as lipid metabolism, and disruption of peroxisomal homeostasis can shift intracellular redox balance toward oxidative stress. Because PI3K/AKT and Nrf2 are redox‐sensitive signaling systems, persistent ROS imbalance provides a plausible biological bridge between the peroxisomal alterations observed in the independent dataset and the PI3K/AKT1/Nrf2 dysregulation identified in our cohort. Thus, although GSE159104 does not directly replicate the PI3K/AKT1/Nrf2 signature, it independently supports the broader oxidative and metabolic context in which this signaling abnormality occurs. This convergence provided the rationale for systematically screening antioxidant compounds targeting this axis [14]. Among the five candidates evaluated, melatonin exhibited the lowest effective concentration and rescued the behavioral, molecular, and metabolic abnormalities induced by PE exposure in ADHD‐model mice. The neuroprotective effects of melatonin through PI3K/AKT/Nrf2 signaling have been independently documented in multiple neurological disease models. In paraquat‐induced Parkinson's disease, melatonin protected dopaminergic neurons via PI3K/AKT/Nrf2 activation [56]. In traumatic brain injury, melatonin promoted Nrf2 nuclear translocation and antioxidant enzyme induction, effects that were partially reversed in Nrf2‐knockout mice [57]. In cerebral ischemia‐reperfusion injury, melatonin attenuated brain edema by upregulating p‐PI3K, p‐AKT, and Nrf2 expression [58]. These precedents lend external plausibility to our findings and position the present study as the first to extend this mechanism to environmental microplastic exposure in the context of ADHD‐like phenotypes. Importantly, the timing of melatonin administration should be distinguished from a therapeutic treatment paradigm. In the present study, melatonin was administered concurrently with nicotine and PE throughout the developmental exposure period from E1.5 to P21, rather than being initiated after establishment of the ADHD‐like phenotype. Accordingly, our findings demonstrate a preventive or protective effect of melatonin against the development and aggravation of PE‐associated neurobehavioral and molecular abnormalities, but do not establish therapeutic efficacy for reversing an already established ADHD‐like phenotype.

Maternal oxidative stress and inflammation during pregnancy are independently associated with adverse neurodevelopmental outcomes in offspring [59]. To validate the convergent human and computational evidence in vivo, we used perinatal nicotine exposure to establish a neurodevelopmentally vulnerable background and examined whether early‐life PE exposure aggravated ADHD‐related phenotypes. Importantly, PE‐only exposure in a separate cohort did not produce detectable abnormalities in either the three‐chamber social interaction test or the 5‐CSRTT, including accuracy, omission rate, premature responding, and perseverative responding [60]. These findings support a second‐hit model in which PE exposure amplifies a pre‐existing neurodevelopmental vulnerability rather than independently producing an overt ADHD‐like phenotype under the present exposure conditions. The addition of the 5‐CSRTT substantially strengthened the behavioral characterization of the model by directly assessing attentional performance and impulsive action, two domains closely aligned with the core clinical features of ADHD. ADHD‐like mice exhibited reduced accuracy and increased omission rates as stimulus duration shortened, together with increased premature responding as the inter‐trial interval was prolonged. PE co‐exposure further aggravated these abnormalities in a dose‐dependent manner, whereas melatonin partially improved attentional performance and reduced impulsive responding. Perseverative responding was also increased after PE exposure, indicating impaired response control and enhanced repetitive responding. These 5‐CSRTT findings extend the behavioral effects of PE beyond the broader social, anxiety‐related, repetitive, recognition‐memory, and spatial‐learning abnormalities observed in the other behavioral paradigms. The behavioral abnormalities were accompanied by disruption of catecholaminergic signaling. Prefrontal dopamine and norepinephrine signaling is central to attention, inhibitory control, and working memory, and its disturbance represents an important neurobiological feature of ADHD [61]. In ADHD‐like mice, dopamine and norepinephrine levels were reduced and declined further following PE co‐exposure, together with dose‐dependent reductions in tyrosine hydroxylase and dopamine D1 receptor expression. Notably, DOPAC levels remained relatively stable whereas the DOPAC/DA ratio increased. This pattern is more appropriately interpreted as increased dopamine turnover relative to a progressively depleted dopamine pool rather than as an absolute increase in dopamine metabolism [62]. Melatonin partially restored catecholamine levels, normalized the DOPAC/DA ratio, and recovered TH and DRD1 expression. Consistent dopaminergic alterations were also observed in the striatum, where TH and DRD1 expression declined progressively following nicotine and PE co‐exposure and was partially restored by melatonin. Importantly, striatal phosphorylation of PI3K, AKT1, and GSK3β showed changes parallel to those observed in the PFC, accompanied by increased Keap1 and reduced Nrf2 expression. These findings indicate that PE‐associated dopaminergic and redox‐signaling abnormalities are not restricted to the PFC but extend to the striatum, suggesting a broader effect across ADHD‐relevant dopaminergic regions. Nissl staining and BDNF analysis further demonstrated neuronal injury, with PE exposure aggravating neuronal loss in the hippocampal CA1 and CA3 regions and reducing BDNF expression. The parallel improvement in BDNF abundance, neuronal integrity, and behavioral performance following melatonin supports a relationship among redox homeostasis, neurotrophic support, and neurobehavioral function. The CETSA analysis further strengthened the target‐level consistency of the mechanistic evidence. CETSA specifically assessed the p110α catalytic subunit encoded by PIK3CA, which was also the molecular target evaluated by molecular docking, molecular dynamics simulations, and AAV‐mediated knockdown. Melatonin increased the thermal stability of PIK3CA at higher temperatures, providing cellular evidence consistent with engagement of the p110α catalytic subunit. Importantly, CETSA reflects ligand‐associated or interaction‐associated protein stabilization and does not independently establish direct physical binding. Nevertheless, the convergence of molecular docking, molecular dynamics, PIK3CA‐specific CETSA, and functional PIK3CA knockdown supports p110α as a candidate upstream mediator of the protective effects of melatonin.

Microplastic‐induced ROS have been shown to dysregulate both PI3K/AKT and Nrf2 signaling across multiple organ systems, establishing oxidative pathway disruption as a conserved mechanism of microplastic toxicity [63]. In the present study, PE exposure dose‐dependently suppressed PI3K, AKT1, and GSK3β (Ser9) phosphorylation in the PFC without altering total protein levels, indicating that PE targets pathway activation rather than expression. Reduced AKT1‐mediated inhibitory phosphorylation of GSK3β releases active GSK3β, which phosphorylates Nrf2 and promotes its Keap1‐independent proteasomal degradation [64]. Because PE simultaneously upregulated Keap1, Nrf2 was suppressed through two complementary arms, enhanced Keap1‐dependent ubiquitylation and increased GSK3β‐mediated degradation, resulting in coordinated downregulation of GCLC, NQO1, and HO‐1, GSH depletion, and weakened detoxification capacity [65]. This dual suppression is particularly consequential in the developing brain, where Nrf2 not only governs antioxidant defense but also restrains neuroinflammation by suppressing pro‐inflammatory cytokine expression and modulating NF‐κB signaling [66]. Consistent with this, PE‐exposed mice showed elevated prefrontal IL‐6, IL‐1β, TNF‐α, and MDA alongside reduced SOD and GSH, and transcriptomic analysis revealed enrichment of IL‐6 production and cytokine‐related pathways. These findings delineate a vicious signaling cycle in which PI3K/AKT1 suppression de‐represses both GSK3β‐mediated Nrf2 degradation and NF‐κB‐driven inflammation, while the resulting oxidative damage further impairs PI3K signaling [67]. Finally, AAV‐mediated PIK3CA knockdown in the PFC markedly attenuated the behavioral and antioxidant benefits of melatonin, including its effects on GSH and Nrf2‐dependent antioxidant proteins. These findings provide functional evidence that PIK3CA signaling contributes to the protective effects of melatonin. However, because the knockdown was incomplete and was not restricted to a defined neural cell population, the present experiments do not establish complete pathway necessity and cannot determine whether these effects are mediated predominantly by excitatory neurons, inhibitory neurons, astrocytes, microglia, or other cellular populations within the PFC. Together, the data support PI3K/AKT1/Nrf2 signaling as an important mechanistic node through which early‐life PE exposure amplifies oxidative‐inflammatory and dopaminergic dysfunction in the vulnerable ADHD‐like brain.

Overall, our findings support a mechanistic framework in which PE exposure aggravates an existing neurodevelopmental vulnerability by suppressing PI3K/AKT1 signaling, reducing inhibitory phosphorylation of GSK3β, enhancing Keap1‐dependent and GSK3β‐dependent repression of Nrf2, and weakening downstream antioxidant defenses. The resulting glutathione depletion, lipid peroxidation, inflammatory activation, neuronal injury, and dopaminergic dysfunction may collectively contribute to the worsening of ADHD like behavioral abnormalities. Melatonin partially interrupts this cascade by restoring PI3K/AKT1/GSK3β/Nrf2 signaling and redox homeostasis.

## 5 Limitations

Several limitations should be acknowledged. First, the clinical evidence was derived from a pilot cross‐sectional cohort with a modest sample size and a single blood measurement. Although several potential sources of variability were reduced through age‐ and sex‐matching, recruitment from the same medical center and study period, standardized sampling procedures, and exclusion of recent medication or nutritional supplement use that could substantially affect immune, oxidative, or lipid‐metabolic profiles, residual confounding remains possible. In particular, habitual dietary intake, including consumption of omega‐3‐rich seafood, household plastic use, socioeconomic status, and indoor microplastic or PE‐containing dust exposure were not quantitatively assessed and therefore could not be incorporated into covariate‐adjusted analyses. Fasting blood collection may reduce short‐term dietary variation but does not account for habitual dietary patterns that can influence baseline EPA and DHA levels. These unmeasured factors may affect both internal microplastic burden and the biochemical profiles examined in this study and should therefore be considered when interpreting the observed associations. Moreover, because circulating microplastic burden and ADHD symptoms were assessed concurrently, reverse causation cannot be excluded. ADHD‐related behavioral characteristics, such as increased hand‐to‐mouth activity, pica‐like oral exploration, altered dietary patterns, or greater outdoor activity, may themselves influence microplastic exposure rather than PE burden contributing to symptom severity. Larger prospective birth cohorts incorporating repeated biological measurements, validated dietary and household exposure assessments, socioeconomic indicators, and direct measurements of indoor microplastic exposure will be required to establish temporality and more comprehensively address residual confounding. Microplastic quantification also remains technically challenging because of the risks of sample contamination, limited sensitivity for smaller particles, and variable recovery across particle sizes and polymer types. Second, the human multi‐omics analyses were based on peripheral blood, which may not fully reflect molecular alterations in the prefrontal cortex or other brain regions relevant to ADHD. Transcriptomic profiling was also restricted to children with severe ADHD, limiting the generalizability of these findings across the full spectrum of symptom severity. In addition, immune‐cell abundance was inferred from expression‐based enrichment rather than measured directly. The Mendelian randomization analysis identified a nominal inverse association between genetically predicted AKT1 expression and ADHD risk; however, this association did not remain statistically significant after correction for multiple testing. Accordingly, the AKT1 MR signal should be regarded as exploratory and supportive of candidate‐gene prioritization rather than as definitive evidence of a protective causal effect. Importantly, this MR framework evaluates genetically proxied gene expression in relation to ADHD susceptibility and does not directly assess PE exposure or determine whether AKT1 mediates the association between PE burden and ADHD. Therefore, the MR findings cannot establish an environmental causal pathway linking PE exposure, AKT1, and ADHD. Validation in larger independent genetic datasets and complementary central nervous system and single‐cell models is warranted. Third, the animal model has inherent limitations. The perinatal nicotine exposure paradigm reproduces selected ADHD‐relevant phenotypes but does not capture the full genetic, developmental, and clinical heterogeneity of human ADHD. The inclusion of the 5‐CSRTT enabled direct assessment of attentional performance and impulsive action and therefore strengthened the behavioral relevance of the model. Nevertheless, no single rodent task fully reproduces the multidimensional clinical manifestations of ADHD. Several additional outcomes examined in this study, including marble burying, central‐zone exploration, social interaction, and spatial learning, are not specific to ADHD and should be interpreted as associated neurobehavioral phenotypes rather than core diagnostic features. Moreover, hyperactivity and other dimensions of response inhibition were not comprehensively characterized, and potential sex‐dependent effects were not systematically evaluated. Replication in complementary genetic ADHD models, inclusion of female offspring, and assessment of additional executive‐function domains would further strengthen the translational relevance of the findings. Fourth, the mechanistic resolution remains incomplete. Although additional striatal analyses demonstrated alterations in TH, DRD1, PI3K/AKT1/GSK3β phosphorylation, and Keap1/Nrf2 signaling that were broadly consistent with those observed in the PFC, the deeper mechanistic analyses remained predominantly focused on the PFC. Transcriptomic profiling, CETSA, and AAV‐mediated PIK3CA knockdown were performed primarily in this region; therefore, the present study cannot determine the relative contributions of prefrontal and striatal abnormalities or establish circuit‐level interactions between these regions. Moreover, AAV‐mediated PIK3CA knockdown in the PFC was partial and was not cell‐type specific. Consequently, although the knockdown experiments provide functional evidence that reduced PIK3CA signaling weakens the protective effects of melatonin, they do not establish complete pathway necessity and cannot identify the specific neuronal or glial populations responsible for the observed behavioral and antioxidant effects. In particular, the relative contributions of excitatory neurons, inhibitory neurons, astrocytes, and microglia remain unresolved. Future studies incorporating region‐specific and cell‐type‐specific viral manipulation, Cre‐dependent approaches, conditional PIK3CA models, circuit tracing, and electrophysiological analyses will be required to define the neural populations and circuit‐level mechanisms underlying these effects. Finally, melatonin was evaluated at a single dose using a concurrent preventive administration schedule. Because melatonin was administered throughout the same E1.5‐P21 period as nicotine and PE exposure, the present design primarily evaluates its protective effects against the development or aggravation of PE‐associated abnormalities rather than its therapeutic efficacy after establishment of an ADHD‐like phenotype. No post‐exposure or post‐symptom treatment arm was included; therefore, whether melatonin can reverse established behavioral and molecular abnormalities remains unknown. Future studies should evaluate delayed melatonin administration initiated after cessation of PE exposure or after ADHD‐like phenotypes have become established, together with its pharmacokinetic profile, brain exposure, optimal dosing window, circadian effects, receptor dependence, and long‐term safety. The mechanisms underlying the apparent discordance between reduced PIK3CA and AKT1 transcript levels and relatively stable total PI3K and AKT1 protein abundance were also not directly examined. This discrepancy may reflect differences in protein stability, translational regulation, degradation rates, or compensatory feedback mechanisms and warrants further investigation. Despite these limitations, the convergence of human exposure data, multi‐omics signatures, animal phenotypes, biochemical measurements, pathway activation, and AAV‐mediated PI3KCA knockdown provides a coherent mechanistic framework linking early‐life PE exposure to impaired antioxidant signaling and aggravated ADHD‐like abnormalities.

## 6 Conclusions

In summary, this study provides cross‐species evidence linking circulating PE microplastic burden with ADHD‐related clinical and biological features in children and demonstrates in a PNE mouse model that developmental PE co‐exposure aggravates behavioral, molecular, and metabolic abnormalities associated with impaired PI3K/AKT1/Nrf2 signaling, weakened antioxidant defenses, disrupted lipid homeostasis, neuroinflammation, and altered dopamine homeostasis. Concurrent melatonin administration partially attenuated these abnormalities by restoring PI3K/AKT1/Nrf2 signaling, whereas AAV‐mediated PIK3CA knockdown markedly weakened its protective effects, supporting a functional contribution of PIK3CA signaling to melatonin‐associated neuroprotection. Collectively, the human findings support an association between circulating PE burden and ADHD‐related neurodevelopmental vulnerability, while the animal experiments provide mechanistic evidence that developmental PE exposure can aggravate ADHD‐like phenotypes in a susceptible background. These findings highlight PI3K/AKT1/Nrf2 signaling as an important mechanistic pathway relevant to microplastic‐associated developmental neurotoxicity and preventive neuroprotection.

## Author Contributions

**Yufan Luo**: conceptualization, methodology, software, data curation, investigation, validation. **Ruijin Xie**: methodology, conceptualization, software, data curation, investigation, validation. **Hua Xu**: conceptualization, methodology, software, data curation, investigation, validation. **Xue Xiao**: conceptualization, methodology, software, data curation, investigation, validation. **Wei Xiao**: validation, formal analysis, investigation. **Qiyang Pan**: investigation, validation, formal analysis. **Shengjie Xu**: investigation, validation, formal analysis. **Li Liu**: investigation, validation, formal analysis. **Zeliang Xiao**: software, methodology, conceptualization. **Yue Zhou**: conceptualization, methodology, software. **Chenyu Sun**: visualization, writing – original draft, writing – review and editing, conceptualization, methodology, software, resources. **Yu Wu**: conceptualization, investigation, writing – review and editing, software, data curation, supervision, resources, writing – original draft. **Yueying Liu**: project administration, writing – review and editing, funding acquisition, writing – original draft, visualization, resources, data curation, software, methodology, conceptualization.

## Conflicts of Interest

The authors declare no conflicts of interest.

## Supporting information




**Supporting File**: advs78594‐sup‐0001‐SuppMat.docx.

## Data Availability

The data that support the findings of this study are available from the corresponding author upon reasonable request.
