# Supplementary material for: Early‐Life Polyethylene Microplastic Exposure Exacerbates ADHD‐Like Behaviors in Mice via PI3K/AKT1/Nrf2 Signaling: Evidence From the Pilot Cohort and Multi‐Omics Analyses
Source: Adv Sci (Weinh). 2026 Nov 1:e78594. Online ahead of print. doi: 10.1002/advs.78594 (PMC13723744; doi:10.1002/advs.78594)
Supplement: Supplementary file 1 [file ADVS-9999-e78594-s001.docx]

**Supplemental Questionnaire Section**

**Q1.DSM-5 Diagnostic Criteria for Attention-Deficit/Hyperactivity Disorder (ADHD)^[1]^**

Core Diagnostic Criteria (A–E)

A. Core Symptom Requirement

A persistent pattern of inattention and/or hyperactivity-impulsivity that interferes with functioning or development, as characterized by (1) and/or (2):

1. Inattention: ≥6 symptoms (≥5 for adolescents ≥17 years and adults) for ≥6 months, inconsistent with developmental level, and present in ≥2 settings.
   1. Often fails to give close attention to details or makes careless mistakes in schoolwork, work, or other activities.
   2. Often has difficulty sustaining attention in tasks or play activities.
   3. Often does not seem to listen when spoken to directly.
   4. Often does not follow through on instructions and fails to finish schoolwork, chores, or duties in the workplace (not due to oppositional behavior or failure to understand instructions).
   5. Often has difficulty organizing tasks and activities.
   6. Often avoids, dislikes, or is reluctant to engage in tasks that require sustained mental effort (such as schoolwork or homework).
   7. Often loses things necessary for tasks or activities (e.g., school materials, pencils, books, tools, wallets, keys, paperwork, eyeglasses, mobile telephones).
   8. Is often easily distracted by extraneous stimuli (for older adolescents and adults, may include unrelated thoughts).
   9. Is often forgetful in daily activities (e.g., doing chores, running errands; for older adolescents and adults, returning calls, paying bills, keeping appointments).
2. Hyperactivity-Impulsivity: ≥6 symptoms (≥5 for adolescents ≥17 years and adults) for ≥6 months, inconsistent with developmental level, and present in ≥2 settings.
   1. Hyperactivity:
      1. Often fidgets with or taps hands or feet, or squirms in seat.
      2. Often leaves seat in situations when remaining seated is expected (e.g., in classroom, office).
      3. Often runs about or climbs in situations where it is inappropriate (adolescents or adults may be limited to feeling restless).
      4. Often unable to play or engage in leisure activities quietly.
      5. Is often “on the go,” acting as if “driven by a motor” (e.g., is unable to be or uncomfortable being still for extended time, as in restaurants, meetings; may be experienced by others as being restless or difficult to keep up with).
   2. Impulsivity:
      1. Often blurts out an answer before a question has been completed (e.g., completes people’s sentences; cannot wait for turn in conversation).
      2. Often has difficulty waiting his or her turn (e.g., while waiting in line).
      3. Often interrupts or intrudes on others (e.g., butts into conversations, games, or activities; may start using other people’s things without asking or receiving permission; for adolescents and adults, may intrude into or take over what others are doing).

B. Onset

Several inattentive or hyperactive-impulsive symptoms were present prior to age 12 yearsAPA.

C. Functional Impairment

Symptoms are present in two or more settings (e.g., school, work, home, with peers) and interfere with or reduce the quality of social, academic, or occupational functioning.

D. Exclusion of Other Conditions

Symptoms do not occur exclusively during the course of schizophrenia or another psychotic disorder and are not better explained by another mental disorder (e.g., mood disorder, anxiety disorder, dissociative disorder, personality disorder, substance intoxication or withdrawal).

Presentations

1. Combined Presentation: Meets criteria for both inattention (A1) and hyperactivity-impulsivity (A2) currently.
2. Predominantly Inattentive Presentation: Meets criteria for inattention (A1) but not hyperactivity-impulsivity (A2) currently.
3. Predominantly Hyperactive-Impulsive Presentation: Meets criteria for hyperactivity-impulsivity (A2) but not inattention (A1) currently.

**Q2. SNAP-IV-18 English Version (Parent/Teacher Rating Scale)**

**Instructions:**

Rate how often the child/adolescent exhibits each behavior over the past 6 months using a 4-point scale: 0 = Not at all; 1 = Just a little; 2 = Quite a bit; 3 = Very much.

1. **Inattention Subset (Items 1–9)**
1. Often fails to give close attention to details or makes careless mistakes in schoolwork, work, or other activities.
2. Often has difficulty sustaining attention in tasks or play activities.
3. Often does not seem to listen when spoken to directly.
4. Often does not follow through on instructions and fails to finish schoolwork, chores, or duties in the workplace (not due to oppositional behavior or failure to understand instructions).
5. Often has difficulty organizing tasks and activities.
6. Often avoids, dislikes, or is reluctant to engage in tasks that require sustained mental effort (e.g., schoolwork, homework; for older adolescents/adults, preparing reports, completing forms, reviewing lengthy papers).
7. Often loses things necessary for tasks or activities (e.g., school materials, pencils, books, tools, wallets, keys, paperwork, eyeglasses, mobile phones).
8. Is often easily distracted by extraneous stimuli (for older adolescents/adults, may include unrelated thoughts).
9. Is often forgetful in daily activities (e.g., doing chores, running errands; for older adolescents/adults, returning calls, paying bills, keeping appointments).

**II. Hyperactivity-Impulsivity Subset (Items 10–18)**

10. Often fidgets with or taps hands or feet, or squirms in seat.
11. Often leaves seat in situations when remaining seated is expected (e.g., classroom, office, other work settings, or situations requiring staying in place).
12. Often runs about or climbs in situations where it is inappropriate (adolescents/adults may be limited to feeling restless).
13. Often unable to play or engage in leisure activities quietly.
14. Is often “on the go,” acting as if “driven by a motor” (e.g., unable to be still for extended periods, such as in restaurants, meetings; may be perceived by others as restless or hard to keep up with).
15. Often talks excessively.
16. Often blurts out an answer before a question has been completed (e.g., completes others’ sentences; cannot wait for turn in conversation).
17. Often has difficulty waiting his or her turn (e.g., while waiting in line).
18. Often interrupts or intrudes on others (e.g., butts into conversations, games, or activities; may start using others’ things without asking; for adolescents/adults, may intrude into or take over what others are doing).

**Q3: Conners 3 Parent Rating Scale (PRS) Short Form (43 items, English version)^[2]^**

**Instructions:**

Rate your child’s behavior in the past month using a 4-point scale:

0 = Not True at All (Never/Seldom); 1 = Just a Little True (Occasionally); 2 = Pretty Much True (Often/Quite a Bit); 3 = Very Much True (Very Often/Frequently).

Respond to every item; guess if unsure.

| **Item #** | **Item Content (English)** | **Subscale / Index** |
| --- | --- | --- |
| 1 | Forgets to turn in completed work | Inattention |
| 2 | Is perfect in every way | Positive Impression (Validity) |
| 3 | Fidgets or squirms in seat | Hyperactivity-Impulsivity |
| 4 | Is one of the last to be picked for teams or games | Peer Relations |
| 5 | Cannot do things right | Negative Impression (Validity) |
| 6 | Blurts out answers before questions are completed | Hyperactivity-Impulsivity |
| 7 | Loses things necessary for tasks (e.g., pencils, books) | Inattention |
| 8 | Argues with adults | Oppositional/Defiant |
| 9 | Is good at memorizing facts | Positive Impression (Validity) |
| 10 | Does not get invited to play or go out with others | Peer Relations |
| 11 | Has forced someone into sexual activity | Critical Item (Safety Risk) |
| 12 | Has trouble staying focused on one thing at a time | Inattention |
| 13 | Leaves seat in situations where staying seated is expected | Hyperactivity-Impulsivity |
| 14 | Is cruel to animals | Aggression |
| 15 | Never lies | Positive Impression (Validity) |
| 16 | Difficulty waiting for his/her turn | Hyperactivity-Impulsivity |
| 17 | Avoids tasks requiring sustained mental effort | Inattention |
| 18 | Refuses to follow rules at home | Oppositional/Defiant |
| 19 | Is well-liked by peers | Positive Impression (Validity) |
| 20 | Bullies or threatens others | Aggression |
| 21 | Has trouble organizing tasks or activities | Executive Function |
| 22 | Talks excessively | Hyperactivity-Impulsivity |
| 23 | Has destroyed others’ property | Aggression |
| 24 | Always follows directions | Positive Impression (Validity) |
| 25 | Does not seem to listen when spoken to directly | Inattention |
| 26 | Often “on the go” or acts as if “driven by a motor” | Hyperactivity-Impulsivity |
| 27 | Hits, kicks, or bites others | Aggression |
| 28 | Never gets into trouble | Positive Impression (Validity) |
| 29 | Fails to finish things he/she starts (not due to defiance) | Inattention |
| 30 | Has trouble concentrating | Inattention |
| 31 | Tells the truth; does not even tell “little white lies” | Positive Impression (Validity) |
| 32 | Has trouble organizing tasks or activities | Executive Function |
| 33 | Interrupts or intrudes on others (e.g., butts into conversations) | Hyperactivity-Impulsivity |
| 34 | Steals things of value | Aggression/Conduct |
| 35 | Is easily distracted by noises or activities | Inattention |
| 36 | Climbs or runs in inappropriate situations | Hyperactivity-Impulsivity |
| 37 | Hurts others on purpose | Aggression |
| 38 | Never makes mistakes | Positive Impression (Validity) |
| 39 | Difficulty sustaining attention in tasks or play | Inattention |
| 40 | Refuses to do what adults ask | Oppositional/Defiant |
| 41 | Has trouble waiting for his/her turn | Hyperactivity-Impulsivity |
| 42 | Is disliked by peers | Peer Relations |
| 43 | Cannot pay attention to details; makes careless mistakes | Inattention |

**References:**

1.	Koutsoklenis A, Honkasilta J. ADHD in the DSM-5-TR: What has changed and what has not. **Front Psychiatry** **2022**, 13**:** 1064141.

2.	Kim Y, Kim B, Chang JS, Kim BN, Cho SC, Hwang JW. Parental quality of life and depressive mood following methylphenidate treatment of children with attention-deficit hyperactivity disorder. **Psychiatry Clin Neurosci** **2014**, 68(7)**:** 506–514.

**Supplemental Method Section**

**S1: PPI Network Construction and Hub Gene Identification**

To identify potential molecular targets linking polyethylene (PE) microplastic exposure to attention-deficit/hyperactivity disorder (ADHD), ADHD-related genes were retrieved from the GeneCards database, whereas PE-associated genes were obtained from the Comparative Toxicogenomics Database (CTD) based on our previous studies^[1-3]^. The overlapping genes between the two datasets were identified using Venn analysis and were considered candidate targets potentially involved in the association between PE exposure and ADHD.The candidate genes were subsequently imported into the STRING database to construct a protein-protein interaction (PPI) network. The organism was restricted to Homo sapiens, and interactions with a confidence score greater than 0.4 were retained. The resulting PPI network was imported into Cytoscape for visualization and topological analysis.

Hub genes were identified using the cytoHubba plugin in Cytoscape. Three complementary topological algorithms, including maximal clique centrality (MCC), edge percolated component (EPC), and degree, were independently applied to rank the candidate genes. The top-ranked genes identified by each algorithm were extracted, and genes shared among all three methods were determined by Venn analysis. These overlapping genes were defined as robust hub genes potentially involved in PE-associated ADHD pathophysiology and were subsequently subjected to Mendelian randomization, drug screening, pathway enrichment, and experimental validation analyses.

**S2: Mendelian randomization analysis**

To investigate the potential causal effects of genetically predicted expression of the candidate target genes on ADHD risk, a two-sample Mendelian randomization (MR) analysis was performed. Summary-level genome-wide association study (GWAS) data for ADHD were obtained from the Psychiatric Genomics Consortium (PGC)^[4]^, comprising a large European-ancestry cohort of ADHD cases and controls (Table S4). Expression quantitative trait locus (eQTL) datasets for the candidate genes were obtained from the IEU OpenGWAS database, and the corresponding dataset identifiers and sample information are provided in Table S5^[5]^. For each candidate gene, single-nucleotide polymorphisms (SNPs) associated with gene expression at P < 1 × 10⁻⁵ were initially selected as candidate instrumental variables to retain sufficient genetic instruments for downstream analyses. Linkage disequilibrium clumping was subsequently performed using an r² threshold of <0.001 within a 10,000-kb window to obtain approximately independent instruments.

Instrument strength was assessed using the F statistic, calculated as F= (βexposure/SEexposure)^2^, with F >10 considered indicative of adequate instrument strength. F statistics were used as an instrument-strength diagnostic and were reported for each candidate gene, including the minimum, mean, and median F statistics and the number of variants with F ≤10. Variants were not excluded from the primary analysis solely on the basis of a post-hoc F-statistic threshold. Exposure and outcome datasets were harmonized to ensure consistency of effect alleles, and variants that could not be reliably harmonized, including ambiguous palindromic variants or variants with incompatible alleles, were excluded. Proxy SNPs were not used when exposure-associated variants were unavailable in the ADHD outcome dataset.

For genes with two or more valid instrumental variants, the inverse-variance weighted (IVW) method was prespecified as the primary MR estimator. For genes represented by a single instrumental variant, the Wald ratio method was used. When at least three valid instrumental variants were available, weighted median and MR-Egger regression were additionally conducted as complementary sensitivity analyses. Thus, each candidate gene contributed a single prespecified primary MR estimate, derived from IVW when ≥2 SNPs were available and from the Wald ratio when only one SNP was available. Effect estimates were expressed as odds ratios (ORs) with corresponding 95% confidence intervals (CIs) for ADHD risk per genetically predicted increase in gene expression. Because multiple candidate genes were evaluated, multiple-testing correction was applied across the prespecified primary MR estimates using the Benjamini–Hochberg false discovery rate (BH-FDR) procedure. An FDR-adjusted P value <0.05 was considered statistically significant.

Potential heterogeneity among SNP-specific causal estimates was assessed using Cochran's Q statistic. Directional horizontal pleiotropy was evaluated using the MR-Egger intercept when at least three instrumental variants were available. Leave-one-out analyses were performed for genes with at least three SNPs to evaluate whether the overall IVW estimate was disproportionately driven by an individual variant. Steiger directionality tests were additionally conducted, where applicable, to assess whether the genetic instruments explained greater variance in gene expression than in ADHD liability. Consistency in the direction and magnitude of effect estimates across the primary and complementary MR estimators was considered when evaluating the robustness of the findings. All analyses were performed in R using the TwoSampleMR package. The MR analysis was intended to prioritize candidate genes whose genetically predicted expression is associated with ADHD risk. It was not designed to test the causal effect of PE exposure itself or to assess mediation of PE-associated effects through these genes.

**S3: Publicly available transcriptomic analysis of GSE159104**

The whole-blood transcriptomic dataset GSE159104 was obtained from the Gene Expression Omnibus (GEO) database and analyzed using R. Raw expression data were normalized to counts per million (CPM), aggregated at the gene level, and transformed as log2(CPM + 1). Repeated measurements from the same participant were averaged to generate a single subject-level expression profile. Lowly expressed and invariant genes were removed before subsequent analyses. Principal component analysis (PCA) was performed to assess overall expression patterns and sample clustering. Differential expression between the ADHD and control groups was analyzed using the limma package with empirical Bayes moderation. The Benjamini–Hochberg method was used to control the false discovery rate (FDR), and differentially expressed genes were defined as those with FDR < 0.05 and an absolute fold change > 1.25. Gene Ontology (GO) and Kyoto Encyclopedia of Genes and Genomes (KEGG) enrichment analyses were subsequently performed using the clusterProfiler package, with FDR < 0.05 considered statistically significant.

**S4: Immune Cell Enrichment Analysis**

To characterize peripheral immune alterations associated with ADHD, immune cell enrichment was estimated from the peripheral blood transcriptomic expression matrix using single-sample gene set enrichment analysis (ssGSEA). The LM22 immune-cell signature matrix, comprising marker genes for 22 human immune cell populations, was used as the reference gene set. After normalization of the gene expression data, ssGSEA was performed to calculate a relative enrichment score for each immune cell type in each individual sample based on our previous study^[6]^. The resulting enrichment scores were compared between the ADHD and healthy control groups to identify immune cell populations associated with ADHD-related peripheral immune alterations. Differences between groups were assessed using the Wilcoxon rank-sum test, with a two-sided P value < 0.05 considered statistically significant. Immune cell populations showing significant between-group differences were visualized using box plots. Because ssGSEA provides relative gene-signature enrichment rather than direct estimates of absolute cell abundance, the derived scores were interpreted as differences in immune-cell-associated transcriptional signatures. These analyses were used to complement the cytokine and transcriptomic findings and to characterize peripheral immune activation associated with ADHD.

**S5: In Silico Drug Screening and Experimental Validation**

In silico drug screening was performed to identify candidate compounds capable of modulating the molecular network linking polyethylene (PE) microplastic exposure to attention-deficit/hyperactivity disorder (ADHD). Based on our previous studies, the common hub genes identified from the PPI network and cytoHubba analyses were submitted to the Drug Signatures Database (DSigDB) through the Enrichr web server based on our previous studies^[7, 8]^. Candidate compounds were ranked according to their enrichment scores and the degree of overlap between compound-associated gene signatures and the submitted hub-gene set. Among the top-ranked candidates, five compounds with established antioxidant or neuroprotective potential were selected for further experimental evaluation: N-acetyl-L-cysteine, resveratrol, melatonin, glutathione, and curcumin. Their chemical structures were obtained from public chemical databases and visualized for presentation.

To compare their neuroprotective activities under oxidative stress conditions, HT22 cells were exposed to 5 mM glutamate to establish an oxidative neuronal injury model and subsequently treated with graded concentrations of each candidate compound. Cell viability was assessed using the Cell Counting Kit-8 (CCK-8) assay according to the manufacturer's instructions. Concentration-response curves were generated, and half-maximal effective concentrations (EC50) were estimated by nonlinear regression. The compound exhibiting the strongest protective activity together with a relatively low EC50 was prioritized for subsequent molecular docking, molecular dynamics simulation, and mechanistic validation. Based on the integrated computational ranking and cellular validation, melatonin was selected as the lead candidate for further investigation of its potential protective effects against PE-aggravated ADHD-like phenotypes and its involvement in PI3K/AKT1/Nrf2 signaling.

**S6: Molecular Docking and Molecular Dynamics Simulation**

Molecular docking was performed to evaluate the potential interactions of melatonin with PIK3CA and AKT1, two key components of the PI3K/AKT1 signaling pathway identified in the preceding multi-omics and network analyses. The three-dimensional structures of PIK3CA and AKT1 were obtained from the Protein Data Bank, and the chemical structure of melatonin was retrieved from the PubChem database. Before docking, protein structures were prepared by removing co-crystallized ligands, nonessential water molecules, and other heteroatoms, followed by the addition of polar hydrogen atoms and assignment of appropriate atomic charges. The melatonin structure was subjected to geometry optimization before docking.

Molecular docking was performed using AutoDock Vina 1.2.0. The docking grid was centered on the corresponding ligand-binding pocket of each target protein and was sufficiently large to cover the predicted active region. Multiple binding conformations were generated for each protein-ligand pair, and the conformation with the lowest predicted binding energy and a reasonable orientation within the binding pocket was selected as the representative binding mode. Protein-ligand interactions, including hydrogen bonds, hydrophobic interactions, and other noncovalent contacts, were visualized using PyMOL and Discovery Studio Visualizer.

To further evaluate the dynamic stability of the predicted PIK3CA-melatonin and AKT1-melatonin complexes, molecular dynamics simulations were performed using an AMBER force-field-based protocol. The docked complexes were used as the initial structures. Protein and ligand parameters were assigned using the corresponding AMBER-compatible force fields, after which each complex was placed in an explicit water box and neutralized by the addition of appropriate counterions. The systems were subjected sequentially to energy minimization, heating, and equilibration under constant temperature and pressure conditions before production simulation.

Each protein-ligand complex was subsequently subjected to a 100-ns production molecular dynamics simulation under periodic boundary conditions. System temperature and pressure were maintained at approximately 300 K and 1 atm, respectively. Trajectory coordinates were recorded at regular intervals for subsequent structural analyses.

The stability and conformational dynamics of the complexes were evaluated by calculating the root mean square deviation (RMSD), root mean square fluctuation (RMSF), radius of gyration (Rg), solvent-accessible surface area (SASA), and the number of intermolecular hydrogen bonds throughout the simulation. In addition, free-energy landscapes were constructed using RMSD and Rg as collective variables to identify the predominant low-energy conformational states of the PIK3CA-melatonin and AKT1-melatonin complexes. Collectively, these analyses were used to assess the structural stability and persistence of the predicted interactions between melatonin and PIK3CA or AKT1 under dynamic conditions.

**S7: Three-Chamber Social Interaction Test**

The three-chamber social interaction test was performed to assess sociability and social novelty preference using an apparatus consisting of three interconnected chambers that allowed mice to move freely among the compartments based on our previous study^[6]^. The test consisted of three sequential stages. During stage 0 (habituation), each mouse was placed in the center chamber and allowed to freely explore all three empty chambers for 10 min; total movement distance and the number of chamber transitions were recorded to assess general locomotor activity and spontaneous exploration. During stage 1 (sociability), an unfamiliar age- and sex-matched mouse, designated stranger 1 (S1), was enclosed in a wire cage in one side chamber, while an identical empty wire cage (E) was placed in the opposite chamber. The experimental mouse freely explored the apparatus for 10 min, and sniffing times directed toward S1 and E were recorded. The sociability preference index was calculated as (TS1 − TE)/(TS1 + TE) × 100%. During stage 2 (social novelty), the previously encountered S1 remained in its original cage as the familiar mouse, and a second unfamiliar mouse (Stranger 2, S2) was introduced into the opposite cage. Sniffing times toward S1 and S2 were recorded over 10 min, and the social novelty preference index was calculated as (TS2 − TS1)/(TS2 + TS1) × 100%. A higher positive index indicated greater sociability or social novelty preference, whereas values approaching zero or becoming negative indicated impaired social preference. Behavior was recorded and analyzed using video-tracking software.

**S8: Five-choice serial reaction time task**

The five-choice serial reaction time task (5-CSRTT) was used to assess ADHD-related core behavioral domains, particularly sustained attention and impulsivity. Mice were habituated to the operant chambers and progressively trained to respond to brief visual stimuli presented randomly in one of five apertures. Training was continued until stable task performance was achieved before behavioral testing. Each test session consisted of 100 trials. To evaluate attentional performance, stimulus duration was progressively shortened to 0.8, 0.6, 0.4, and 0.2 s, thereby increasing attentional demand. Accuracy was calculated as correct/(correct + incorrect) × 100%, and omission rate as omissions/total trials × 100%. Impulsivity was assessed using an inter-trial interval (ITI) challenge, in which the ITI was varied across 5, 7, 9, and 11 s, with longer intervals imposing greater response-inhibition demands. Responses made before stimulus presentation were recorded as premature responses and used as the primary measure of impulsivity. Perseverative responses, defined as additional responses made after a correct response, were also recorded as an auxiliary measure of repetitive responding. Behavioral data were analyzed using group and stimulus duration or ITI as between- and within-subject factors, respectively.

**S9: Open-Field Test**

The open-field test was performed to evaluate spontaneous locomotor activity and anxiety-related behavior. Each mouse was individually placed in the center of an open-field arena (50 × 50 × 40 cm) and allowed to explore freely for 10 min under dim-light conditions based on previous studies^[3, 7]^. The arena was virtually divided into central and peripheral zones. Movements were recorded using an automated video-tracking system. Mean locomotor velocity was used to evaluate general motor activity, whereas the number of central-zone entries and the time spent in the central zone were quantified as indices of anxiety-related behavior, with reduced central-zone exploration interpreted as increased anxiety. The arena was cleaned with 75% ethanol and allowed to dry completely between trials.

**S10: Marble-Burying Test**

The marble-burying test was used to assess repetitive and anxiety-related behavior. Each mouse was individually placed in a standard cage containing approximately 5 cm of fresh bedding, on which 20 glass marbles were evenly arranged in a 4 × 5 grid. After 30 min of free exploration under quiet conditions, the number of buried marbles was recorded; a marble was considered buried when at least two-thirds of its surface was covered by bedding. A greater number of buried marbles was interpreted as increased repetitive or anxiety-related behavior. Fresh bedding was used for each mouse, and marbles and cages were thoroughly cleaned between trials.
**S11: Novel Object Recognition Test**

The novel object recognition (NOR) test was performed to evaluate recognition memory and consisted of habituation, training, and testing phases. During habituation, each mouse freely explored the empty testing arena for 10 min on 5 consecutive days. During training, two identical objects were positioned symmetrically in the arena, and mice freely explored both objects for 10 min. After a retention interval of 5min, one familiar object was replaced with a novel object of different shape and appearance, and mice were allowed to explore for 5 min during the testing phase. The positions of the familiar and novel objects were counterbalanced among mice to minimize spatial bias. Exploration was defined as active sniffing or directing the nose toward the object at close distance; sitting on or climbing over the object was not considered exploratory behavior. Exploration times toward the familiar (F) and novel (N) objects were recorded, and the discrimination index was calculated as (TN − TF)/(TN + TF) × 100%. A higher index indicated stronger novel-object recognition, whereas values approaching zero indicated loss of novel-object preference. Mean running speed during the test was also recorded to exclude locomotor impairment as a confounding factor.

**S12:Morris Water Maze Test**

The Morris water maze was performed to evaluate spatial learning and memory. The apparatus consisted of a circular pool (120 cm in diameter) filled with opaque water maintained at 22 ± 1 °C. The pool was virtually divided into four quadrants, and a circular escape platform was submerged approximately 1 cm below the water surface in the target quadrant. Spatial cues surrounding the pool remained unchanged throughout the experiment. During the acquisition phase, mice underwent four training trials per day for five consecutive days. For each trial, mice were released from different predetermined starting positions and allowed a maximum of 60 s to locate the hidden platform; mice that failed to find the platform within 60 s were gently guided to it and allowed to remain for 15 s. Escape latency and swimming trajectories were recorded throughout training. Twenty-four hours after the final training session, the platform was removed and a 60-s probe trial was performed. Spatial memory retention was evaluated by the number of crossings over the former platform location and the time spent in the target quadrant. Representative swimming trajectories were additionally analyzed to characterize search strategies. Mean swimming speed was recorded to exclude motor performance differences as a potential explanation for between-group differences in spatial learning and memory. Behavior was recorded and analyzed using video-tracking software.

**S13: Nissl Staining and Histological Analysis**

Nissl staining was performed to evaluate hippocampal neuronal injury in the different experimental groups. After completion of the behavioral tests, brain tissues were collected and fixed in 4% paraformaldehyde, followed by routine dehydration, paraffin embedding, and sectioning at a thickness of 4 μm. Sections containing the hippocampus were deparaffinized, rehydrated through a graded ethanol series, and stained with cresyl violet according to standard procedures.

The hippocampal CA1 and CA3 regions were examined under a light microscope, and representative images were acquired under identical imaging conditions. Morphologically intact neurons were characterized by relatively regular cell bodies, clear nuclei, and abundant Nissl substance, whereas injured neurons exhibited cellular shrinkage, disorganized arrangement, and reduced or fragmented Nissl staining. Neuronal density in the CA1 and CA3 regions was quantified using ImageJ software. Equivalent anatomical regions and comparable fields of view were selected across experimental groups to minimize sampling bias. Quantification and image analysis were performed by investigators blinded to group allocation. The results were used to assess whether early-life polyethylene microplastic exposure aggravated hippocampal neuronal injury in ADHD-like mice and whether melatonin treatment attenuated these pathological alterations.

**S14 Cellular Thermal Shift Assay for PIK3CA Target Engagement**

A cellular thermal shift assay (CETSA) was performed to evaluate the thermal stability of the PI3K catalytic subunit p110α (PIK3CA) in prefrontal cortex (PFC) tissue. Fresh PFC samples were collected from the ADHD + High PE and ADHD + High PE + Mel groups and homogenized in ice-cold phosphate-buffered saline supplemented with protease inhibitors. Homogenates were centrifuged at 12,000 × g for 15 min at 4 °C, and the resulting supernatants were collected and divided into equal aliquots.

Aliquots from each sample were heated at 45, 50, 55, 60, and 65 °C for 3 min and then allowed to cool at room temperature for 3 min. Samples were subsequently centrifuged at 20,000 × g for 20 min at 4 °C to remove heat-denatured and precipitated proteins. The soluble fractions were collected and analyzed by Western blotting using a PIK3CA/p110α-specific antibody (PI3 kinase p110α, Cell Signaling Technology, cat. no. 4249S). Equal volumes of soluble protein were loaded for each temperature point, and PIK3CA band intensities were quantified using ImageJ software. For each sample, the soluble PIK3CA signal at each temperature was normalized to the corresponding signal at 45 °C and expressed as the percentage of soluble PIK3CA remaining. Thermal stability curves were generated by plotting normalized soluble PIK3CA abundance against temperature. Greater retention of soluble PIK3CA at elevated temperatures in the ADHD + High PE + Mel group relative to the ADHD + High PE group was interpreted as melatonin-associated stabilization of the p110α catalytic subunit. These CETSA findings were considered together with molecular docking, molecular dynamics simulations, and AAV-mediated PIK3CA knockdown experiments as evidence consistent with potential PIK3CA target engagement by melatonin. CETSA was not interpreted as definitive evidence of direct molecular binding.

**S15 Western Blot Analysis**

Western blotting was performed to evaluate proteins involved in dopaminergic signaling, neurotrophic support, and the PI3K/AKT1/GSK3β/Nrf2 pathway. Prefrontal cortex or hippocampal tissues were homogenized in ice-cold RIPA lysis buffer supplemented with protease and phosphatase inhibitors. Lysates were centrifuged at 12,000 × g for 15 min at 4 °C, and the supernatants were collected. Protein concentrations were determined using a bicinchoninic acid assay. Equal amounts of protein were separated by SDS-PAGE and transferred onto polyvinylidene difluoride membranes. Membranes were blocked with 5% nonfat milk or bovine serum albumin for 1 h at room temperature and incubated overnight at 4 °C with the appropriate primary antibodies. The proteins examined included phospho-PI3 kinase p85 (Tyr458), total PI3 kinase p85, phospho-AKT1 (Ser473), total AKT1, phospho-GSK3β (Ser9), total GSK3β, Nrf2, Keap1, tyrosine hydroxylase (TH), dopamine receptor D1 (DRD1), brain-derived neurotrophic factor (BDNF), and β-actin. Antibody catalog numbers and manufacturers are provided in the Chemicals and Reagents section. In the striatum, TH and DRD1 were assessed as markers of dopaminergic signaling, together with phospho-PI3K p85, total PI3K p85, phospho-AKT1, total AKT1, phospho-GSK3β, total GSK3β, Keap1, and Nrf2 to determine whether the molecular alterations observed in the PFC also extended to this ADHD-relevant dopaminergic region. To distinguish pathway activation from catalytic-subunit target validation, the PI3K p85 regulatory subunit and its Tyr458 phosphorylation were quantified to assess PI3K pathway activation, and phospho-PI3K levels were normalized to total PI3K p85. In contrast, the PI3K catalytic subunit p110α (PIK3CA) was specifically examined using a p110α-specific antibody in the CETSA experiments and for validation of AAV-mediated PIK3CA knockdown. Thus, the p85 regulatory subunit was used as a signaling readout, whereas p110α/PIK3CA was used for catalytic-subunit-specific target and genetic validation.

After washing with TBST, membranes were incubated with the corresponding horseradish peroxidase-conjugated secondary antibodies for 1 h at room temperature. Protein bands were visualized using an enhanced chemiluminescence detection system and quantified using ImageJ software. β-Actin was used as the loading control for total protein measurements. Phosphorylated protein levels were normalized to their corresponding total protein levels and expressed as p-PI3K/PI3K, p-AKT1/AKT1, and p-GSK3β/GSK3β ratios. All densitometric analyses were performed using identical exposure conditions.

**S16 AAV-Mediated PI3K Knockdown**

To determine whether PI3K signaling was required for the protective effects of melatonin, adeno-associated virus-mediated PI3K knockdown was performed in the prefrontal cortex. Three independent short hairpin RNA constructs targeting PI3K were designed and packaged into AAV vectors (Table S7), together with a corresponding scrambled shRNA control. The three candidate constructs were initially screened for knockdown efficiency by western blotting, and the construct producing the greatest reduction in PI3K protein expression was selected for subsequent in vivo experiments. For stereotaxic injection, mice were anesthetized and fixed in a stereotaxic apparatus. After exposure of the skull, bilateral injections were performed into the prefrontal cortex using coordinates referenced to bregma according to the mouse brain atlas. AAV suspension was slowly delivered using a microsyringe, and the needle was retained in place for several minutes after injection to minimize reflux. Mice were allowed to recover for an appropriate period to permit stable viral expression before behavioral and biochemical analyses.The viral strategy was designed to reduce PIK3CA expression within the PFC but was not restricted to a specific neuronal or glial cell population; therefore, the experiment was intended to assess the regional contribution of PIK3CA signaling rather than cell-type-specific function.

The main experimental groups used for mechanistic validation included ADHD + High PE, ADHD + High PE + Melatonin, and ADHD + High PE + Melatonin + AAV-shPI3K groups, together with the corresponding controls. General locomotor activity was first assessed during stage 0 of the three-chamber test to exclude nonspecific motor effects of viral intervention. Sociability and social novelty preference were then evaluated to determine whether PI3K knockdown altered the behavioral rescue produced by melatonin. After behavioral testing, prefrontal cortex tissues were collected to assess PI3K expression and downstream antioxidant responses. GSH levels and the expression of NQO1, GCLC, and HO-1 were quantified. The extent to which PI3K knockdown attenuated melatonin-mediated restoration of these behavioral and antioxidant endpoints was used to evaluate the functional requirement of PI3K signaling in the protective effects of melatonin.

**S17 RT-qPCR Analysis**

RT-qPCR was performed to validate transcriptomic findings and evaluate the expression of genes associated with Nrf2-dependent antioxidant signaling. Total RNA was extracted from peripheral blood or mouse prefrontal cortex tissue using a commercial RNA extraction kit according to the manufacturer's instructions. RNA concentration and purity were assessed spectrophotometrically, and equivalent amounts of RNA were reverse-transcribed into complementary DNA using a reverse-transcription kit. Quantitative PCR was performed using SYBR Green-based detection on a real-time PCR system. The amplification protocol consisted of an initial denaturation step followed by 40 amplification cycles and melt-curve analysis to confirm amplification specificity. Relative gene expression was calculated using the 2^-ΔΔCt method after normalization to the corresponding housekeeping gene.

For human peripheral blood validation, the expression of PIK3CA, PIK3R1, AKT1, NFE2L2, and GCLC was analyzed. In mouse prefrontal cortex tissue, RT-qPCR was primarily used to quantify Nfe2l2 and its downstream antioxidant genes, including Gclc, Nqo1, and Hmox1. Primer sequences are provided in Table S5 All reactions were performed in technical triplicate, and the mean Ct value was used for subsequent analysis.

**References:**

1.	Han P, Chu S, Shen J, Li L, Zhang Y, Wang S*, et al.* Quercetin-derived microbial metabolite DOPAC potentiates CD8(+) T cell anti-tumor immunity via NRF2-mediated mitophagy. **Cell Metab** **2025**, 37(12)**:** 2438–2454.e2438.

2.	Xie R, Xiao X, Zhao W, Zhong Y, Wu D, Dou J*, et al.* Association between long-term exposure of polystyrene microplastics and exacerbation of seizure symptoms: Evidence from multiple approaches. **Ecotoxicol Environ Saf** **2025**, 302**:** 118741.

3.	Liu Y, Xie R, Zhao W, Xu H, Dou J, Xiao X*, et al.* Long-term exposure to polystyrene microplastics exacerbates seizure symptoms via lipid metabolic disruption and ferroptosis: insights from multi-omics analyses. **J Nanobiotechnology** **2026**.

4.	Agrawal A, Bulik CM, Abebe DS, Andreassen OA, Atkinson EG, Choi KW*, et al.* The Psychiatric Genomics Consortium: discoveries and directions. **Lancet Psychiatry** **2025**, 12(8)**:** 600–610.

5.	Tuersong T, Yong YX, Chen Y, Li PS, Shataer S, Shataer M*, et al.* Integrating plasma circulating protein-centered multi-omics to identify potential therapeutic targets for Parkinsonian cognitive disorders. **J Transl Med** **2025**, 23(1)**:** 535.

6.	Dou J, Zhang K, Xie R, Xu H, Pan Q, Xiao X*, et al.* Investigating the Effects of Long-Term Fine Particulate Matter Exposure on Autism Spectrum Disorder Severity: Evidence from Multiple Analytical Approaches. **Toxics** **2025**, 13(11).

7.	Jiang Y, Xie R, Cao Y, Zhao W, Shen J, Xu H*, et al.* Resveratrol alleviates epilepsy-induced brain damage via the P62-Keap1-Nrf2 Pathway: A multi-omics study integrating the pilot cohort, In silico analyses, and experimental validation. **Free Radic Biol Med** **2026**, 254**:** 788–814.

8.	Chang Y, Jiang X, Dou J, Xie R, Zhao W, Cao Y*, et al.* Investigating the potential risk of cadmium exposure on seizure severity and anxiety-like behaviors through the ferroptosis pathway in epileptic mice: An integrated multi-omics approach. **J Hazard Mater** **2024**, 480**:** 135814.

**Supplemental Figure Section**

**Figure S1.** Representative micro-Raman spectroscopic identification of additional microplastic polymer types detected in peripheral blood samples. Representative microscopic images (left column), high-resolution enlarged images (middle column), and corresponding Raman spectra (right column) are shown for polyethylene terephthalate (PET), polyvinyl chloride (PVC), polyurethane (PU), and polybutylene adipate terephthalate (PBAT) particles (from top to bottom).

**Figure S2.** Subgroup analyses of circulating inflammatory cytokines, antioxidant enzyme activity, and polyunsaturated fatty acid profiles across children with mild, moderate, and severe ADHD. (A–C) Peripheral blood levels of interleukin-1β (IL-1β; A), tumor necrosis factor-α (TNF-α; B), and superoxide dismutase (SOD; C) activity showed no significant differences among the three severity subgroups (n = 15 per subgroup). (D) Arachidonic acid (AA) levels did not differ significantly across subgroups. (E–F) The AA/docosahexaenoic acid (DHA; E) and AA/eicosapentaenoic acid (EPA; F) ratios progressively increased with ADHD symptom severity, with statistically significant differences between mild and moderate (*P < 0.05), moderate and severe (**P < 0.01), and mild and severe (**P < 0.01) subgroups. NS, not significant.

**Figure S3**. Single-sample gene set enrichment analysis (ssGSEA) of peripheral blood immune cell signatures in healthy control children and children with severe ADHD. Enrichment scores for 22 immune cell subsets were derived using the LM22 signature matrix. Box plots depict the ssGSEA scores for each cell type, with individual data points overlaid. Children with severe ADHD showed significantly elevated enrichment signatures for CD8+ T cells, regulatory T cells (Tregs), γδ T cells, and resting and activated mast cells compared with healthy controls (*P < 0.05, **P < 0.01). ns, not significant.

**Figure S4:** Independent transcriptomic analysis of GSE159104 identifies altered redox- and metabolism-related pathways in children with ADHD. (A) Principal component analysis of peripheral blood RNA-sequencing data from 39 children with ADHD and 37 healthy controls in the GSE159104 dataset. (B) Volcano plot showing differentially expressed genes between the ADHD and control groups. Differentially expressed genes were defined using a BH-adjusted P < 0.05 and fold change > 1.25 or < 0.80, identifying 129 upregulated and 132 downregulated genes. (C) Gene Ontology biological process enrichment analysis of differentially expressed genes, highlighting biological processes related to peroxisomal function, nervous system development, protein regulation, and cellular stress responses. (D) KEGG pathway enrichment analysis showing significant enrichment of pathways including peroxisome, proteasome, cytosolic DNA-sensing, allograft rejection, and ribosome-related processes. The enrichment of peroxisome pathway provides additional transcriptomic evidence of disturbed cellular redox and metabolic homeostasis in ADHD, which may intersect with PI3K/AKT-related signaling.

**Figure S5.** PE-only exposure does not significantly alter social behavior, attention, or impulsivity in mice. (A–C) Three-chamber social interaction test in control mice and mice exposed to PE alone at 1 or 3 mg/kg. (A) Total distance traveled during the habituation stage was comparable among groups, indicating preserved general locomotor activity. (B) Sociability preference index and (C) social novelty preference index showed no significant differences among the Control, PE-only Low, and PE-only High groups. (D–G) Performance in the five-choice serial reaction time task (5-CSRTT). (D) Relative accuracy and (E) omission rate were assessed across stimulus durations of 0.8, 0.6, 0.4, and 0.2 s to evaluate attentional performance. (F) Premature responding and (G) perseverative responding were assessed across inter-trial intervals of 5, 7, 9, and 11 s as indices of impulsive and repetitive responding, respectively. No significant group differences were observed across the tested stimulus durations or inter-trial intervals.

**Figure S6.**Five-choice serial reaction time task (5-CSRTT) assessment of attention and impulsivity in ADHD-like mice following early-life polyethylene microplastic exposure and melatonin intervention. (A) Attentional accuracy across progressively shortened stimulus durations (0.8, 0.6, 0.4, and 0.2 s). ADHD-like mice exhibited reduced response accuracy compared with controls, and polyethylene (PE) co-exposure further impaired attentional performance in a dose-dependent manner, whereas melatonin partially attenuated these deficits. (B) Omission rate across the same stimulus-duration conditions. Omission rates increased in ADHD-like mice and were further elevated by PE co-exposure, particularly in the high-dose PE group, while melatonin partially reduced this increase. (C) Premature responding during the impulsivity paradigm with progressively prolonged inter-trial intervals (ITIs; 5, 7, 9, and 11 s). ADHD-like mice showed increased premature responses, which were further exacerbated by PE exposure and partially ameliorated by melatonin. (D) Perseverative responding across the same ITI conditions. PE co-exposure increased perseverative responding in ADHD-like mice, whereas melatonin partially reversed this effect. Groups included Control, ADHD, ADHD + Low PE, ADHD + High PE, and ADHD + High PE + Melatonin. P < 0.05, P < 0.01; N.S., not significant.

**Figure S7:** Dose-dependent effects of early-life polyethylene (PE) microplastic exposure on serum polyunsaturated fatty acid profiles and prefrontal cortical (PFC) cytokine levels in perinatal nicotine exposure (PNE)–induced ADHD-like mice, and their modulation by melatonin. (A–C) Serum levels of arachidonic acid (AA; A) and the AA/EPA (B) and AA/DHA (C) ratios were elevated in ADHD-like mice, further increased by PE co-exposure in a dose-dependent manner, and partially restored by melatonin treatment (30 mg/kg/day). (D–E) PFC levels of the pro-inflammatory cytokines interleukin-1β (IL-1β; D) and tumor necrosis factor-α (TNF-α; E) showed progressive, dose-dependent increases following nicotine and PE exposure, which were attenuated by melatonin. (F) PFC levels of the anti-inflammatory cytokine interleukin-10 (IL-10) were reduced in ADHD-like mice, further decreased by PE exposure, and partially restored by melatonin. **P < 0.01. Data are presented as mean ± standard deviation with individual data points overlaid (n = 8 per group).

**Figure S8:** Early-life PE exposure disrupts striatal dopaminergic signaling and suppresses the PI3K/AKT1/GSK3β/Nrf2 pathway in ADHD-like mice. (A-B) Representative Western blots and quantitative analysis of tyrosine hydroxylase (TH) and dopamine receptor D1 (DRD1) in the striatum. TH and DRD1 protein levels were reduced in ADHD mice and further decreased following PE co-exposure, with a more pronounced reduction in the high-dose PE group. Melatonin partially restored both proteins. (C-D) Representative Western blots and densitometric analysis of the striatal PI3K/AKT1/GSK3β signaling pathway. Phosphorylation of PI3K p85 (Tyr458), AKT1 (Ser473), and GSK3β (Ser9) was reduced in ADHD mice and further suppressed by PE co-exposure, whereas total PI3K, AKT1, and GSK3β protein levels remained largely unchanged. Melatonin partially restored PI3K/AKT1/GSK3β phosphorylation. (E-F) Representative Western blots and quantitative analysis of Keap1 and Nrf2 in the striatum. PE co-exposure increased Keap1 expression and decreased Nrf2 abundance, whereas melatonin partially reversed these alterations. β-Actin was used as the loading control. Phosphorylated proteins were normalized to their corresponding total protein levels, whereas TH, DRD1, Keap1, and Nrf2 were normalized to β-actin. P < 0.05, P < 0.01; N.S., not significant.

**Supplemental Table Section**

Table S1：Demographic and Clinical Characteristics of ADHD Patients

|  | **ADHD Symptoms** | | |  |
| --- | --- | --- | --- | --- |
| **Characteristic** | **Mild**  **(N=15)**^1^ | **Moderate (N=15)**^1^ | **Severe (N=15)**^1^ | **P-value**^2^ |
| **Years** |  |  |  | 0.3 |
| Mean (SD) | 9.26 (1.39) | 8.46 (1.49) | 8.91 (1.40) |  |
| **Sex** |  |  |  | >0.9 |
| Boy | 11 (73%) | 12 (80%) | 11 (73%) |  |
| Girl | 4 (27%) | 3 (20%) | 4 (27%) |  |
| **BMI** |  |  |  | 0.054 |
| Mean (SD) | 16.39 (1.17) | 15.82 (1.13) | 15.47 (0.93) |  |
| **Education** |  |  |  | >0.9 |
| Primary school | 15 (100%) | 15 (100%) | 15 (100%) |  |
| Junior school | 0 (0%) | 0 (0%) | 0 (0%) |  |
| **SNAP-IV** |  |  |  | **<0.001** |
| Mean (SD) | 1.28 (0.08) | 1.82 (0.11) | 2.47 (0.83) |  |
| **Conners** |  |  |  | **<0.001** |
| Mean (SD) | 62.73(2.50) | 69.15(4.37) | 74.81(3.95) |  |
| ^1^n (%) | | | | |
| ^2^Kruskal-Wallis rank sum test; Fisher's exact test | | | | |

**Table S2: Scoring and interpretation of the SNAP-IV-18 average rating per item (ARI)**

| **SNAP-IV-18 measure** | **Items** | **Raw Score Range** | **ARI range** | **Clinical interpretation** |
| --- | --- | --- | --- | --- |
| Inattention (IA) | 1–9 | 0–27 | 0–3 | Higher scores indicate greater inattentive symptom burden. |
| Hyperactivity/Impulsivity (HI) | 10–18 | 0–27 | 0–3 | Higher scores indicate greater hyperactive/impulsive symptom burden. |
| Overall | 1–18 | 0–54 | 0–3 | Higher scores indicate greater overall ADHD symptom burden. |

**Scoring and interpretation:** The SNAP-IV-18 comprises 18 ADHD symptom items corresponding to the Inattention (IA; items 1–9) and Hyperactivity/Impulsivity (HI; items 10–18) domains. Each item is rated by the parent on a four-point Likert scale: 0 = not at all, 1 = just a little, 2 = quite a bit, and 3 = very much.

For each participant, the IA and HI raw scores were obtained by summing the corresponding nine item scores. The combined ADHD raw score was calculated as the sum of all 18 ADHD items. The average rating per item (ARI) was calculated by dividing the summed item score by the number of items included in the corresponding scale**:**

**ARI = Sum of item scores / Number of items**

**Clinical severity classification:** In the present study, mild, moderate, and severe ADHD subgroups were determined independently by qualified pediatric clinicians according to the DSM-5 severity specifier, incorporating symptom burden and functional impairment. The SNAP-IV-18 ARI was not used as a numerical cutoff for assigning participants to these clinical severity groups. Instead, the combined ADHD ARI was analyzed as a continuous quantitative measure of symptom burden and was used to characterize symptom differences across the clinician-defined severity groups.

**Note:** The SNAP-IV is a symptom-rating instrument and should not be used alone to establish an ADHD diagnosis or clinical severity category. Interpretation should be integrated with developmental history, symptom presentation across settings, functional impairment, and comprehensive clinical assessment. The Chinese version of the SNAP-IV has demonstrated satisfactory reliability and validity for assessing ADHD symptoms in pediatric populations..

**Table S3: Scoring and interpretation of Conners-3 standardized T-scores**

| **Conners-3 T-score** | **Standard interpretive range** | **Clinical interpretation** |
| --- | --- | --- |
| <60 | Average or below | Ratings fall within or below the range typically observed in the relevant normative group. |
| 60–64 | High Average | Ratings are somewhat higher than typically observed and may indicate emerging or borderline concerns. |
| 65–69 | Elevated | Ratings indicate a clinically meaningful elevation in the assessed symptom domain and warrant consideration in the context of the overall clinical evaluation. |
| ≥70 | Very Elevated | Ratings indicate a marked elevation in the assessed symptom domain relative to the normative group and warrant careful clinical evaluation. |

**Note:** Conners-3 T-scores are standardized, norm-referenced scores with a mean of 50 and a standard deviation of 10. T-scores are interpreted relative to the appropriate age- and sex-specific normative group for the version of the Conners Rating Scale administered. Higher T-scores indicate a greater level of concern in the corresponding symptom or behavioral domain relative to the normative population. In the present study, Conners-3 T-scores were used as quantitative measures of ADHD-related symptom burden and were not used as fixed numerical criteria to assign participants to mild, moderate, or severe ADHD groups. Clinical ADHD severity was determined independently by qualified pediatric clinicians according to DSM-5 severity criteria, incorporating symptom burden and functional impairment. Conners-3 scores were therefore used to characterize and provide complementary quantitative support for the clinician-defined severity groups.

**Scoring procedure**

Step 1 Calculate the raw score for each scale: Individual item ratings were summed according to the scoring rules of the specific Conners-3 scale or subscale analyzed. Relevant domains may include Inattention, Hyperactivity/Impulsivity, ADHD-related indices, and DSM-oriented symptom scales, depending on the form administered. Raw scores were calculated separately for each scale and were not combined across scales unless explicitly specified by the scoring manual.

Step 2 Select the appropriate normative reference: For each participant, the corresponding normative reference was selected according to the child's age and sex, as specified in the Conners-3 scoring manual. Because Conners-3 T-scores are norm-referenced, identical raw scores may correspond to different T-scores across different age or sex groups.

Step 3 Convert raw scores to standardized T-scores: Raw scores were converted to T-scores using the appropriate normative conversion tables or authorized scoring system for the Conners-3. The standardized T-score metric has a normative mean of 50 and a standard deviation of 10. Higher T-scores indicate greater symptom or behavioral concerns relative to the corresponding normative group.

Step 4 Interpret the standardized T-score: T-scores were interpreted according to the conventional Conners-3 ranges: <60, Average or below; 60–64, High Average; 65–69, Elevated; and ≥70, Very Elevated. These ranges were used to describe the degree of symptom elevation and were not equated directly with DSM-5 mild, moderate, or severe ADHD categories.

**Table S4：Spike-and-Recovery Validation of PE, PP, and PS Microplastics Using the Micro-Raman Analytical Workflow**

| **Polymer** | **Size** | **Spiked particle counts** | **Recovered particle counts** | **Recovery rate, mean ± SD** |
| --- | --- | --- | --- | --- |
| PE | 1 μm | 50 | 34, 36, 37, 35, 38 | **72.0 ± 3.2%** |
| PE | 5 μm | 50 | 42, 44, 43, 45, 42 | **86.4 ± 2.6%** |
| PP | 1 μm | 50 | 32, 35, 34, 33, 37 | **68.4 ± 3.8 %** |
| PP | 5 μm | 50 | 40, 42, 41, 43, 41 | **82.8 ± 2.3%** |
| PS | 1 μm | 50 | 35, 37, 36, 38, 36 | **72.8 ± 2.2%** |
| PS | 5 μm | 50 | 43, 44, 42, 45, 44 | **87.2 ± 2.3%** |

**Table S5. Background Contamination in Reagent, Filter, and Procedural Blanks During Microplastic Analysis**

| **Blank type** | **n** | **PE** | **PP** | **PS** | **PET** | **Other confirmed polymers¹** | **Fibres²** | **Total observed particles** |
| --- | --- | --- | --- | --- | --- | --- | --- | --- |
| Reagent blank | 5 | 0 | 0 | 0.2 ± 0.4 | 0.4 ± 0.5 | 0.4 ± 0.5 | 0.8 ± 0.8 | **1.8 ± 1.1** |
| Filter blank | 5 | 0.2 ± 0.4 | 0 | 0.2 ± 0.4 | 0.4 ± 0.5 | 0.6 ± 0.5 | 1.4 ± 1.1 | **2.8 ± 1.5** |
| Procedural blank | 5 | **0.2 ± 0.4** | 0.2 ± 0.4 | 1.0 ± 0.7 | 1.8 ± 0.8 | 2.0 ± 1.0 | 3.8 ± 2.8 | **9.0 ± 2.9** |

Data are expressed as particles per blank, mean ± SD.

¹ Other confirmed polymers include Raman-confirmed synthetic polymers other than PE, PP, PS, and PET.

² Fibres were recorded separately as potential background particles. Fibres without definitive Raman confirmation of a synthetic polymer were not included in confirmed microplastic counts.

**Table S6：Summary GWAS Data used In this study**

| **GWAS ID** | **Source** | **Trait/Genes** |
| --- | --- | --- |
| ADHD2022 | Psychiatric Genomics Consorium | ADHD symptoms |
| eqtl-a-ENSG00000171791 | IEU Open GWAS | CDH1 |
| eqtl-a-ENSG00000197122 | IEU Open GWAS | TGFB1 |
| eqtl-a-ENSG00000039068 | IEU Open GWAS | NFE2L2 |
| eqtl-a-ENSG00000125538 | IEU Open GWAS | IL1B |
| eqtl-a-ENSG00000136244 | IEU Open GWAS | IL6 |
| eqtl-a-ENSG00000142208 | IEU Open GWAS | AKT1 |
| eqtl-a-ENSG00000148400 | IEU Open GWAS | NOCH1 |
| eqtl-a-ENSG00000164305 | IEU Open GWAS | CAPS3 |
| eqtl-a-ENSG00000170345 | IEU Open GWAS | FOS |
| eqtl-a-ENSG00000171791 | IEU Open GWAS | BCL2 |
| eqtl-a-ENSG00000173039 | IEU Open GWAS | RELA |
| eqtl-a-ENSG00000197122 | IEU Open GWAS | MTOR |
| eqtl-a-ENSG00000232810 | IEU Open GWAS | TNF |

**Table S7：The Specific Primer Used in this Study**

| **Genes** | **Sequence (5' -> 3')** |
| --- | --- |
| **For Human** | |
| H-PIK3CA-Forward | CCACGACCATCATCAGGTGAA |
| H-PIK3CA-Reverse | CCTCACGGAGGCATTCTAAAGT |
| H-PIK3R1-Forward | ACCACTACCGGAATGAATCTCT |
| H-PIK3R1-Reverse | GGGATGTGCGGGTATATTCTTC |
| H-AKT1-Forward | CCTCCACGACATCGCACTG |
| H-AKT1-Reverse | TCACAAAGAGCCCTCCATTATCA |
| H-NFE2L2-Forward | TCAGCGACGGAAAGAGTATGA |
| H-NFE2L2-Reverse | CCACTGGTTTCTGACTGGATGT |
| H-GCLC-Forward | GGAGGAAACCAAGCGCCAT |
| H-GCLC-Reverse | CTTGACGGCGTGGTAGATGT |
| **For Mice** | |
| M-NFE2L2-Forward | CTTTAGTCAGCGACAGAAGGAC |
| M-NFE2L2-Reverse | AGGCATCTTGTTTGGGAATGTG |
| M-GCLC-Forward | CTACCACGCAGTCAAGGACC |
| M-GCLC-Reverse | CCTCCATTCAGTAACAACTGGAC |
| M-NQO1-Forward | AGGATGGGAGGTACTCGAATC |
| M-NQO1-Reverse | TGCTAGAGATGACTCGGAAGG |
| M-HMOX1-Forward | AGGTACACATCCAAGCCGAGA |
| M-HMOX1-Reverse | CATCACCAGCTTAAAGCCTTCT |

**Table S8：The Specific Primer Used in this AAV study**

| **Genes** | **Sequence (5' -> 3')** |
| --- | --- |
| shRNA1-PIK3CA | 5′-ATGTTTACTACCAAATGGA-3′ |
| shRNA2-PIK3CA | 5′-GGTGGAATGAATGGCTGAA-3′ |
| shRNA3-PIK3CA | 5-CTGAGTCAGTATAAGTATATA-3′ |
